# Diversity and distribution of lichen-associated fungi in the Ny-Ålesund Region (Svalbard, High Arctic) as revealed by 454 pyrosequencing

**DOI:** 10.1038/srep14850

**Published:** 2015-10-14

**Authors:** Tao Zhang, Xin-Li Wei, Yu-Qin Zhang, Hong-Yu Liu, Li-Yan Yu

**Affiliations:** 1 China Pharmaceutical Culture Collection, Institute of Medicinal Biotechnology, Chinese Academy of Medical Sciences & Peking Union Medical College, Beijing 100050, PR China; 2 State Key Laboratory of Mycology, Institute of Microbiology, Chinese Academy of Sciences, Beijing 100101, PR China

## Abstract

This study assessed the diversity and distribution of fungal communities associated with seven lichen species in the Ny-Ålesund Region (Svalbard, High Arctic) using Roche 454 pyrosequencing with fungal-specific primers targeting the internal transcribed spacer (ITS) region of the ribosomal rRNA gene. Lichen-associated fungal communities showed high diversity, with a total of 42,259 reads belonging to 370 operational taxonomic units (OTUs) being found. Of these OTUs, 294 belonged to Ascomycota, 54 to Basidiomycota, 2 to Zygomycota, and 20 to unknown fungi. Leotiomycetes, Dothideomycetes, and Eurotiomycetes were the major classes, whereas the dominant orders were Helotiales, Capnodiales, and Chaetothyriales. Interestingly, most fungal OTUs were closely related to fungi from various habitats (e.g., soil, rock, plant tissues) in the Arctic, Antarctic and alpine regions, which suggests that living in association with lichen thalli may be a transient stage of life cycle for these fungi and that long-distance dispersal may be important to the fungi in the Arctic. In addition, host-related factors shaped the lichen-associated fungal communities in this region. Taken together, these results suggest that lichens thalli act as reservoirs of diverse fungi from various niches, which may improve our understanding of fungal evolution and ecology in the Arctic.

The Arctic is one of the most pristine regions of the planet, and its environment exhibits extreme conditions (e.g., low temperature, strong winds, permafrost, and long periods of darkness and light) and offers unique opportunities to explore extremophiles. Unfortunately, the Arctic ecosystem is experiencing a great rate of climate warming and significant changes have already been observed in recent years^1^. Hence, the structure, composition, and taxonomic diversity of microbial communities in the Arctic need to be studied urgently. In particular, fungi are an important proportion of microbial communities in Arctic habitats, such as soil^2^,^3^,^4^, mosses^5^, plant leaves^6^,^7^,^8^, and plant roots^9^,^10^,^11^,^12^. Lichens are successful colonizers of the Arctic and may play an important role in the mineral cycling patterns of Arctic habitats. However, the diversity of fungi associated with lichens in the Arctic has yet to be adequately characterized.

The lichen thalli harbor lichenicolous fungi (those that live exclusively on the lichen thalli)^13^,^14^,^15^ and endolichenic fungi (those that inhabit lichen thalli at some time during their life cycle without obligate symbiosis)^16^, in addition to the lichenized fungi (lichen mycobiont). It appears that lichen metabolism is altered by lichen-associated fungi^17^. Nutritionally, some lichen-associated fungi might utilize organics leaked from mycobiont hyphae damaged during wet/dry and freeze/thaw cycles^18^. However, details of interactions between lichen-associated fungi and lichen symbionts are still poorly understood. Arnold *et al.*^19^ found that the lichen thalli might act as ‘cradles’ of fungal diversification. A great phylogenetic diversity of lichen-associated fungi has been found in lichens from boreal, temperate, tropical and Antarctic ecosystems. Several studies were focused on culturable fungi associated with lichen thalli^15^,^19^,^20^,^21^,^22^,^23^,^24^,^25^,^26^, and a few studies have surveyed lichen-associated fungi using pyrosequencing with fungal-specific primers targeting the 18S rDNA^27^,^28^ and ITS region^29^.

The Svalbard archipelago (74 ∼ 81°N; 10 ∼ 35°E) is entirely within the High Arctic region and its vegetation is characterized by the absence of trees; this vegetation consists primarily of 742 lichen species^30^, 373 bryophyte species, and 173 vascular plant species^31^. As the most successful vegetation group in Svalbard, lichens survive several abiotic stresses (e.g., low temperature, high level of UV radiation, and long periods of darkness and light) and are important primary producers; thus, lichen-associated fungi are likely to play important roles in the Arctic terrestrial ecosystem.

To our knowledge, a great diversity of lichenicolous fungi has been found in Svalbard^14^, whereas the detailed information of endolichenic fungi in the High Arctic is still limited. For the first time, we used Roche 454 pyrosequencing to investigate the fungal communities associated with lichens in the Ny-Ålesund Region (Svalbard, High Arctic) (Fig. 1 and Fig. S1) to address the following questions: (1) what are the fungal taxa and composition associated with Arctic lichens in this region? (2) are the lichen-associated fungal taxa in the Arctic different from those in the non-Arctic regions? (3) do the richness and composition of fungal communities vary among different lichen species in the region?

## Results

### Sequence data

The raw data from 24 samples of 7 lichen species consisted of 176,063 reads, of which 68,557 reads were retained after removing sequences with different tags at each end for quality filtering and denoising (Table 1 and Table S1). After removing singletons, and OTUs assigned to the host lichen mycobionts, nonfungal organisms or had unreliable BLAST matches (max score below 200 or aligned query sequence below 200 bp), 370 fungal OTUs of 42,259 reads were included in the final matrix (Table S2). The number of OTUs in the 7 lichen species ranged from 36 to 160.

### Fungal diversity and community structure

Based on BLASTn searches in GenBank, the OTUs could be identified at different levels of precision. The taxonomic information for the 370 OTUs is presented in Table S3, and the distribution of the 370 OTUs found in the 7 lichen species is presented in Table S2. Of these OTUs, 294 belonged to Ascomycota, 54 to Basidiomycota, 2 to Zygomycota, and 20 to unknown fungi (Fig. 2). These fungal OTUs spanned 3 phyla, 14 classes, 40 orders, 51 families, 78 genera, and 55 species (Table S4). Several fungal OTUs could not be assigned to a genus or a species based on ITS sequences. Among 370 OTUs, 156 had matching sequences with similarity below 97%, and these might belong to yet undescribed fungal species that can survive in the Arctic environment. The other 214 OTUs had matching sequences with high similarity (≥97%), and the habitat information of their matching sequences was valuable.

Of these 214 fungal OTUs, most were reported previously in Arctic regions (e.g., Svalbard, North American Arctic) and areas near the Arctic (e.g., Norway, Finland, Austria, Sweden, Switzerland, Portugal, Germany, and North America). A few OTUs were closely related to fungi from alpine regions (e.g., Himalaya in the Tibetan plateau). Interestingly, 8 OTUs were even found to be closely related to fungi from Antarctica. Additionally, most of these OTUs were closely related to fungi that inhabited soils and plant tissues (e.g., wood stump, root, stem and leaf of vascular plants, and thalli of liverworts and mosses). A few OTUs were closely related to fungi from other niches, such as air dusts and streams.

Members of Ascomycota were more frequently observed than those of Basidiomycota and Zygomycota. Leotiomycetes, Dothideomycetes, and Eurotiomycetes were the major classes. The Ascomycota comprised 23 orders, with Helotiales being the most abundant and the most diverse, followed by Capnodiales and Chaetothyriales. The Basidiomycota contained 16 orders, with Sebacinales being the most abundant and Tremellales being the most diverse (Fig. 2). The distributions of OTUs among orders were different for the 7 lichen species (Table 2).

The major fungal genera detected (>200 reads) were *Atradidymella*, *Cladosporium*, *Geltingia*, *Gorgomyces*, *Hyphodiscus*, *Pichia, Polyblastia*, *Rachicladosporium*, and *Rhizoscyphus* in Asomycota, and *Cryptococcus*, and *Sebacina* in Basidiomycota, whereas the most commonly identified fungal species (>100 reads) belonged to Ascomycota, including *Geltingia associata, Polyblastia terrestis, Atradidymella muscivora, Rachicladosporium monterosium, Pichia pastoris, Rhizoscyphus ericae, Bilimbia microcarpa*, and *Elasticomyces elasticus* (Table S3).

### Dissimilarity of fungal communities among different lichen species

The Chao 1, Good’s coverage estimator and Shannon’s indices were used to evaluate and compare the diversity of the fungal communities among the 7 lichen species (Table 1). The coverage ranged from 95.80 to 99.81%, which indicated that pyrosequencing captured the dominant phylotypes. The Chao1 index (45–172) and Shannon’s Index (*H*′ = 1.63–5.20) indicated that the diversity level varied among the 7 lichen species and that the fungal communities were the most diverse in *Cetrariella delisei*, followed by *Cladonia pocillum*, *Cladonia borealis*, *Ochrolechia frigida, Flavocetraria nivalis*, *Peltigera canina*, and *Cladonia arbuscula.*

To further understand the relationships between lichen-associated fungi in the Arctic and non-Arctic regions, a phylogenetic tree was constructed using sequences of 370 fungal OTUs and fungal sequences associated with *Peltigera praetextata* in a temperate forest of the USA, which was also analyzed by 454 pyrosequencing (NCBI SRA No. SRR1183466) (Fig. 3). Only 50 fungal OTUs in the Arctic were phylogenetically close to the fungi in the temperate forest (≥97% sequence similarity). Among these 50 OTUs, 43 belonged to Ascomycota, 6 to Basidiomycota, and 1 to Zygomycota (Fig. S2). These results indicated that most of the lichen-associated fungi in this Arctic region were distinct from lichen-associated fungi in the temperate forest.

A MRPP test (*A* = 0.02698, *P* = 0.001) (Table S5) revealed a statistically significant difference among fungal communities in the 7 lichen species. Low Sorenson’s similarity coefficients (0.21–0.50) (Table 3) were found among the 7 lichen species. The highest similarity (0.50) was between *Cladonia borealis* and *Cladonia pocillum*, and the lowest similarity (0.21) was between *Ochrolechia frigida* and *Flavocetraria nivalis.* To better elucidate the distribution of OTUs among the seven lichen species, a network analysis was performed (Fig. 4). Among the 370 OTUs, 167 belonged to only one lichen species and only 3 were shared among the 7 lichen species.

## Discussion

This study reports fungal communities associated with lichens in Ny-Ålesund (Svalbard, High Arctic) by 454 pyrosequencing analyses, which help uncover the lichen-associated fungal diversity and distribution within the Arctic terrestrial ecosystem. Because lichen thalli consist primarily of mycobiont tissue, sequences of lichen mycobionts occupied an important part of pyroseuqencing data, which was also observed by Bates *et al.*^28^. The presence of sequences of other fungal taxa associated with the Arctic lichens demonstrate these lichen species harbor potential fungal propagules, although they do not represent sizable biomass portions in the lichen thalli.

Despite the extreme Arctic climate, lichen-associated fungal communities were found to be diverse in this region. Various Shannon diversity indices (*H*′ = 3.15–5.12) were obtained in the present study. It may be favorable for a host lichen thalli to form close relationships with fungi with as many different physiological attributes as possible in this extreme environment. In previous culture-based studies for lichen-associated fungi, Shannon’s indices were 1.6–3.1^21^ and 0.48–1.85^23^. The high sensitivity of 454 pyrosequencing may allow us to detect rare species and provide more detailed information on fungal diversity than the conventional methods; thus, differences in the methodology used could explain the observed differences in the diversity.

In the present study, sequences of Ascomycota were more frequently indentified than those of Basidiomycota and Chytridiomycota. U’Ren *et al.*^29^ found that sequences of Ascomycota were more frequently identified than those of Basidiomycota and unclassified phylum in lichen *Peltigera praetextata* from the temperate forest (32.4210° N, 110.7311° W; 2444 m). By contrast, sequences of Basidiomycota were more prevalent than those of Ascomycota, Zygomycota, and Glomeromycota in the plant root-associated fungal communities from Svalbard^11^. These data suggest that fungal communities associated with lichens are distinct from those associated with plant roots in Svalbard.

The major classes identified in the present study are not in accordance with the previous studies of fungi associated with non-Arctic lichens. Most of the fungal ITS sequences were Leotiomycetes, followed by Dothideomycetes, Sordariomycetes, and Eurotiomycetes in fresh lichen thalli in temperate forest of the USA^29^. In Antarctica, lichen-associated fungal 18S rDNA sequences belonged to the Arthoniomycetes, Eurotiomycetes, Lecanoromycetes, Leotiomycetes, and Sordariomycetes classes of Ascomycota, and the Tremellomycetes and Cystobasidiomycetes classes of Basidiomycota^28^. Furthermore, 320 fungal OTUs in the Arctic lichens were distinct from those in the temperate forest of the USA (as shown in Fig. S2). Taken together, the fungal communities associated with Arctic lichens are obviously different from those in the non-Arctic lichens.

Among 78 fungal genera detected in the present study, 11 were previously reported from the Arctic soils, including *Acremonium*, *Alternaria*, *Arrhenia*, *Arthrinium*, *Aspergillus*, *Cladosporium*, *Exophiala*, *Herpotrichia*, *Mortierella, Phialocephala, Oidiodendron*, and *Preussia*^2^,^3^,^4^, and 3 were observed associated with the Arctic moss, including *Cladosporium*, *Fusarium*, and *Mortierella*^5^. The fungal genera, such as *Cladosporium*, *Mortierella*, and *Alternaria*, were associated with leaves of Arctic plants^6^. In addition, 11 fungal genera were associated with plant roots in the Arctic, such as *Alatospora*, *Cadophora, Cenococcum, Cladophialophora, Cryptococcus, Fusarium, Herpotrichia, Hymenoscyphus, Mrakia, Monodictys, Mortierella, Phaeosphaeria, Rhodotorula, Russula, Sebacina*, and *Tomentella*^9^,^10^,^11^,^12^. Some species, such as *Altatospora acuminate, Cenococcum geophilum, Cladophialophora humicolae, Cryptococcus gilvescens, Cryptococcus victoriae, Monodictys arctica*, and *Rhodotorula lamellibrachiae*, were previously observed in the roots of plants (i.e., *Bistorta vivipara*, *Dryas octopetala, Saxifraga oppositifolia*, and *Salix polaris*) collected in Svalbard^7^,^8^,^9^. Members of *Rachicladosporium* are rock-inhabiting black fungi and possess peculiar physiological and physical characteristics (e.g., melanized cell walls) to successfully colonize extreme habitats^32^. Taken together, these results suggest that most of the lichen-associated fungi in this region may be from various niches in the Arctic terrestrial ecosystem.

Geographically, most fungal OTUs were closely related to fungi from the North American Arctic Transect^33^, some fungal OTUs were close to fungi from alpine regions (e.g., Himalaya in Tibet plateau), and some fungal OTUs were even found to be closely related to fungi from Antarctica. For example, one OTU was affiliated with *Elasticomyces elasticus*, which is an extremotolerant fungus isolated from Antarctic lichens^34^. Additionally, the genera *Pichia*, *Cryptococcus*, *Dioszegia*, *Malassezia*, *Mrakia*, and *Trichosporon* are extremophilic yeasts in the Arctic that also occur frequently in Antarctica^35^. The wide distribution of these fungi suggests that long-distance dispersal may be important to these fungi in the Arctic and that these fungi may serve as a good model for further ecological and evolutionary studies.

It is evident that a certain level of host-specificity for lichen-associated fungi exist in this region, although these seven lichen species have similar ecological habitats in such an abiotically stressful environment. These results are in accordance with previous studies which found that fungal communities associated with lichens were dependent on the host-lichen species^22^,^23^. These seven Arctic lichen species exhibit several differences in thallus characteristics (e.g., morphology, structure, texture) and grow on different substrates. It is possible that different fungal communities may adapt to different types of lichen thalli on different substrates. This finding is similar to the results obtained from studies on the bacteria involved in lichen symbiosis, which found that lichens were densely colonized by diverse and host-specific communities of bacteria^36^,^37^.

To our knowledge, there is little information on the functional roles of the fungi associated with lichen thalli. In the present study, some fungal OTUs were affiliated with fungal species with various functional roles. For example, *Atradidymella muscivora* is a bryophyte panthogen in the boreal and montane habitats^38^. *Rhizoscyphus ericae* is typically a symbiont of liverworts in Antarctica^39^ and could increase the amino acid uptake of root cells^40^. Because bryophytes and lichens have similar habitats and thallus morphology, these fungi may play similar roles in lichen thalli. Additionally, these lichen-associated fungi may become decomposers after lichen senescence. Some fungal genera indentified in the present study (e.g., *Acremonium* and *Coniothyrium*) were previously shown to produce extracellular enzymes, which indicates their potential role in litter degradation^18^. However, there is no direct evidence to clarify and establish connections between lichen-associated fungi and their functional roles in the present study. The ecological significance of fungi in the Arctic may be underestimated due to the limited information available in GenBank. Although there is a wealth of fungal ITS data, some ecologically important members of Arctic fungal communities have not been sequenced.

Additionally, there is no information regarding how the fungi detected in this study become associated with the lichen thalli. It may well be that some of the sequences are not true lichenicolous or endolichenic fungi and represent spore deposits rather than growing hyphae. Therefore, it is necessary to visually inspect the lichen thalli to determine whether the hyphae of some fungal taxa exist. In further studies, a combination of different technologies, such as traditional culture-based methods, *in situ* microscopy observation, metatranscriptomics, and metaproteomics, may help us answer the pending questions.

## Methods

### Study sites and sample collection

**Figure 1:**
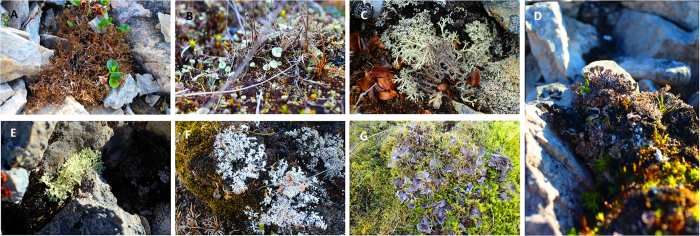
Seven lichen species studied in the Ny-Ålesund region, Svalbard (High Arctic). (**A**) *Cetrariella delisei*, (**B**) *Cladonia borealis*, (**C**) *Cladonia arbuscula*, (**D**) *Cladonia pocillum*, (**E**) *Flavocetraria nivalis*, (**F**) *Ochrolechia frigida*, (**G**) *Peltigera canina*.

**Figure 2:**
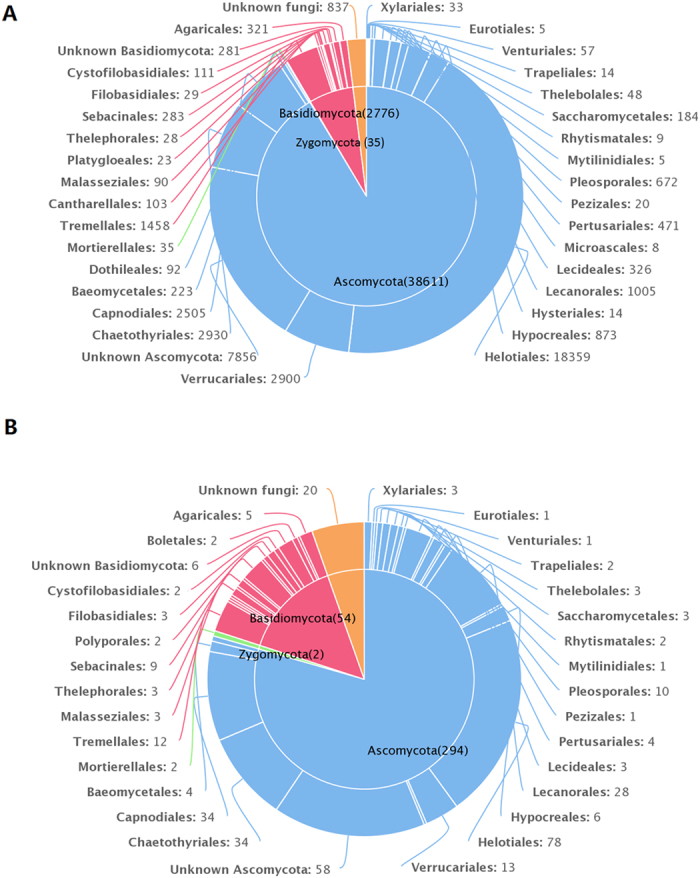
(**A**) The taxonomic distribution of 42,259 sequence reads at the phylum and order level; (**B**) the taxonomic distribution of the 370 OTUs at the phylum and order level.

**Figure 3:**
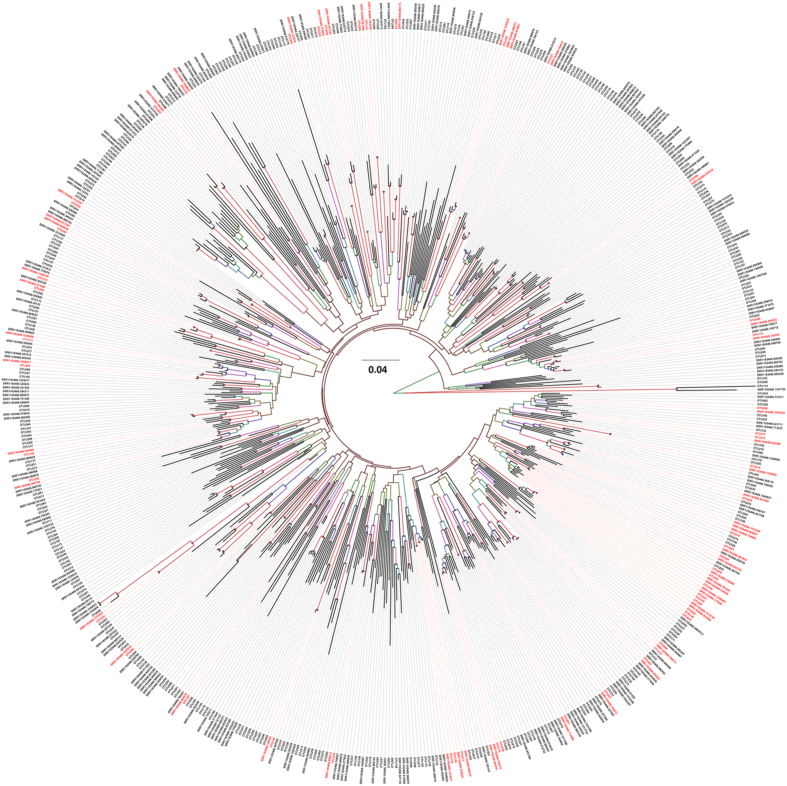
A phylogenetic tree showing the relationships between lichen-associated fungi in the Arctic (370 OTUs) and those in the non-Arctic regions. Among the 370 OTUs, 50 (in red text) have sequences with high similarity (≥97%) to those in the temperate forest (NCBI SRA No. SRR1183466). The tree was constructed with the neighbor-joining method, based on the ITS1 sequences of rDNA.

**Figure 4:**
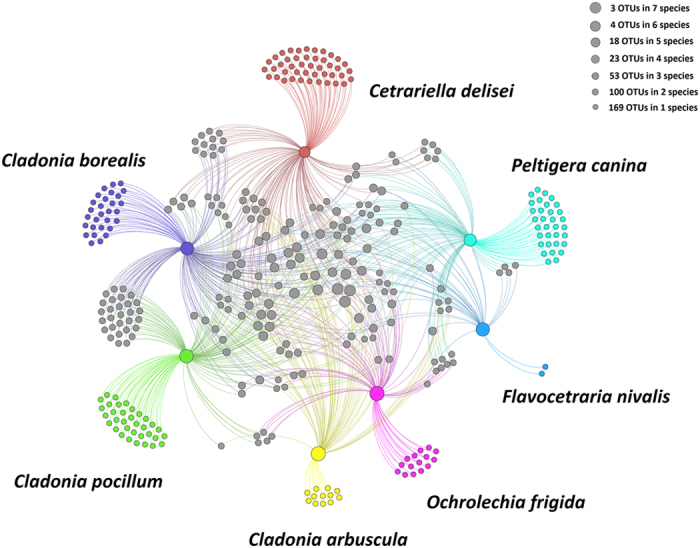
A Gephi network to visualize the 370 OTUs and demonstrate the number of shared OTUs among the 7 lichen species.

The study area is located in the Ny-Ålesund Region (78°55′ N, 11°56′ E), Spitsbergen, the largest island of the Svalbard archipelago. The Svalbard archipelago is geographically isolated from mainland Eurasia, which is entirely within the High Arctic, and has a severe climate. The Ny-Ålesund region is situated on the Brøgger peninsula (Brøggerhalvøya) and on the shore of the bay of Kongsfjorden. It has the lowest temperatures during February, at an average of −14 °C, and a high average of 5 °C during July. The sun does not set from 18 April to 24 August (midnight sun) and does not rise from 25 October to 17 February (polar night)^41^. Sampling was performed in the Ny-Ålesund region during China’s Arctic expedition in July 2013. Seven lichen species were collected in this region, including *Cetrariella delisei* (Bory ex Schaer) Kärnefelt & A. Thell, *Cladonia borealis* S. Stenroos, *Cladonia arbuscular* (Wallr) Flot., *Cladonia pocillum* (Ach). O.J. Rich., *Flavocetraria nivalis* (L.) Kärnefelt & A. Thell, *Ochrolechia frigida* (Sw). Lynge, and *Peltigera canina* (L.) Willd. (Fig. 1). The distributions of these seven lichen species are widespread and not limited to Svalbard. *C. delisei*, *C. arbuscula*, *F. nivalis*, and *O. frigida* are common in Svalbard and dominant in some places (more than 50% cover in its habitats), whereas *C. borealis*, *C. pocillum*, and *P. canina* are also common but sparse in their habitats^30^. The sample collection was under ethical approval of the Svalbard Science Forum (Research in Svalbard ID 4951 & 7126) and Chinese Arctic and Antarctic Administration (CAA), the State Oceanic Administration (SOA) of China. The detailed information of 24 lichen samples was shown in Table S1. Lichen samples were frozen at −20 °C for 10–20 days in the Yellow River Station (China) and transported to our home laboratory by flight. Samples were then stored at −80 °C within 15 days until nucleic acids were extracted.

### DNA extraction

Before DNA extraction, lichen samples were surface sterilized by immersion in 75% ethanol for 1 min, in 1% sodium hypochlorite for 2 min, and in 75% ethanol for 0.5 min. The surface sterilized tissues were then rinsed with sterile water for 0.5 min and blotted dry with sterile filter paper. We cut the sterilized samples into small segments (1 ∼ 2 cm long) using sterilized scissors and then used SuperFastPrep-1^TM^ Instrument (MP Biomedicals Co., Ltd., USA) to disrupt these segments. Genomic DNA was extracted from 72 segments of 24 lichen samples (three replicates for each sample) using a PowerSoil DNA Isolation Kit (MO BIO Laboratories, Inc., USA) according to the manufacturer’s instructions. The obtained DNA extracts were then used for the subsequent PCR and sequencing analyses.

### 454 pyrosequencing

The fungal internal transcribed spacer (ITS, ITS1–5.8S-ITS2) of nuclear ribosomal DNA sequences was amplified by using ITS1F (55′- CTTGGTCATTTAGAGGAAGTAA -3′) and ITS4 (55′- TCCTCCGCTTATTGATATGC -3′) primer sets^42^. The PCR amplification was performed using the Amplicon Fusion Primers as 55′-A-x-ITS1F-3′ and 55′-B-ITS4–3′, where A and B represent the pyrosequencing adaptors (CCATCTCATCCCTGCGTGTCTCCGACGACT and CCTATCCCCTGTGTGCCTTGGCAGTCGACT, respectively) and x represents an 8 bp-tag for sample identification (Table S6). The 20 μl reaction mixture contained the template DNA (10 ng of Template DNA), 4 μl of 5 × buffer (50 M Tris-HCl, pH 8.3–8.8), 2 μl of 2.5 nM dNTP, 0.8 μl of Fastpfu Polymerase, 2 μM of each primer and ultra pure sterilized water. The PCR amplification consisted of an initial denaturation at 95 °C for 2 min, 30 cycles of denaturation at 95 °C for 30 s, annealing at 55 °C for 30 s, and extension at 72 °C for 30 s, with a final extension at 72 °C for 5 min. After purification using the AxyPrep DNA Gel Extraction Kit (Axygen Biosciences, Inc., US) and quantification using QuantiFluor-ST (Promega Corporation, US), equimolar mixtures of multiple amplicons were used for pyrosequencing on a Roche 454 GS FLX + Titanium platform (Roche 454 Life Sciences, US) at Majorbio Bio-Pharm Technology Co., Ltd., Shanghai, China. The raw sequence reads were deposited in the NCBI sequencing read archive under Accession No. SRP045933.

### Pyrosequencing data treatment

Raw sequence data generated from pyrosequencing were processed using QIIME 1.8.0 software^43^. Briefly, the sequence libraries were split and denoised to avoid diversity overestimation caused by sequencing errors, including sequences with average quality score <20 over a 50 bp sliding window, sequences shorter than 200 bp, sequences with homopolymers longer than six nucleotides, and sequences containing ambiguous base calls or incorrect primer sequences. Operational Taxonomic Units (OTUs) were clustered with a 97% similarity cutoff using UPARSE^44^ and chimeric sequences were identified and removed using UCHIME^45^. These OTUs were then used as a basis for calculating alpha-diversity and beta-diversity metrics.

### Statistical analyses

Sequences representing the OTUs were subjected to BLASTn searches in GenBank (http://www.ncbi.nlm.nih.gov/genbank/) to determine their taxonomic affiliation. The following criteria were used to interpret the sequences from the GenBank database: for sequence identities ≥97%, the genus and species were accepted; for sequence identities between 95% and 97%, only the genus was accepted; and for sequence identities <95%, OTUs were labelled at the order, family or phylum name or as ‘unassigned’. Statistical analysis of OTU richness of each lichen sample via Chao1, Good’s coverage estimator and Shannon’s indices were performed using QIIME 1.8.0 software^43^. Sequences representing the OTUs were subjected to BLASTn searches in SRA databases (SRA Experiment set SRX481201) to determine the numbers of fungal OTUs that had the closest matching sequences (≥97% sequence similarity). A phylogenetic tree was constructed to illustrate the relationships among the fungal OTUs and their close matching sequences (≥97% sequence similarity), using MEGA v. 6.0 and the Neighbor-Joining algorithm with bootstrap values calculated from 1,000 replicate runs. Network analysis using Gephi 0.8.2 software^46^ was performed to visualize all of the OTUs and to compare their distribution among different lichen species. This network diagram allowed the visualization of the OTU partitioning among the 7 lichen species and the unveiling of unique OTUs that only belong to simple lichen species. Multiple response permutation procedure (MRPP) was performed using QIIME 1.8.0 software^43^ to test whether there were significant differences among the 7 lichen species. Sorenson’s similarity coefficient (*CS*) was employed to test the similarity of fungal composition between lichen species, which was calculated according to the formula: *CS* = *2C/(A* *+* *B)*, where *A* and *B* are the OTU numbers in samples *A* and *B*, respectively, and *C* is the number of OTU shared by the two species. *CS* is expressed with values between 0 (no similarity) and 1 (absolute similarity).

## Additional Information

**How to cite this article**: Zhang, T. *et al.* Diversity and distribution of lichen-associated fungi in the Ny-Ålesund Region (Svalbard, High Arctic) as revealed by 454 pyrosequencing. *Sci. Rep.* **5**, 14850; doi: 10.1038/srep14850 (2015).

## Supplementary Material

Supplementary Information

## Figures and Tables

**Table 1:** Information on the 7 lichen species investigated in the present study.

Lichenspecies	Lichenform	n*	Validreads	Trimmedreads	Hostmycobiontreads	Untargetedfungalreads	Unreliablereads	Singletons	Targetedreads#	TargetedOTUs	Chao1	Good’scoverageestimator	Shannon
*Cetrariella delisei*	foliose	4	43856	12338	3525	231	67	2	8513	156	167(159,188)	99.76	5.12
*Cladonia borealis*	fruticose	3	20178	8069	45	368	70	2	7584	160	172(164,196)	99.71	4.68
*Cladonia arbuscula*	fruticose	2	12714	6815	433	44	0	0	6338	78	87(80,110)	99.76	3.15
*Cladonia pocillum*	fruticose	5	26328	12718	8364	173	27	4	8450	143	153(146,175)	99.77	4.96
*Flavocetraria nivalis*	foliose	3	29253	560	46	45	13	1	455	36	45(38,74)	95.80	3.84
*Ochrolechia frigida*	crustose	4	20948	16960	11021	31	10	0	5898	77	81(78,96)	99.81	4.24
*Peltigera canina*	foliose	3	22786	6797	1569	89	116	2	5021	105	109(106,121)	99.74	3.79
Total		24	17063	68557	25003	981	303	11	42259	370			

^*^Sample numbers.

^#^Targeted reads: fungal sequences excluding sequences of host mycobionts and untargeted fungi, unreliable sequences, and singletons.

**Table 2:** Information on the taxonomic composition of fungal communities associated with the 7 lichen species.

	% ofreads	% ofOTUs	*Cetrarielladelisei*	*Cladoniaborealis*	*Cladoniaarbuscula*	*Cladoniapocillum*	*Flavocetrarianivalis*	*Ochrolechiafrigida*	*Peltigeracanina*
Ascomycota	**91.37**	**79.46**							
Dothideomycetes	**7.92**	**12.97**							
Capnodiales	5.93	9.19	2.97	2.12	16.28	6.76	1.54	2.04	7.19
Dothileales	0.22	0.54	0.28	0.34	0.58	0.05	ND	0.02	ND
Hysteriales	0.03	0.27	0.16	ND	ND	ND	ND	ND	ND
Mytilinidiales	0.01	0.27	ND	ND	ND	ND	ND	0.08	ND
Pleosporales	1.59	2.70	2.11	2.45	0.44	0.59	ND	0.12	4.40
Venturiales	0.13	0.27	ND	ND	0.90	ND	ND	ND	ND
Eurotiomycetes	**13.81**	**12.70**							
Chaetothyriales	6.93	9.19	6.33	6.59	0.28	13.29	2.20	6.23	7.25
Eurotiales	0.01	0.27	0.01	0.04	0.02	ND	ND	ND	ND
Verrucariales	6.86	3.51	1.50	3.16	0.06	2.50	ND	0.20	45.91
Leotiomycetes	**43.62**	**22.16**							
Helotiales	43.44	21.08	39.65	56.41	50.17	40.97	65.05	58.41	6.51
Pezizales	0.05	0.27	ND	ND	ND	0.24	ND	ND	ND
Rhytismatales	0.02	0.54	0.08	0.03	ND	ND	ND	ND	ND
Thelebolales	0.11	0.81	0.06	0.24	0.02	ND	ND	0.02	0.46
Lecanoromycetes	**4.83**	**11.89**							
Baeomycetales	0.53	1.08	1.97	0.57	ND	0.12	ND	ND	0.04
Lecanorales	2.38	7.57	2.08	3.68	ND	2.39	0.66	1.14	5.52
Lecideales	0.77	0.81	3.28	0.30	0.21	0.07	0.44	0.05	ND
Pertusariales	1.11	1.08	4.97	0.61	ND	0.02	ND	ND	ND
Teloschistales	0.01	0.27	ND	ND	ND	ND	ND	ND	0.04
Trapeliales	0.03	0.54	ND	0.18	ND	ND	ND	ND	ND
Saccharomycetes	**0.44**	**0.81**							
Saccharomycetales	0.44	0.81	0.19	0.37	1.37	0.07	9.89	ND	0.04
Sordariomycetes	**2.16**	**2.70**							
Hypocreales	2.07	1.62	5.87	0.04	0.02	2.33	ND	2.26	0.78
Microascales	0.02	0.27	0.01	ND	ND	ND	ND	ND	0.14
Xylariales	0.08	0.81	0.08	0.01	0.06	ND	ND	ND	0.42
Unknown Ascomycota	**18.59**	**15.68**	22.46	14.72	27.61	18.89	11.43	21.62	3.13
Basidiomycota	**6.57**	**14.59**							
Agaricomycetes	**1.81**	**6.49**							
Agaricales	0.76	1.35	0.04	0.13	ND	0.18	ND	4.94	0.04
Auriculariales	0.03	0.27	ND	ND	ND	ND	ND	ND	0.26
Boletales	0.01	0.54	0.05	ND	ND	ND	ND	ND	ND
Cantharellales	0.24	0.27	ND	ND	ND	ND	ND	1.75	ND
Polyporales	0.02	0.54	0.02	ND	ND	0.06	ND	ND	ND
Russulales	0.02	0.27	0.08	ND	ND	ND	ND	ND	ND
Sebacinales	0.67	2.43	0.35	0.88	ND	1.83	ND	0.12	0.48
Thelephorales	0.07	0.81	0.04	ND	ND	ND	ND	ND	0.50
Agaricostilbomyces	**0.01**	**0.27**							
Agaricostibales	0.01	0.27	ND	0.04	ND	ND	ND	ND	ND
Cystobasidiomycetes	0.01	0.27							
Erythrobasidiales	0.01	0.27	ND	0.05	ND	0.02	ND	ND	ND
Exobasidiomycetes	**0.21**	**0.81**							
Malasseziales	0.21	0.81	0.67	0.03	ND	0.25	ND	0.17	ND
Microbotryomycetes	0.02	0.27							
Sporidiobolales	0.02	0.27	ND	ND	0.11	0.02	ND	ND	ND
Pucciniomycetes	**0.05**	**0.27**							
Platygloeales	0.05	0.27	ND	ND	ND	ND	ND	ND	0.46
Tremellomycetes	**3.78**	**4.59**							
Cystofilobasidiales	0.26	0.54	0.01	0.96	0.49	0.01	ND	ND	0.10
Filobasidiales	0.07	0.81	0.07	0.04	ND	ND	2.64	ND	0.16
Tremellales	3.45	3.24	3.09	4.01	0.28	0.65	4.62	0.44	15.36
Unknown Basidiomycota	**0.66**	**1.62**	0.01	0.38	0.66	2.09	ND	0.19	0.42
Zygomycota	**0.08**	**0.54**							
Zygomycetes	**0.08**	**0.54**							
Mortierellales	0.08	0.54	ND	0.41	ND	0.05	ND	ND	ND
Unknown fungi	1.98	5.41	1.48	1.20	0.43	6.54	1.54	0.20	0.42

ND: not detected. The second and third columns represent the percentage of total reads and total number of OTUs across the host species. The last 7 columns provide a taxonomic overview of the fungal communities found in each of the 7 lichen species, which are represented as the percentage of reads.

**Table 3:** Sorenson’s similarity coefficients for fungal OTUs among the 7 lichen species in the Ny-Ålesund Region.

Lichen species	*Cetrarielladelisei*	*Cladoniaborealis*	*Cladoniaarbuscula*	*Cladoniapocillum*	*Flavocetrarianivalis*	*Ochrolechiafrigida*	*Peltigeracanina*
*Cetrariella delisei*							
*Cladonia borealis*	0.44	—					
*Cladonia arbuscula*	0.37	0.43	—				
*Cladonia pocillum*	0.39	0.50	0.29	—			
*Flavocetraria nivalis*	0.30	0.33	0.25	0.21	—		
*Ochrolechia frigida*	0.29	0.25	0.26	0.29	0.21	—	
*Peltigera canina*	0.30	0.25	0.24	0.22	0.26	0.25	—
