# Supplementary material for: Diversity and distribution of lichen-associated fungi in the Ny-Ålesund Region (Svalbard, High Arctic) as revealed by 454 pyrosequencing
Source: Sci Rep. 2015 Oct 14;5:14850. doi: 10.1038/srep14850 (PMC4604449; doi:10.1038/srep14850)
Supplement: Supplementary Information [file srep14850-s1.docx]

**Supplementary Material**

**Diversity and distribution of lichen-associated fungi in the Ny-Ålesund Region (Svalbard, High Arctic) as revealed by 454 pyrosequencing**

Tao Zhang^1^, Xin-Li Wei^2^, Yu-Qin Zhang^1^, Hong-Yu Liu^1^, & Li-Yan Yu^1*^

1. China Pharmaceutical Culture Collection, Institute of Medicinal Biotechnology, Chinese Academy of Medical Sciences & Peking Union Medical College, Beijing 100050, PR China

2. State Key Laboratory of Mycology, Institute of Microbiology, Chinese Academy of Sciences, Beijing 100101, PR China

* Corresponding Author E-mail: yly@cpcc.ac.cn; Tel/fax:+86 10 63187118


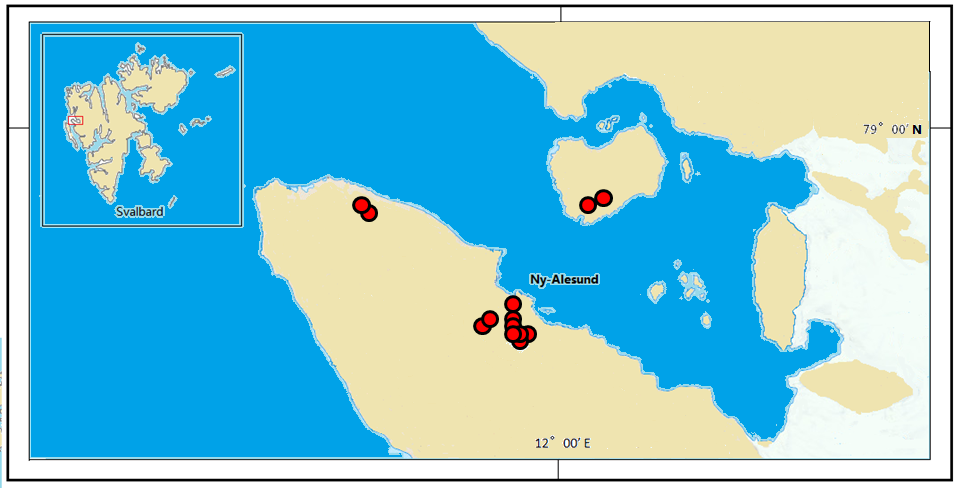


**Figure S1 |** The map of the lichen sampling sites in the Ny-Ålesund Region, Svalbard (created using drawing tool software in Window 8.0).

**
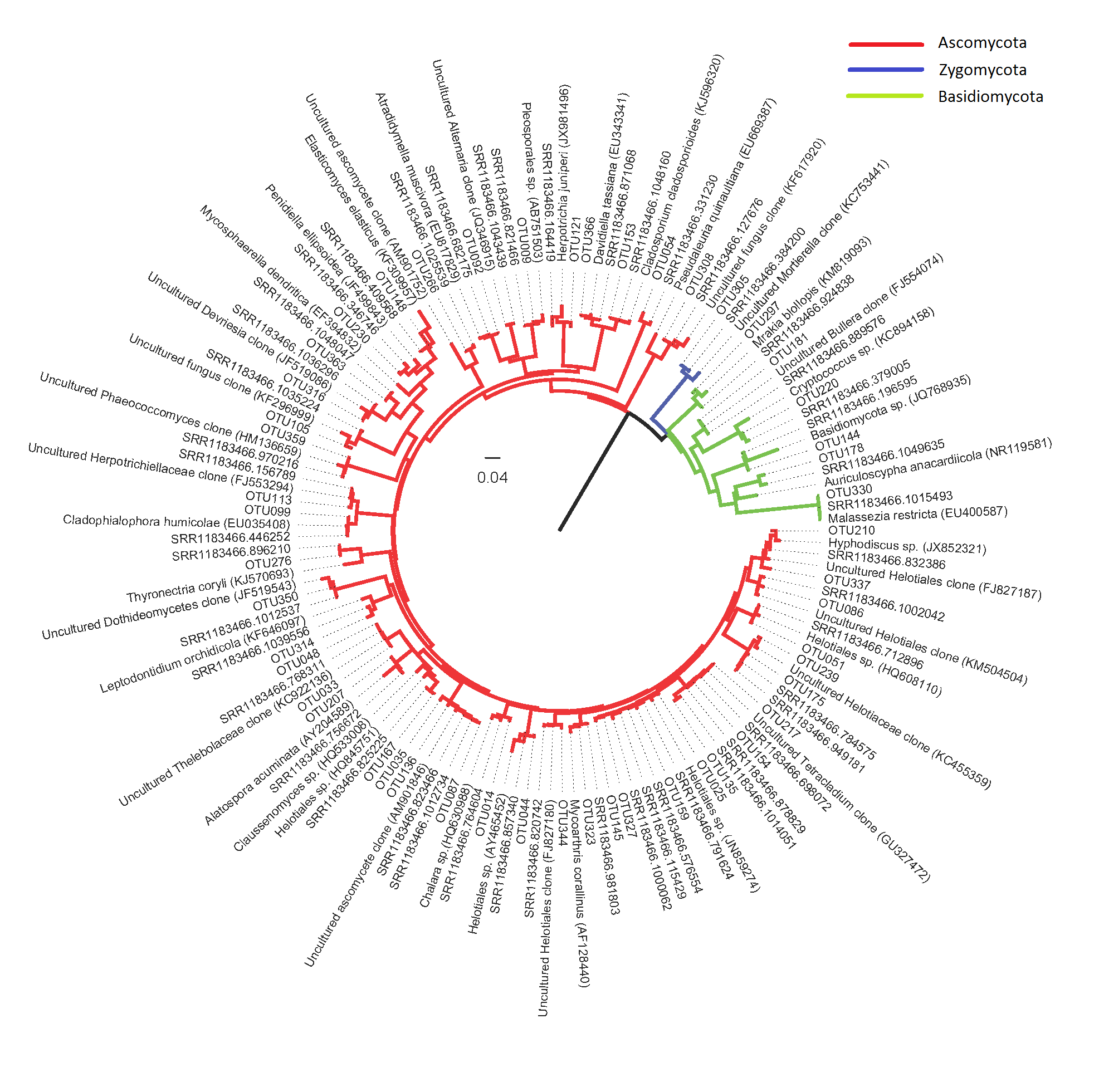
**

**Figure S2 |** A phylogenetic tree showing the relationships between the lichen-associated fungi in the Arctic and those in the non-Arctic regions. These 50 OTUs have sequences with high similarity (≥97%) to those in the temperate forest (NCBI SRA No. SRR1183466). The tree was constructed using the neighbor-joining method, based on the ITS1 sequences of rDNA.

**Table S1 |** Information on the 24 lichen samples and their respective pyrosequencing data in the Ny-Ålesund region, Svalbard.

| **Sample code** | **Lichen species** | **Coordination** | **Latitude** | **Habitat** | **Valid reads** | **Trimmed reads** | **Host mycobiont reads** | **Untargeted fungal reads** | **Unreliable reads** | **Singletons** | **Targeted reads** | **OTUs** |
| --- | --- | --- | --- | --- | --- | --- | --- | --- | --- | --- | --- | --- |
| ZT2013175 | *Cetrariella delisei* | 78°58’11.88’’N; 12°04’00.75’’E | 87m | Among rocks | 2884 | 2496 | 2315 | 25 | 6 | 0 | 150 | 17 |
| ZT2013030 | *Cetrariella delisei* | 78°54’42.44’’N; 11°58’30.16’’E | 38m | Among rocks | 4825 | 3080 | 1131 | 17 | 0 | 0 | 1932 | 45 |
| ZT2013096 | *Cetrariella delisei* | 78°57’53.41’’N; 11°35’05.03’’E | 18m | Among mosses | 17516 | 3107 | 55 | 187 | 0 | 1 | 2864 | 74 |
| ZT2013234 | *Cetrariella delisei* | 78°54’40.36’’N; 11°56’59.24’’E | 74m | Among mosses | 18631 | 3655 | 24 | 2 | 61 | 1 | 3567 | 70 |
| ZT2013128 | *Cladonia borealis* | 78°54’48.54’’N; 11°57’09.60’’E | 44m | On soil | 7190 | 2499 | 7 | 87 | 44 | 0 | 2361 | 93 |
| ZT2013230 | *Cladonia borealis* | 78°55’01.55’’N; 11°56’47.22’’E | 23m | On soil | 6834 | 2538 | 38 | 34 | 0 | 0 | 2466 | 50 |
| ZT2013076 | *Cladonia borealis* | 78°54’53.43’’N; 11°51’29.11’’E | 74m | On soil | 6154 | 3032 | 0 | 247 | 26 | 2 | 2757 | 81 |
| ZT2013129 | *Cladonia arbuscula* | 78°54’44.92’’N; 11°57’08.42’’E | 55m | Among mosses | 6614 | 3018 | 309 | 37 | 0 | 0 | 2672 | 62 |
| ZT2013212 | *Cladonia arbuscula* | 78°54’16,71’’N; 12°00’59.49’’E | 66m | Among plants | 6100 | 3797 | 124 | 7 | 0 | 0 | 3666 | 33 |
| ZT2013155 | *Cladonia pocillum* | 78°57’58.92’’N; 12°03’35.50’’E | 46m | On soil | 5579 | 4496 | 4372 | 4 | 0 | 0 | 120 | 17 |
| ZT2013231 | *Cladonia pocillum* | 78°55’00.94’’N; 11°56’48.50’’E | 24m | On soil | 5188 | 4388 | 3775 | 119 | 0 | 1 | 493 | 38 |
| ZT2013211 | *Cladonia pocillum* | 78°54’19.40’’N; 12°00’33.34’’E | 64m | On soil | 3508 | 2437 | 55 | 1 | 1 | 1 | 2379 | 42 |
| ZT2013125 | *Cladonia pocillum* | 78°54’46.96’’N; 11°57’13.50’’E | 47m | On soil | 5155 | 3266 | 161 | 48 | 26 | 1 | 3030 | 65 |
| ZT2013209 | *Cladonia pocillum* | 78°57’37.34’’N; 11°36’17.67’’E | 33m | On soil | 6898 | 2431 | 1 | 1 | 0 | 1 | 2428 | 44 |
| ZT2013204 | *Flavocetraria nivalis* | 78°57’37.30’’N; 11°36’26.58’’E | 32m | Among mosses | 10141 | 219 | 16 | 2 | 0 | 0 | 201 | 18 |
| ZT2013246 | *Flavocetraria nivalis* | 78°54’59.42’’N; 11°51’29.47’’E | 66m | Among plants | 10446 | 225 | 15 | 43 | 13 | 0 | 154 | 17 |
| ZT2013235 | *Flavocetraria nivalis* | 78°54’40.29’’N; 11°56’58.89’’E | 75m | Among rocks | 8666 | 116 | 15 | 0 | 0 | 1 | 100 | 14 |
| ZT2013079 | *Ochrolechia frigida* | 78°54’53.84’’N; 11°51’34.44’’E | 75m | On rock | 5621 | 4823 | 4664 | 2 | 3 | 0 | 154 | 10 |
| ZT2013083 | *Ochrolechia frigida* | 78°54’50.99’’N; 11°51’18.16’’E | 83m | Among mosses | 4580 | 3291 | 725 | 1 | 0 | 0 | 2565 | 17 |
| ZT2013205 | *Ochrolechia frigida* | 78°57’35.00’’N; 11°36’36.67’’E | 35m | Among mosses | 5647 | 4471 | 2423 | 19 | 7 | 0 | 2022 | 39 |
| ZT2013209o | *Ochrolechia frigida* | 78°54’52.11’’N; 11°35’34.16’’E | 19m | Among mosses | 5100 | 4375 | 3209 | 9 | 0 | 0 | 1157 | 43 |
| ZT2013054 | *Peltigera canina* | 78°55’18.64’’N; 11°56’01.43’’E | 17m | On soil | 5189 | 3881 | 0 | 89 | 110 | 1 | 3681 | 78 |
| ZT2013104 | *Peltigera canina* | 78°57’38.31’’N; 11°35’56.97’’E | 34m | Among mosses | 9532 | 1188 | 56 | 0 | 0 | 1 | 1131 | 29 |
| ZT2013198 | *Peltigera canina* | 78°57’36.93’’N; 11°36’02.83’’E | 35m | Among mosses | 8065 | 1728 | 1513 | 0 | 6 | 0 | 209 | 19 |

**Table S2 |** Distribution of the 370 OTUs found in the 7 lichen species, including their number of sequences.

| OUT ID | *Cetrariella delisei* | *Cladonia borealis* | *Cladonia arbuscula* | *Cladonia pocillum* | *Flavocetraria nivalis* | *Ochrolechia frigida* | *Peltigera canina* |
| --- | --- | --- | --- | --- | --- | --- | --- |
| OTU1 | 26 | 0 | 74 | 0 | 0 | 0 | 0 |
| OTU2 | 85 | 8 | 848 | 25 | 0 | 144 | 2 |
| OTU3 | 0 | 1 | 0 | 98 | 0 | 0 | 0 |
| OTU4 | 0 | 17 | 0 | 0 | 0 | 0 | 0 |
| OTU5 | 0 | 0 | 12 | 0 | 0 | 0 | 0 |
| OTU6 | 0 | 0 | 0 | 0 | 2 | 0 | 16 |
| OTU7 | 7 | 72 | 14 | 7 | 0 | 0 | 19 |
| OTU8 | 0 | 0 | 0 | 0 | 0 | 0 | 2 |
| OTU9 | 13 | 13 | 3 | 3 | 0 | 0 | 7 |
| OTU10 | 0 | 8 | 0 | 9 | 0 | 0 | 0 |
| OTU11 | 0 | 0 | 772 | 0 | 0 | 0 | 1 |
| OTU12 | 0 | 22 | 2 | 6 | 0 | 0 | 0 |
| OTU13 | 0 | 0 | 4 | 0 | 0 | 0 | 0 |
| OTU14 | 0 | 0 | 0 | 0 | 0 | 0 | 21 |
| OTU15 | 0 | 0 | 0 | 432 | 0 | 0 | 0 |
| OTU16 | 0 | 0 | 0 | 0 | 0 | 0 | 3 |
| OTU17 | 67 | 0 | 0 | 0 | 0 | 0 | 0 |
| OTU18 | 0 | 9 | 0 | 0 | 0 | 0 | 0 |
| OTU19 | 8 | 22 | 36 | 4 | 0 | 1 | 0 |
| OTU20 | 0 | 0 | 227 | 0 | 0 | 0 | 0 |
| OTU21 | 0 | 33 | 0 | 12 | 0 | 0 | 0 |
| OTU22 | 0 | 0 | 0 | 14 | 0 | 0 | 0 |
| OTU23 | 0 | 1 | 0 | 146 | 0 | 0 | 0 |
| OTU24 | 0 | 0 | 0 | 2 | 0 | 0 | 0 |
| OTU25 | 3 | 0 | 0 | 0 | 0 | 11 | 0 |
| OTU26 | 0 | 4 | 0 | 0 | 0 | 0 | 0 |
| OTU27 | 25 | 235 | 3 | 64 | 0 | 0 | 0 |
| OTU28 | 12 | 85 | 3 | 30 | 0 | 0 | 2 |
| OTU29 | 10 | 0 | 0 | 0 | 0 | 0 | 0 |
| OTU30 | 9 | 0 | 0 | 0 | 0 | 0 | 0 |
| OTU31 | 9 | 0 | 0 | 0 | 0 | 0 | 0 |
| OTU32 | 0 | 0 | 0 | 0 | 3 | 0 | 156 |
| OTU33 | 3 | 14 | 1 | 0 | 0 | 1 | 2 |
| OTU34 | 0 | 0 | 9 | 0 | 0 | 0 | 0 |
| OTU35 | 5 | 14 | 2 | 9 | 1 | 556 | 0 |
| OTU36 | 0 | 0 | 0 | 7 | 0 | 0 | 0 |
| OTU37 | 0 | 11 | 0 | 0 | 0 | 0 | 1 |
| OTU38 | 0 | 7 | 0 | 0 | 0 | 0 | 19 |
| OTU39 | 274 | 7 | 13 | 6 | 2 | 0 | 0 |
| OTU40 | 5 | 0 | 0 | 0 | 0 | 0 | 0 |
| OTU41 | 2 | 4 | 1 | 0 | 12 | 0 | 576 |
| OTU42 | 0 | 517 | 0 | 105 | 0 | 0 | 0 |
| OTU43 | 1 | 0 | 0 | 9 | 0 | 0 | 0 |
| OTU44 | 0 | 0 | 0 | 0 | 79 | 0 | 0 |
| OTU45 | 109 | 0 | 0 | 0 | 0 | 0 | 0 |
| OTU46 | 3 | 0 | 0 | 0 | 0 | 0 | 0 |
| OTU47 | 0 | 6 | 0 | 0 | 0 | 0 | 0 |
| OTU48 | 2 | 4 | 0 | 0 | 0 | 0 | 0 |
| OTU49 | 0 | 0 | 0 | 0 | 0 | 4 | 178 |
| OTU50 | 0 | 0 | 0 | 0 | 0 | 0 | 18 |
| OTU51 | 278 | 135 | 0 | 80 | 0 | 0 | 0 |
| OTU52 | 19 | 11 | 0 | 556 | 0 | 339 | 0 |
| OTU53 | 0 | 2 | 0 | 2 | 0 | 0 | 0 |
| OTU54 | 0 | 2 | 0 | 352 | 0 | 0 | 0 |
| OTU55 | 7 | 2 | 0 | 0 | 0 | 0 | 0 |
| OTU56 | 4 | 0 | 0 | 0 | 0 | 0 | 0 |
| OTU57 | 0 | 3 | 3 | 320 | 0 | 0 | 0 |
| OTU58 | 10 | 0 | 0 | 0 | 0 | 0 | 0 |
| OTU59 | 0 | 4 | 0 | 2 | 0 | 0 | 0 |
| OTU60 | 0 | 0 | 0 | 0 | 0 | 213 | 0 |
| OTU61 | 0 | 5 | 0 | 0 | 0 | 0 | 0 |
| OTU62 | 3 | 0 | 0 | 0 | 0 | 3 | 0 |
| OTU63 | 0 | 3 | 0 | 0 | 2 | 0 | 2 |
| OTU64 | 82 | 0 | 0 | 0 | 0 | 0 | 0 |
| OTU65 | 14 | 1 | 2 | 162 | 0 | 27 | 0 |
| OTU66 | 0 | 0 | 0 | 0 | 0 | 1 | 14 |
| OTU67 | 0 | 0 | 0 | 0 | 0 | 0 | 2 |
| OTU68 | 0 | 2 | 0 | 256 | 0 | 0 | 0 |
| OTU69 | 0 | 0 | 0 | 4 | 0 | 0 | 0 |
| OTU70 | 0 | 0 | 0 | 7 | 0 | 0 | 0 |
| OTU71 | 2 | 1 | 0 | 0 | 0 | 0 | 0 |
| OTU72 | 0 | 10 | 0 | 1 | 0 | 78 | 0 |
| OTU73 | 11 | 0 | 0 | 0 | 0 | 5 | 0 |
| OTU74 | 1 | 31 | 168 | 55 | 0 | 0 | 0 |
| OTU75 | 0 | 15 | 0 | 0 | 0 | 0 | 21 |
| OTU76 | 6 | 0 | 0 | 0 | 0 | 18 | 0 |
| OTU77 | 0 | 23 | 0 | 0 | 0 | 0 | 0 |
| OTU78 | 0 | 0 | 3 | 0 | 0 | 0 | 0 |
| OTU79 | 941 | 0 | 0 | 0 | 0 | 0 | 0 |
| OTU80 | 39 | 16 | 0 | 0 | 4 | 0 | 4 |
| OTU81 | 4 | 112 | 0 | 8 | 0 | 0 | 0 |
| OTU82 | 3 | 0 | 2 | 0 | 0 | 0 | 0 |
| OTU83 | 7 | 5 | 0 | 1 | 0 | 0 | 0 |
| OTU84 | 0 | 0 | 0 | 3 | 0 | 0 | 0 |
| OTU85 | 0 | 0 | 0 | 0 | 0 | 0 | 4 |
| OTU86 | 0 | 0 | 6 | 0 | 0 | 0 | 0 |
| OTU87 | 75 | 48 | 5 | 1769 | 12 | 509 | 4 |
| OTU88 | 0 | 26 | 0 | 0 | 0 | 0 | 0 |
| OTU89 | 0 | 0 | 0 | 0 | 0 | 103 | 0 |
| OTU90 | 0 | 0 | 0 | 0 | 0 | 11 | 0 |
| OTU91 | 0 | 0 | 0 | 5 | 0 | 0 | 0 |
| OTU92 | 0 | 1 | 1 | 0 | 0 | 4 | 186 |
| OTU93 | 4 | 0 | 0 | 0 | 0 | 0 | 0 |
| OTU94 | 140 | 0 | 0 | 0 | 0 | 0 | 0 |
| OTU95 | 0 | 0 | 0 | 0 | 0 | 0 | 10 |
| OTU96 | 0 | 32 | 0 | 0 | 0 | 0 | 0 |
| OTU97 | 0 | 14 | 0 | 0 | 0 | 0 | 0 |
| OTU98 | 0 | 0 | 0 | 0 | 0 | 8 | 0 |
| OTU99 | 0 | 0 | 0 | 5 | 0 | 0 | 0 |
| OTU100 | 0 | 4 | 0 | 0 | 0 | 0 | 0 |
| OTU101 | 96 | 0 | 2 | 0 | 1 | 0 | 0 |
| OTU102 | 0 | 0 | 0 | 4 | 0 | 0 | 0 |
| OTU103 | 0 | 1 | 2 | 0 | 4 | 0 | 0 |
| OTU104 | 0 | 3 | 0 | 0 | 0 | 0 | 0 |
| OTU105 | 0 | 12 | 0 | 4 | 0 | 53 | 0 |
| OTU106 | 0 | 0 | 0 | 0 | 0 | 133 | 0 |
| OTU107 | 0 | 14 | 0 | 119 | 0 | 0 | 0 |
| OTU108 | 14 | 3 | 0 | 46 | 29 | 0 | 0 |
| OTU109 | 0 | 0 | 57 | 0 | 0 | 0 | 0 |
| OTU110 | 2 | 0 | 1 | 36 | 0 | 0 | 0 |
| OTU111 | 0 | 3 | 1 | 0 | 0 | 0 | 0 |
| OTU112 | 17 | 0 | 0 | 57 | 3 | 0 | 0 |
| OTU113 | 0 | 0 | 0 | 32 | 0 | 0 | 0 |
| OTU114 | 71 | 0 | 0 | 0 | 0 | 0 | 0 |
| OTU115 | 0 | 3 | 0 | 0 | 0 | 0 | 0 |
| OTU116 | 0 | 0 | 0 | 183 | 0 | 0 | 0 |
| OTU117 | 0 | 73 | 0 | 1 | 0 | 0 | 0 |
| OTU118 | 4 | 0 | 0 | 0 | 0 | 0 | 0 |
| OTU119 | 52 | 15 | 0 | 36 | 0 | 2 | 7 |
| OTU120 | 3 | 0 | 0 | 1 | 0 | 0 | 11 |
| OTU121 | 0 | 1 | 3 | 0 | 0 | 0 | 1 |
| OTU122 | 0 | 0 | 0 | 0 | 0 | 0 | 135 |
| OTU123 | 0 | 0 | 0 | 0 | 0 | 2 | 0 |
| OTU124 | 0 | 83 | 0 | 20 | 0 | 0 | 0 |
| OTU125 | 1 | 24 | 16 | 0 | 0 | 0 | 0 |
| OTU126 | 3 | 0 | 0 | 0 | 0 | 0 | 0 |
| OTU127 | 5 | 1 | 0 | 0 | 0 | 0 | 0 |
| OTU128 | 0 | 11 | 0 | 0 | 0 | 2 | 0 |
| OTU129 | 0 | 0 | 0 | 0 | 1 | 0 | 7 |
| OTU130 | 12 | 0 | 0 | 0 | 0 | 0 | 0 |
| OTU131 | 131 | 6 | 4 | 1 | 0 | 0 | 0 |
| OTU132 | 123 | 0 | 0 | 0 | 0 | 0 | 19 |
| OTU133 | 0 | 0 | 0 | 7 | 0 | 0 | 0 |
| OTU134 | 15 | 2 | 0 | 0 | 0 | 0 | 0 |
| OTU135 | 0 | 0 | 3 | 0 | 3 | 3 | 6 |
| OTU136 | 15 | 4 | 4 | 17 | 0 | 494 | 0 |
| OTU137 | 0 | 0 | 7 | 2 | 0 | 0 | 0 |
| OTU138 | 0 | 0 | 4 | 0 | 0 | 0 | 16 |
| OTU139 | 0 | 5 | 0 | 249 | 0 | 0 | 0 |
| OTU140 | 133 | 1 | 0 | 0 | 0 | 0 | 0 |
| OTU141 | 0 | 0 | 0 | 6 | 0 | 0 | 0 |
| OTU142 | 0 | 0 | 0 | 0 | 0 | 17 | 5 |
| OTU143 | 14 | 0 | 0 | 0 | 0 | 0 | 0 |
| OTU144 | 0 | 2 | 2 | 0 | 0 | 0 | 0 |
| OTU145 | 2 | 0 | 1 | 0 | 92 | 8 | 0 |
| OTU146 | 32 | 37 | 15 | 0 | 0 | 0 | 0 |
| OTU147 | 1 | 0 | 0 | 0 | 0 | 40 | 0 |
| OTU148 | 2 | 2 | 0 | 0 | 0 | 0 | 5 |
| OTU149 | 0 | 51 | 0 | 1 | 0 | 0 | 0 |
| OTU150 | 11 | 0 | 0 | 0 | 0 | 0 | 0 |
| OTU151 | 1 | 0 | 0 | 0 | 0 | 0 | 13 |
| OTU152 | 0 | 29 | 0 | 0 | 0 | 0 | 0 |
| OTU153 | 7 | 1 | 0 | 2 | 0 | 0 | 1 |
| OTU154 | 13 | 1 | 0 | 6 | 1 | 0 | 0 |
| OTU155 | 0 | 12 | 0 | 0 | 0 | 0 | 0 |
| OTU156 | 1 | 6 | 40 | 177 | 0 | 2 | 0 |
| OTU157 | 5 | 4 | 12 | 2 | 0 | 0 | 0 |
| OTU158 | 0 | 0 | 0 | 2 | 0 | 0 | 0 |
| OTU159 | 34 | 0 | 0 | 0 | 0 | 0 | 17 |
| OTU160 | 1 | 0 | 0 | 0 | 0 | 0 | 7 |
| OTU161 | 7 | 8 | 0 | 6 | 0 | 0 | 1 |
| OTU162 | 3 | 0 | 0 | 0 | 0 | 0 | 0 |
| OTU163 | 4 | 2 | 0 | 0 | 0 | 0 | 0 |
| OTU164 | 3 | 0 | 0 | 0 | 1 | 0 | 0 |
| OTU165 | 0 | 0 | 0 | 0 | 0 | 0 | 5 |
| OTU166 | 0 | 40 | 0 | 0 | 0 | 0 | 0 |
| OTU167 | 0 | 113 | 0 | 0 | 0 | 0 | 0 |
| OTU168 | 279 | 649 | 33 | 227 | 7 | 77 | 4 |
| OTU169 | 0 | 2 | 0 | 0 | 0 | 0 | 1 |
| OTU170 | 318 | 1 | 0 | 0 | 0 | 0 | 0 |
| OTU171 | 0 | 0 | 0 | 21 | 0 | 0 | 0 |
| OTU172 | 93 | 0 | 0 | 3 | 0 | 0 | 0 |
| OTU173 | 0 | 33 | 0 | 1 | 0 | 0 | 0 |
| OTU174 | 1 | 7 | 10 | 1 | 0 | 25 | 0 |
| OTU175 | 2 | 5 | 12 | 0 | 0 | 0 | 22 |
| OTU176 | 1 | 0 | 0 | 2 | 0 | 1 | 10 |
| OTU177 | 0 | 0 | 16 | 0 | 0 | 0 | 0 |
| OTU178 | 0 | 0 | 0 | 0 | 0 | 0 | 23 |
| OTU179 | 0 | 0 | 0 | 134 | 0 | 0 | 0 |
| OTU180 | 0 | 0 | 0 | 0 | 0 | 3 | 0 |
| OTU181 | 0 | 56 | 0 | 0 | 1 | 0 | 7 |
| OTU182 | 3 | 0 | 0 | 0 | 0 | 0 | 0 |
| OTU183 | 0 | 0 | 0 | 0 | 0 | 0 | 10 |
| OTU184 | 0 | 0 | 0 | 78 | 0 | 0 | 0 |
| OTU185 | 0 | 20 | 1 | 1 | 0 | 0 | 0 |
| OTU186 | 13 | 0 | 0 | 18 | 0 | 0 | 1 |
| OTU187 | 1 | 7 | 0 | 31 | 2 | 0 | 52 |
| OTU188 | 0 | 0 | 2657 | 0 | 0 | 0 | 0 |
| OTU189 | 3 | 0 | 0 | 18 | 0 | 45 | 0 |
| OTU190 | 0 | 0 | 0 | 7 | 0 | 6 | 0 |
| OTU191 | 0 | 0 | 0 | 0 | 0 | 0 | 11 |
| OTU192 | 0 | 7 | 0 | 0 | 0 | 0 | 0 |
| OTU193 | 0 | 7 | 0 | 0 | 0 | 5 | 0 |
| OTU194 | 0 | 0 | 0 | 0 | 0 | 8 | 0 |
| OTU195 | 5 | 0 | 1 | 0 | 0 | 0 | 0 |
| OTU196 | 1 | 0 | 0 | 17 | 0 | 358 | 4 |
| OTU197 | 0 | 0 | 0 | 5 | 0 | 1 | 0 |
| OTU198 | 0 | 0 | 0 | 12 | 0 | 0 | 0 |
| OTU199 | 0 | 0 | 0 | 0 | 0 | 6 | 0 |
| OTU200 | 0 | 3 | 0 | 5 | 0 | 0 | 0 |
| OTU201 | 4 | 0 | 0 | 18 | 0 | 0 | 0 |
| OTU202 | 15 | 3 | 1 | 0 | 0 | 0 | 0 |
| OTU203 | 0 | 2 | 0 | 0 | 0 | 0 | 0 |
| OTU204 | 0 | 0 | 7 | 0 | 0 | 0 | 0 |
| OTU205 | 0 | 0 | 0 | 0 | 0 | 0 | 99 |
| OTU206 | 0 | 0 | 0 | 0 | 0 | 0 | 6 |
| OTU207 | 1 | 0 | 0 | 0 | 0 | 0 | 11 |
| OTU208 | 24 | 0 | 0 | 0 | 0 | 0 | 0 |
| OTU209 | 0 | 0 | 0 | 0 | 0 | 0 | 34 |
| OTU210 | 162 | 12 | 9 | 0 | 55 | 925 | 0 |
| OTU211 | 17 | 21 | 0 | 0 | 0 | 0 | 0 |
| OTU212 | 12 | 0 | 0 | 2 | 0 | 0 | 0 |
| OTU213 | 0 | 0 | 0 | 0 | 0 | 8 | 0 |
| OTU214 | 0 | 0 | 0 | 3 | 0 | 0 | 0 |
| OTU215 | 0 | 0 | 0 | 0 | 0 | 0 | 2 |
| OTU216 | 1176 | 0 | 0 | 0 | 0 | 0 | 0 |
| OTU217 | 3 | 0 | 0 | 0 | 13 | 0 | 0 |
| OTU218 | 12 | 0 | 0 | 0 | 0 | 0 | 0 |
| OTU219 | 0 | 6 | 0 | 1 | 0 | 0 | 0 |
| OTU220 | 4 | 0 | 0 | 0 | 0 | 0 | 0 |
| OTU221 | 0 | 0 | 4 | 0 | 0 | 0 | 0 |
| OTU222 | 0 | 1018 | 0 | 38 | 0 | 0 | 0 |
| OTU223 | 3 | 0 | 0 | 0 | 0 | 0 | 0 |
| OTU224 | 13 | 0 | 0 | 0 | 0 | 0 | 1 |
| OTU225 | 168 | 2 | 0 | 0 | 0 | 0 | 0 |
| OTU226 | 2 | 0 | 0 | 0 | 0 | 0 | 0 |
| OTU227 | 0 | 0 | 0 | 3 | 0 | 0 | 0 |
| OTU228 | 0 | 1 | 8 | 0 | 0 | 0 | 0 |
| OTU229 | 0 | 0 | 0 | 15 | 0 | 0 | 0 |
| OTU230 | 6 | 5 | 0 | 0 | 0 | 2 | 2 |
| OTU231 | 0 | 0 | 0 | 0 | 0 | 0 | 4 |
| OTU232 | 0 | 1 | 0 | 78 | 0 | 0 | 0 |
| OTU233 | 0 | 4 | 0 | 0 | 0 | 0 | 0 |
| OTU234 | 2 | 0 | 0 | 5 | 0 | 0 | 1 |
| OTU235 | 2 | 0 | 0 | 0 | 0 | 0 | 0 |
| OTU236 | 0 | 0 | 0 | 0 | 0 | 0 | 17 |
| OTU237 | 0 | 0 | 0 | 2 | 0 | 0 | 0 |
| OTU238 | 0 | 31 | 0 | 2 | 0 | 0 | 0 |
| OTU239 | 2 | 50 | 1 | 2 | 0 | 0 | 17 |
| OTU240 | 0 | 0 | 0 | 0 | 0 | 1 | 17 |
| OTU241 | 1 | 16 | 0 | 0 | 0 | 0 | 1 |
| OTU242 | 0 | 7 | 0 | 0 | 0 | 0 | 0 |
| OTU243 | 0 | 0 | 0 | 0 | 0 | 7 | 0 |
| OTU244 | 269 | 2 | 12 | 0 | 0 | 0 | 0 |
| OTU245 | 0 | 0 | 0 | 0 | 0 | 0 | 2 |
| OTU246 | 16 | 4 | 1 | 0 | 0 | 0 | 0 |
| OTU247 | 0 | 0 | 0 | 9 | 0 | 0 | 0 |
| OTU248 | 1 | 0 | 0 | 13 | 0 | 0 | 0 |
| OTU249 | 0 | 1 | 0 | 0 | 6 | 229 | 0 |
| OTU250 | 12 | 2 | 0 | 12 | 0 | 2 | 0 |
| OTU251 | 0 | 0 | 0 | 0 | 0 | 0 | 2 |
| OTU252 | 0 | 1795 | 0 | 0 | 0 | 0 | 0 |
| OTU253 | 0 | 0 | 0 | 3 | 0 | 2 | 1 |
| OTU254 | 0 | 15 | 0 | 0 | 0 | 3 | 0 |
| OTU255 | 1 | 3 | 1 | 0 | 0 | 0 | 0 |
| OTU256 | 334 | 0 | 0 | 0 | 0 | 0 | 0 |
| OTU257 | 1 | 0 | 0 | 9 | 0 | 0 | 0 |
| OTU258 | 638 | 146 | 9 | 41 | 1 | 13 | 1 |
| OTU259 | 0 | 28 | 39 | 24 | 0 | 22 | 0 |
| OTU260 | 10 | 10 | 60 | 4 | 27 | 0 | 2 |
| OTU261 | 0 | 0 | 0 | 0 | 0 | 5 | 149 |
| OTU262 | 7 | 0 | 0 | 0 | 0 | 0 | 0 |
| OTU263 | 120 | 0 | 0 | 0 | 0 | 0 | 0 |
| OTU264 | 0 | 0 | 0 | 0 | 0 | 0 | 22 |
| OTU265 | 16 | 0 | 0 | 0 | 0 | 0 | 0 |
| OTU266 | 0 | 0 | 0 | 19 | 0 | 0 | 0 |
| OTU267 | 0 | 23 | 0 | 0 | 0 | 0 | 0 |
| OTU268 | 0 | 3 | 0 | 4 | 0 | 0 | 0 |
| OTU269 | 0 | 0 | 0 | 0 | 43 | 0 | 0 |
| OTU270 | 4 | 3 | 0 | 0 | 0 | 0 | 0 |
| OTU271 | 0 | 0 | 0 | 0 | 0 | 0 | 2 |
| OTU272 | 0 | 41 | 0 | 1 | 0 | 0 | 0 |
| OTU273 | 4 | 2 | 0 | 52 | 1 | 11 | 9 |
| OTU274 | 14 | 0 | 0 | 0 | 0 | 0 | 0 |
| OTU275 | 41 | 1 | 0 | 0 | 0 | 0 | 0 |
| OTU276 | 491 | 0 | 0 | 61 | 0 | 0 | 30 |
| OTU277 | 34 | 68 | 43 | 0 | 0 | 0 | 0 |
| OTU278 | 2 | 330 | 1 | 24 | 0 | 0 | 0 |
| OTU279 | 0 | 0 | 0 | 64 | 0 | 0 | 0 |
| OTU280 | 27 | 0 | 0 | 0 | 0 | 0 | 0 |
| OTU281 | 0 | 0 | 0 | 19 | 0 | 2 | 0 |
| OTU282 | 2 | 0 | 0 | 0 | 0 | 0 | 0 |
| OTU283 | 0 | 0 | 0 | 1 | 0 | 2 | 0 |
| OTU284 | 0 | 0 | 12 | 0 | 0 | 0 | 116 |
| OTU285 | 0 | 0 | 0 | 3 | 0 | 0 | 4 |
| OTU286 | 7 | 0 | 0 | 0 | 0 | 0 | 0 |
| OTU287 | 2 | 0 | 0 | 0 | 0 | 0 | 0 |
| OTU288 | 0 | 0 | 0 | 0 | 0 | 0 | 9 |
| OTU289 | 1 | 3 | 5 | 0 | 0 | 0 | 0 |
| OTU290 | 0 | 0 | 0 | 0 | 0 | 0 | 22 |
| OTU291 | 0 | 0 | 0 | 13 | 0 | 1 | 0 |
| OTU292 | 4 | 1 | 0 | 1 | 0 | 40 | 0 |
| OTU293 | 0 | 0 | 0 | 0 | 1 | 2 | 0 |
| OTU294 | 0 | 0 | 0 | 0 | 0 | 1 | 4 |
| OTU295 | 0 | 0 | 0 | 3 | 0 | 0 | 0 |
| OTU296 | 0 | 0 | 2 | 0 | 0 | 0 | 19 |
| OTU297 | 1 | 0 | 31 | 0 | 0 | 0 | 5 |
| OTU298 | 0 | 93 | 0 | 1 | 0 | 0 | 0 |
| OTU299 | 57 | 0 | 0 | 303 | 0 | 0 | 0 |
| OTU300 | 6 | 0 | 0 | 0 | 0 | 0 | 0 |
| OTU301 | 0 | 0 | 0 | 5 | 0 | 0 | 0 |
| OTU302 | 0 | 2 | 0 | 0 | 0 | 9 | 2150 |
| OTU303 | 0 | 6 | 0 | 0 | 0 | 0 | 0 |
| OTU304 | 0 | 5 | 0 | 0 | 0 | 0 | 0 |
| OTU305 | 0 | 29 | 0 | 2 | 0 | 0 | 0 |
| OTU306 | 6 | 0 | 0 | 0 | 0 | 0 | 0 |
| OTU307 | 11 | 0 | 0 | 1 | 0 | 0 | 2 |
| OTU308 | 0 | 0 | 0 | 20 | 0 | 0 | 0 |
| OTU309 | 0 | 0 | 0 | 14 | 0 | 0 | 0 |
| OTU310 | 0 | 0 | 581 | 0 | 1 | 0 | 1 |
| OTU311 | 3 | 0 | 0 | 8 | 0 | 0 | 2 |
| OTU312 | 0 | 0 | 0 | 0 | 0 | 0 | 2 |
| OTU313 | 0 | 0 | 0 | 0 | 0 | 0 | 9 |
| OTU314 | 0 | 0 | 0 | 1 | 0 | 3 | 16 |
| OTU315 | 0 | 9 | 0 | 0 | 0 | 0 | 0 |
| OTU316 | 54 | 4 | 0 | 0 | 0 | 0 | 123 |
| OTU317 | 0 | 20 | 0 | 0 | 0 | 0 | 0 |
| OTU318 | 0 | 0 | 0 | 0 | 0 | 9 | 0 |
| OTU319 | 0 | 0 | 0 | 71 | 0 | 0 | 0 |
| OTU320 | 0 | 0 | 0 | 21 | 0 | 0 | 0 |
| OTU321 | 1 | 2 | 0 | 18 | 0 | 0 | 0 |
| OTU322 | 3 | 0 | 0 | 0 | 0 | 0 | 0 |
| OTU323 | 64 | 0 | 0 | 0 | 0 | 5 | 2 |
| OTU324 | 93 | 0 | 1 | 98 | 0 | 1 | 128 |
| OTU325 | 0 | 6 | 0 | 0 | 0 | 0 | 0 |
| OTU326 | 0 | 0 | 0 | 0 | 0 | 0 | 3 |
| OTU327 | 42 | 15 | 0 | 0 | 5 | 480 | 0 |
| OTU328 | 0 | 1 | 0 | 4 | 0 | 0 | 2 |
| OTU329 | 0 | 0 | 0 | 0 | 0 | 0 | 11 |
| OTU330 | 52 | 0 | 0 | 3 | 0 | 10 | 0 |
| OTU331 | 9 | 3 | 1 | 0 | 0 | 0 | 0 |
| OTU332 | 0 | 0 | 0 | 23 | 0 | 0 | 0 |
| OTU333 | 0 | 0 | 0 | 3 | 0 | 0 | 0 |
| OTU334 | 0 | 0 | 0 | 0 | 0 | 0 | 15 |
| OTU335 | 11 | 0 | 0 | 8 | 0 | 603 | 0 |
| OTU336 | 2 | 0 | 0 | 0 | 0 | 0 | 0 |
| OTU337 | 0 | 2 | 9 | 0 | 0 | 1 | 0 |
| OTU338 | 1 | 0 | 0 | 23 | 0 | 7 | 0 |
| OTU339 | 12 | 4 | 0 | 2 | 0 | 0 | 0 |
| OTU340 | 0 | 61 | 0 | 1 | 0 | 0 | 0 |
| OTU341 | 0 | 0 | 0 | 9 | 0 | 0 | 3 |
| OTU342 | 0 | 1 | 0 | 42 | 3 | 0 | 0 |
| OTU343 | 6 | 17 | 25 | 2 | 14 | 0 | 0 |
| OTU344 | 0 | 3 | 2 | 0 | 0 | 0 | 0 |
| OTU345 | 0 | 0 | 0 | 0 | 0 | 0 | 150 |
| OTU346 | 0 | 200 | 5 | 0 | 0 | 1 | 0 |
| OTU347 | 0 | 14 | 0 | 0 | 0 | 0 | 0 |
| OTU348 | 477 | 328 | 5 | 832 | 0 | 36 | 0 |
| OTU349 | 0 | 50 | 0 | 0 | 0 | 0 | 0 |
| OTU350 | 0 | 0 | 0 | 0 | 0 | 5 | 0 |
| OTU351 | 0 | 1 | 0 | 0 | 3 | 74 | 0 |
| OTU352 | 0 | 6 | 0 | 95 | 0 | 0 | 0 |
| OTU353 | 0 | 0 | 0 | 0 | 0 | 0 | 5 |
| OTU354 | 0 | 0 | 0 | 0 | 0 | 0 | 9 |
| OTU355 | 0 | 10 | 148 | 4 | 0 | 0 | 0 |
| OTU356 | 0 | 1 | 0 | 13 | 0 | 0 | 0 |
| OTU357 | 2 | 3 | 0 | 0 | 12 | 0 | 3 |
| OTU358 | 0 | 0 | 2 | 0 | 0 | 0 | 0 |
| OTU359 | 0 | 0 | 0 | 0 | 8 | 0 | 26 |
| OTU360 | 0 | 1 | 0 | 0 | 0 | 0 | 5 |
| OTU361 | 7 | 0 | 0 | 0 | 0 | 0 | 0 |
| OTU362 | 0 | 22 | 195 | 1 | 0 | 0 | 0 |
| OTU363 | 0 | 0 | 0 | 0 | 0 | 2 | 59 |
| OTU364 | 0 | 0 | 0 | 0 | 0 | 6 | 0 |
| OTU365 | 0 | 0 | 0 | 0 | 0 | 0 | 13 |
| OTU366 | 0 | 0 | 0 | 0 | 0 | 41 | 0 |
| OTU367 | 0 | 5 | 0 | 1 | 0 | 0 | 0 |
| OTU368 | 6 | 0 | 0 | 0 | 0 | 0 | 0 |
| OTU369 | 14 | 0 | 0 | 0 | 0 | 0 | 0 |
| OTU370 | 0 | 8 | 0 | 98 | 0 | 0 | 0 |

**Table S3 |** Information on the 370 OTUs found in the 24 lichen samples, including their frequency, number of reads, identification, and BLASTn top hits with accession numbers in GenBank.

| **OUT ID** | **Freq.** | **Reads** | **phylum** | **Order** | **Family** | **Identification** | **Similarity** | **Accession** | **Close GenBank match** | **Originally reported habitat** |
| --- | --- | --- | --- | --- | --- | --- | --- | --- | --- | --- |
| 1 | 2 | 100 | Ascomycota | Unassigned | Unassigned | *Ascomycota sp.* | 100% (447/447) | [JQ759518](http://www.ncbi.nlm.nih.gov/nucleotide/57869246?report=genbank&log$=nucltop&blast_rank=1&RID=9R00Y6YM014) | *Dothideomycetes sp.*^§^ | Unreported |
| 2 | 10 | 1112 | Ascomycota | Unassigned | Unassigned | *Ascomycota sp.* | 98%(514/526) | [AM901846](http://www.ncbi.nlm.nih.gov/nucleotide/289190145?report=genbank&log$=nucltop&blast_rank=1&RID=9R00Y6YM014) | *Uncultured ascomycete clone*^§^ | House dust in Finland |
| 3 | 2 | 99 | Ascomycota | Chaetothyriales | Herpotrichiellaceae | *Capronia sp.* ^#^ | 99%(455/460) | KC965221 | *Uncultured fungus clone*^§^ | Arctic soil in Canada (78.78N; 103.55W) |
|  |  |  |  |  |  |  | 97%(446/458) | EU139159 | *Capronia sp.* ^§^ | Plant in China (Jilin) |
| 4 | 1 | 17 | Ascomycota | Unassigned | Unassigned | *Ascomycota sp.* | 97%(469/483) | KC966232 | *Uncultured fungus clone*^§^ | Arctic soil in Canada (78.78N; 103.55W) |
| 5 | 1 | 12 | Ascomycota | Helotiales | Unassigned | *Phialocephala sp.* ^#^ | 98%(400/408) | [FR774054](http://www.ncbi.nlm.nih.gov/nucleotide/315270612?report=genbank&log$=nucltop&blast_rank=1&RID=9R00Y6YM014) | *Uncultured Phialocephala clone*^§^ | Leaf surface |
| 6 | 1 | 18 | Ascomycota | Chaetothyriales | Herpotrichiellaceae | *Rhinocladiella sp.* ^#^ | 96%(547/561) | [KC965227](http://www.ncbi.nlm.nih.gov/nucleotide/307750658?report=genbank&log$=nucltop&blast_rank=1&RID=9R00Y6YM014) | *Uncultured fungus clone* | Arctic soil in Canada (76.23N; 119.30W) |
|  |  |  |  |  |  |  | 99%(508/515) | FJ948175 | *Rhinocladiella sp.* ^§^ | Rock in China |
| 7 | 10 | 119 | Ascomycota | Pleosporales | Venturiaceae | *Venturia sp.* ^#^ | 100% (551/551) | [AB916509](http://www.ncbi.nlm.nih.gov/nucleotide/387966447?report=genbank&log$=nucltop&blast_rank=1&RID=A1G7PDYP01R) | *Venturia sp.* ^§^ | Arctic bird feather in Norway |
| 8 | 1 | 2 | Ascomycota | Teloschistales | Teloschistaceae | *Paryoplaca athallina** | 99%(500/502) | KC966222 | *Uncultured fungus clone*^§^ | Arctic soil in Canada (78.78N; 103.55W) |
|  |  |  |  |  |  |  | 99%(453/456) | KC179111 | *Parvoplaca athallina*^§^ | Lichen in Antarctica |
| 9 | 10 | 39 | Ascomycota | Pleosporales | Unassigned | *Pleosporales sp.* | 100% (498/498) | [GU817159](http://www.ncbi.nlm.nih.gov/nucleotide/189007731?report=genbank&log$=nucltop&blast_rank=1&RID=A1G7PDYP01R) | *Uncultured fungus clone*^§^ | Plant root in Svalbard |
|  |  |  |  |  |  |  | 99%(475/478) | AB751503 | *Pleosporales sp.* ^§^ | Plant in high- arctic Canada |
| 10 | 2 | 17 | Ascomycota | Baeomycetales | Baeomycetaceae | *Baeomyces rufus** | 99%(501/502) | AF448458 | *Baeomyces rufus*^§^ | Lichen |
| 11 | 3 | 773 | Ascomycota | Unassigned | Unassigned | *Ascomycota sp.* | 97%(501/519) | [KC966013](http://www.ncbi.nlm.nih.gov/nucleotide/307750688?report=genbank&log$=nucltop&blast_rank=1&RID=A1G7PDYP01R) | *Uncultured fungus clone*^§^ | Arctic soil in Canada (76.23N; 119.30W) |
|  |  |  |  |  |  |  | 83%(379/456) | AM901730 | *Uncultured ascomycete clone* | House dust in Finland |
| 12 | 4 | 30 | Ascomycota | Unassigned | Unassigned | *Ascomycota sp.* | 88%(380/434) | [HM239990](http://www.ncbi.nlm.nih.gov/nucleotide/194304360?report=genbank&log$=nucltop&blast_rank=3&RID=9R00Y6YM014) | *Uncultured Ascomycota clone* | Grassland soil in USA (California) |
| 13 | 1 | 4 | Ascomycota | Unassigned | Unassigned | *Ascomycota sp.* | 85%(460/544) | KF800446 | *Uncultured fungus clone* | House dust in USA |
|  |  |  |  |  |  |  | 84%(441/528) | KF227874 | *Pezizomycotina sp.* | Semi evergreen vine thickets |
| 14 | 2 | 21 | Ascomycota | Thelebolales | Thelebolaceae | *Pezizella discreta** | 98%(522/531) | [JF908571](http://www.ncbi.nlm.nih.gov/nucleotide/319826960?report=genbank&log$=nucltop&blast_rank=10&RID=9R00Y6YM014) | *Pezizella discreta*^§^ | Unreported |
| 15 | 1 | 432 | Ascomycota | Capnodiales | Teratosphaeriaceae | *Teratosphaeriaceae sp.* | 94%(425/453) | [EU490102](http://www.ncbi.nlm.nih.gov/nucleotide/387966434?report=genbank&log$=nucltop&blast_rank=2&RID=9R00Y6YM014) | *Uncultured ascomycete clone* | Savanna soil in USA (33.85N; 99.44W) |
|  |  |  |  |  |  |  | 93%(416/448) | EU707864 | *Teratosphaeria jonkershoekensis* | Plant in South Africa |
| 16 | 1 | 3 | Ascomycota | Helotiales | Unassigned | *Helotiales sp.* | 99%(488/489) | KC965927 | *Uncultured fungus clone*^§^ | Arctic soil in Canada (76.23N; 119.30W) |
|  |  |  |  |  |  |  | 94%(447/474) | FJ196296 | *Helotiales sp.* | Unreported |
| 17 | 1 | 67 | Ascomycota | Unassigned | Unassigned | *Ascomycota sp.* | 93%(224/242) | [KF296868](http://www.ncbi.nlm.nih.gov/nucleotide/307750518?report=genbank&log$=nucltop&blast_rank=1&RID=9R00Y6YM014) | *Uncultured fungus clone* | Arctic soil in Canada (73.22N; 119.56W) |
|  |  |  |  |  |  |  | 84%(268/319) | AB693770 | *Leptosphaeria sp.* | Unreported |
| 18 | 1 | 9 | Ascomycota | Helotiales | Unassigned | *Helotiales sp.* | 92%(479/520) | [HM488536](http://www.ncbi.nlm.nih.gov/nucleotide/296785201?report=genbank&log$=nucltop&blast_rank=1&RID=9R00Y6YM014) | *Uncultured Helotiales clone* | Ectomycorrhiza in forest |
| 19 | 8 | 71 | Ascomycota | Dothideales | Dothidoraceae | *Dothidoraceae sp.* | 92%(449/488) | [KF274441](http://www.ncbi.nlm.nih.gov/nucleotide/326319947?report=genbank&log$=nucltop&blast_rank=1&RID=A1G7PDYP01R) | *Uncultured fungus clone* | Wood stump in Finland |
|  |  |  |  |  |  |  | 90%(478/534) | KJ690089 | *Aureobasidium sp.* | Ectomycorrhiza |
| 20 | 1 | 227 | Ascomycota | Helotiales | Unassigned | *Helotiales sp.* | 96%(403/419) | KF274408 | *Uncultured fungus clone* | Wood stump in Finland |
|  |  |  |  |  |  |  | 96%(338/353) | EU726289 | *Uncultured Helotiales clone* | Ectomycorrhiza in USA |
| 21 | 3 | 45 | Basidiomycota | Tremelalles | Unassigned | *Tremellales sp.* | 93%(463/499) | [GU993519](http://www.ncbi.nlm.nih.gov/nucleotide/57869255?report=genbank&log$=nucltop&blast_rank=1&RID=A1G7PDYP01R) | *Uncultured Tremellales clone* | Tower in Brazial |
| 22 | 1 | 14 | Ascomycota | Chaetothyriales | Herpotrichiellaceae | *Herpotrichiellaceae sp.* | 100%(537/537) | [FJ552708](http://www.ncbi.nlm.nih.gov/nucleotide/315270612?report=genbank&log$=nucltop&blast_rank=1&RID=9R00Y6YM014) | *Uncultured Herpotrichiellaceae clone*^§^ | Forest soil in Canada |
| 23 | 3 | 147 | Basidiomycota | Sebacinales | Sebacinaceae | *Sebacina sp.* ^#^ | 98%(493/504) | [GQ907137](http://www.ncbi.nlm.nih.gov/nucleotide/19913082?report=genbank&log$=nucltop&blast_rank=1&RID=9R00Y6YM014) | *Uncultured Sebacina clone*^§^ | Liverwort in Chile |
| 24 | 1 | 2 | Ascomycota | Unassigned | Unassigned | *Ascomycota sp.* | 96%(406/423) | KF274449 | *Uncultured fungus clone* | Forest soil in Canada |
|  |  |  |  |  |  |  | 84%(355/424) | KJ462276 | *Xylographa bjoerkii* | Unreported |
| 25 | 3 | 14 | Ascomycota | Helotiales | Unassigned | *Helotiales sp.* | 99%(481/487) | [KC965195](http://www.ncbi.nlm.nih.gov/nucleotide/387145937?report=genbank&log$=nucltop&blast_rank=1&RID=9R00Y6YM014) | *Uncultured fungus clone*^§^ | Arctic soil in USA (69.67N; 148.72W) |
|  |  |  |  |  |  |  | 95%(485/512) | JN859275 | *Helotiales sp.* | Plant root in Hungary |
| 26 | 1 | 4 | Ascomycota | Unassigned | Unassigned | *Ascomycota sp.* | 84%(352/417) | GU174330 | *Uncultured fungus clone* | Wood stump in Finland |
|  |  |  |  |  |  |  | 82%(349/426) | AM901939 | *Uncultured ascomycete clone* | House dust in Finland |
| 27 | 5 | 327 | Ascomycota | Verrucariales | Verrucariaceae | *Polyblastia terrestis** | 99%(516/519) | EU364560 | *Polyblastia terrestis*^§^ | Arctic soil in USA (69.67N; 148.72W) |
| 28 | 10 | 132 | Ascomycota | Pleosporales | Unassigned | *Pleosporales sp.* | 91%(441/486) | [HG327911](http://www.ncbi.nlm.nih.gov/nucleotide/387966464?report=genbank&log$=nucltop&blast_rank=1&RID=9R00Y6YM014) | *Uncultured fungus clone* | Arable soil in China (Gongzhuling) |
|  |  |  |  |  |  |  | 88%(457/519) | FJ210521 | *Preussia sp.* | Leaf in USA (Minnesota) |
| 29 | 1 | 10 | Unassigned | Unassigned | Unassigned | *Fungus sp.* | 99%(454/460) | KC966180 | *Uncultured fungus clone*^§^ | Arctic soil in USA (69.67N; 148.72W) |
| 30 | 1 | 9 | Ascomycota | Chaetothyriales | Herpotrichiellaceae | *Herpotrichiellaceae sp.* | 95%(520/546) | [FN397279](http://www.ncbi.nlm.nih.gov/nucleotide/194304253?report=genbank&log$=nucltop&blast_rank=1&RID=9R00Y6YM014) | *Uncultured fungus clone* | Soil in France (44.26N; 1.26E) |
|  |  |  |  |  |  |  | 93%(503/541) | EU035420 | *Exophiala sp* | Plant in Canada |
| 31 | 3 | 9 | Ascomycota | Chaetothyriales | Herpotrichiellaceae | *Herpotrichiellaceae sp.* | 97%(507/524) | [KC965720](http://www.ncbi.nlm.nih.gov/nucleotide/157057286?report=genbank&log$=nucltop&blast_rank=1&RID=9R00Y6YM014) | *Uncultured fungus clone*^§^ | Arctic soil in Canada (78.78N; 103.55W) |
|  |  |  |  |  |  |  | 94%(517/548) | EF016381 | *Cladophialophora minutissima* | Bryophyte |
| 32 | 2 | 159 | Basidiomycota | Tremellales | Unassigned | *Cryptococcus sp.* ^#^ | 97%(305/314) | [KC753404](http://www.ncbi.nlm.nih.gov/nucleotide/387966464?report=genbank&log$=nucltop&blast_rank=1&RID=9R00Y6YM014) | *Uncultured Cryptococcus clone*^§^ | Grain in Sweden |
| 33 | 7 | 21 | Ascomycota | Thelebolales | Thelebolaceae | *Thelobolus globosus** | 100%(492/492) | JX171196 | *Thelobolus globosus*^§^ | Antarctica |
| 34 | 1 | 9 | Ascomycota | Capnodiales | Teratosphaeriaceae | *Teratosphaeriaceae sp.* | 95%(498/524) | [KC](http://www.ncbi.nlm.nih.gov/nucleotide/387966464?report=genbank&log$=nucltop&blast_rank=1&RID=9R00Y6YM014)966158 | *Unculturedfungus clone* | Arctic soil in USA (69.15N; 148.85W) |
|  |  |  |  |  |  |  | 89%(499/558) | EU707864 | *Teratosphaeria jonkershoekensis* | Plant in South Africa |
| 35 | 8 | 587 | Ascomycota | Helotiales | Helotiaceae | *Helotiaceae sp.* | 99%(487/488) | [KF297173](http://www.ncbi.nlm.nih.gov/nucleotide/305671334?report=genbank&log$=nucltop&blast_rank=1&RID=9R00Y6YM014) | *Uncultured fungus clone*^§^ | Arctic soil in Canada (78.78N; 103.55W) |
|  |  |  |  |  |  |  | 90%(468/520) | HQ533008 | *Claussenomyces sp.* | New Zealand |
| 36 | 1 | 7 | Ascomycota | Unassigned | Unassigned | *Ascomycota sp.* | 97%(483/496) | [KC966265](http://www.ncbi.nlm.nih.gov/nucleotide/99867326?report=genbank&log$=nucltop&blast_rank=1&RID=9R00Y6YM014) | *Uncultured fungus clone*^§^ | Arctic soil in Canada (73.22N; 119.56W) |
|  |  |  |  |  |  |  | 87%(434/501) | AM292201 | *Ascomycete sp.* | Peat in United Kingdom |
| 37 | 2 | 12 | Ascomycota | Helotiales | Vibrisseaceae | *Vibrisseaceae sp.* | 99%(487/491) | KC965908 | *Uncultured fungus clone*^§^ | Arctic soil in Canada (78.78N;103.55W) |
|  |  |  |  |  |  |  | 90%(459/509) | JX415338 | *Vibrissea filisporia* | Unreported |
| 38 | 2 | 26 | Ascomycota | Helotiales | Unassigned | *Helotiales sp.* | 97%(503/517) | [HM069356](http://www.ncbi.nlm.nih.gov/nucleotide/85679810?report=genbank&log$=nucltop&blast_rank=2&RID=9R00Y6YM014) | *Uncultured fungus clone*^§^ | Pine forest soil |
|  |  |  |  |  |  |  | 96%(460/478) | FJ196296 | *Helotiales sp.* | Mycorrhiza in Taiwan |
| 39 | 1 | 302 | Ascomycota | Lecideales | Lecideaceae | *Lecideaceae sp.* | 91%(503/555) | HQ650671 | *Lecidea tessellata* | Unreported |
| 40 | 1 | 5 | Ascomycota | Lecanorales | Unassigned | *Lecanorales sp.* | 89%(420/474) | [DQ219307](http://www.ncbi.nlm.nih.gov/nucleotide/27497500?report=genbank&log$=nucltop&blast_rank=1&RID=9R00Y6YM014) | *Usnea aff. igniaria* | Unreported |
| 41 | 6 | 595 | Basidiomycota | Tremellales | Unassigned | *Cryptococcus sp.* ^#^ | 99%(504/507) | KC753404 | *Uncultured Cryptococcus clone*^§^ | Grain in Sweden |
| 42 | 5 | 622 | Ascomycota | Unassigned | Unassigned | *Ascomycota sp.* | 99%(530/533) | KC965805 | *Uncultured fungus clone*^§^ | Arctic soil in Canada (76.23N; 119.30W) |
|  |  |  |  |  |  |  | 91%(471/518) | EF373563 | *Ascomycete sp.* | Endolichenic fungi in lichen |
| 43 | 2 | 10 | Ascomycota | Helotiales | Unassigned | *Helotiales sp.* | 97%(505/522) | [GQ268559](http://www.ncbi.nlm.nih.gov/nucleotide/307750624?report=genbank&log$=nucltop&blast_rank=1&RID=9R00Y6YM014) | *Uncultured Helotiales clone*^§^ | Ectomycorrhizal root in Malaysia |
| 44 | 2 | 79 | Ascomycota | Helotiales | Unassigned | *Helotiales sp.* | 98%(513/518) | [JN032499](http://www.ncbi.nlm.nih.gov/nucleotide/290575810?report=genbank&log$=nucltop&blast_rank=1&RID=A1G7PDYP01R) | *Uncultured fungus clone*^§^ | Moss litter from coniferous forest |
|  |  |  |  |  |  |  | 98%(448/456) | AY465452 | *Helotiales sp.* ^§^ | Surface-sterilized needle in USA |
| 45 | 2 | 109 | Ascomycota | Chaetothyriales | Herpotrichiellaceae | *Herpotrichiellaceae sp.* | 91%(489/537) | [HQ215794](http://www.ncbi.nlm.nih.gov/nucleotide/307750650?report=genbank&log$=nucltop&blast_rank=1&RID=A1G7PDYP01R) | *Uncultured Herpotrichiellaceae clone* | Forest soil in Canada |
| 46 | 1 | 3 | Ascomycota | Helotiales | Unassigned | *Phialocephala flargerbergii** | 99%(496/497) | KF617341 | *Uncultured fungus clone*^§^ | Forest soil in USA (64.91N; 147.82W) |
|  |  |  |  |  |  |  | 100%(490/490) | AB190400 | *Phialocephala largerbergii*^§^ | Unreported |
| 47 | 1 | 6 | Basidiomycota | Unassigned | Unassigned | *Basidiomycota sp.* | 92%(503/548) | [KC965773](http://www.ncbi.nlm.nih.gov/nucleotide/61608468?report=genbank&log$=nucltop&blast_rank=1&RID=A1G7PDYP01R) | *Uncultured fungus clone* | Arctic soil in Canada (76.23N; 119.30W) |
|  |  |  |  |  |  |  | 87%(505/581) | AM902000 | *Uncultured basidiomycete clone* | House dust in Finland |
| 48 | 2 | 6 | Ascomycota | Thelebolales | Thelebolaceae | *Thelebolaceae sp.* | 95%(490/518) | [FM178231](http://www.ncbi.nlm.nih.gov/nucleotide/326319947?report=genbank&log$=nucltop&blast_rank=1&RID=9R00Y6YM014) | *Uncultured Thelebolaceae clone* | Malthouse waste water |
| 49 | 2 | 182 | Ascomycota | Lecanorales | Ramalinaceae | *Bilimbia microcarpa** | 99%(396/402) | AM292669 | *Bilimbia microcarpa*^§^ | Unreported |
| 50 | 2 | 18 | Ascomycota | Helotiales | Unassigned | *Helotiales sp.* | 99%(513/519) | [FJ378851](http://www.ncbi.nlm.nih.gov/nucleotide/307750666?report=genbank&log$=nucltop&blast_rank=1&RID=9R00Y6YM014) | *Uncultured Helotiales clone*^§^ | Ectomycorrhiza in Himalaya |
| 51 | 6 | 493 | Ascomycota | Helotiales | Unassigned | *Helotiales sp.* | 99%(499/506) | [KF274367](http://www.ncbi.nlm.nih.gov/nucleotide/344333352?report=genbank&log$=nucltop&blast_rank=1&RID=9R00Y6YM014) | *Uncultured fungus clone*^§^ | Wood stump in Finland |
|  |  |  |  |  |  |  | 97%(480/493) | HQ608110 | *Helotiales sp.* ^§^ | Ants nest in USA |
| 52 | 5 | 925 | Ascomycota | Helotiales | Helotiaceae | *Helotiaceae sp.* | 90%(433/481) | FJ440900 | *Uncultured Helotiaceae clone* | Roots of Pyrola plants in USA |
| 53 | 2 | 4 | Zygomycota | Mortierellales | Mortierellaceae | *Mortierella sp.* ^#^ | 99%(495/496) | [FN565295](http://www.ncbi.nlm.nih.gov/nucleotide/307750670?report=genbank&log$=nucltop&blast_rank=1&RID=9R00Y6YM014) | *Uncultured Mortierella clone*^§^ | Plant root in United Kingdom |
| 54 | 3 | 354 | Unassigned | Unassigned | Unassigned | *Fungus sp.* | 87%(348/398) | KF296943 | *Uncultured fungus clone* | Arctic soil in Canada (76.23N; 119.30W) |
| 55 | 2 | 9 | Ascomycota | Lecanorales | Lecanoraceae | *Carbonea sp.* | 96%(392/408) | JX036120 | *Carbonea sp.* | Antarctica (McMurdo Dry Valleys) |
| 56 | 1 | 4 | Basidiomycota | Malasseziales | Malasseziaceae | *Malassezia sp.* | 100%(406/406) | AM901732 | *Uncultured basidiomycete clone*^§^ | Hust dust in Finland |
|  |  |  |  |  |  |  | 95%(381/401) | KC785582 | *Uncultured Malassezia clone* | Soil in Antarctica |
| 57 | 5 | 326 | Ascomycota | Helotiales | Helotiaceae | *Rhizoscyphus sp.* ^#^ | 99%(521/524) | [GU997743](http://www.ncbi.nlm.nih.gov/nucleotide/28974813?report=genbank&log$=nucltop&blast_rank=1&RID=9R00Y6YM014) | *Uncultured Rhizoscyphus clone*^§^ | Ectomycorrhiza in USA (Arctic,68.63N 149.57W) |
| 58 | 1 | 10 | Ascomycota | Pleosporales | Melanommataceae | *Herpotrichia juniperi** | 98% (484/495) | [DQ420833](http://www.ncbi.nlm.nih.gov/nucleotide/90200597?report=genbank&log$=nucltop&blast_rank=1&RID=A1G7PDYP01R) | *Uncultured soil fungus clone*^§^ | Soil in USA |
|  |  |  |  |  |  |  | 98%(448/456) | JX981496 | *Herpotrichia juniperi*^§^ | Plant in Poland |
| 59 | 2 | 6 | Basidiomycota | Erythrobasidiales | Erythrobasidiaceae | *Rhodotorula lamellibrachiae* | 92%(450/488) | KF617929 | *Uncultured fungus clone* | Forest soil in USA |
|  |  |  |  |  |  |  | 88%(458/519) | AB263122 | *Rhodotorula lamellibrachiae* | Japan |
| 60 | 2 | 213 | Basidiomycota | Agaricales | Tricholomataceae | *Tricholomataceae sp.* | 99%(477/478) | [KC966326](http://www.ncbi.nlm.nih.gov/nucleotide/387966434?report=genbank&log$=nucltop&blast_rank=1&RID=A1G7PDYP01R) | *Uncultured fungus clone*^§^ | Arctic soil in Canada (78.78N; 103.55W) |
|  |  |  |  |  |  |  | 92%(442/483) | AB777495 | *Uncultured Tricholomataceae clone* | Mycorrhizal root tip in Japan |
| 61 | 1 | 5 | Ascomycota | Trapeliales | Trapeliaceae | *Trapeliopsis bisorediata* | 91%(393/431) | [KF274365](http://www.ncbi.nlm.nih.gov/nucleotide/289190130?report=genbank&log$=nucltop&blast_rank=1&RID=A1G7PDYP01R) | *Uncultured fungus clone* | Wood stump in Finland |
|  |  |  |  |  |  |  | 100%(374/374) | AF353563 | *Trapeliopsis bisorediata*^§^ | Lichen in Western North America |
| 62 | 2 | 6 | Ascomycota | Helotiales | Unassigned | *Helotiales sp.* | 98%(497/508) | HM230871 | *Uncultured Leotiomycetes clone*^§^ | Cavendishioid mycorrhiza in Ecuador |
|  |  |  |  |  |  |  | 96%(485/507) | FJ827188 | *Uncultured Helotiales clone* | Ectomycorrhiza in China |
| 63 | 2 | 7 | Basidiomycota | Tremellales | Tremellaceae | *Tremellaceae sp.* | 93%(412/445) | [JN790586](http://www.ncbi.nlm.nih.gov/nucleotide/344333452?report=genbank&log$=nucltop&blast_rank=1&RID=9R00Y6YM014) | *Tremella diploschistina* | Lichenicolous fungi on Diploschistes |
| 64 | 1 | 82 | Ascomycota | Pertusariales | Megasporaceae | *Megasporaceae sp.* | 94%(455/486) | EU057953 | *Aspicillia zonata* | Sweden |
| 65 | 9 | 206 | Ascomycota | Unassigned | Unassigned | *Ascomycota sp.* | 98%(447/457) | [KC966342](http://www.ncbi.nlm.nih.gov/nucleotide/99866600?report=genbank&log$=nucltop&blast_rank=1&RID=9R00Y6YM014) | *Uncultured fungus clone*^§^ | Arctic soil in Canada (76.23N; 119.30W) |
|  |  |  |  |  |  |  | 88%(382/432) | EF373563 | *Ascomycete sp.* | Endolichenic fungi in lichen |
| 66 | 2 | 15 | Basidiomycota | Sebacinales | Sebacinaceae | *Sebacina sp.* | 95%(477/501) | [GQ907137](http://www.ncbi.nlm.nih.gov/nucleotide/19913082?report=genbank&log$=nucltop&blast_rank=1&RID=9R00Y6YM014) | *Uncultured Sebacina mycobiont* | Liverwort in Chile |
| 67 | 1 | 2 | Ascomycota | Baeomycetales | Unassigned | *Baeomycetales sp.* | 90%(392/434) | KF274449 | *Uncultured fungus clone* | Wood stump in Finland |
|  |  |  |  |  |  |  | 89%(379/428) | FJ903295 | *Sarea difformis* | Plant stump in Latvia |
| 68 | 2 | 258 | Ascomycota | Unassigned | Unassigned | *Ascomycota sp.* | 98%(523/535) | KC965473 | *Uncultured fungus clone*^§^ | Arctic soil in Canada (73.22N; 119.56W) |
|  |  |  |  |  |  |  | 83%(252/302) | KF513524 | *Dothideomycetes sp.* | Rock in China |
| 69 | 1 | 4 | Ascomycota | Capnodiales | Teratosphaeriaceae | *Teratosphaeriaceae sp.* | 94%(338/358) | [EU490102](http://www.ncbi.nlm.nih.gov/nucleotide/124441653?report=genbank&log$=nucltop&blast_rank=1&RID=9R00Y6YM014) | *Uncultured ascomycete clone* | Savanna soil in USA (33.85N; 99.44W) |
|  |  |  |  |  |  |  | 93%(335/359) | AJ971406 | *Capnobotryella sp.* | Marble monument in Turkey |
| 70 | 1 | 7 | Ascomycota | Verrucariales | Verrucariaceae | *Polyblastia sendtneri** | 99%(505/512) | [KC965812](http://www.ncbi.nlm.nih.gov/nucleotide/289190130?report=genbank&log$=nucltop&blast_rank=1&RID=A1G7PDYP01R) | *Uncultured fungus clone*^§^ | Arctic soil in Canada (73.22N; 119.56W) |
|  |  |  |  |  |  |  | 100%(486/486) | EU559741 | *Polyblastia sendtneri*^§^ | Unreported |
| 71 | 2 | 3 | Ascomycota | Lecanorales | Unassigned | *Lecanorales sp.* | 86%(350/405) | [JX466470](http://www.ncbi.nlm.nih.gov/nucleotide/5420284?report=genbank&log$=nucltop&blast_rank=1&RID=9R00Y6YM014) | *Parmelina tiliacea* | Sweden |
| 72 | 3 | 89 | Basidiomycota | Agaricales | Tricholomataceae | *Tricholomataceae sp.* | 92%(462/504) | [U66428](http://www.ncbi.nlm.nih.gov/nucleotide/266634419?report=genbank&log$=nucltop&blast_rank=1&RID=9R00Y6YM014) | *Arrhenia auriscalpium* | Unreported |
| 73 | 2 | 16 | Ascomycota | Unassigned | Unassigned | *Ascomycota sp.* | 99%(523/524) | [KC965952](http://www.ncbi.nlm.nih.gov/nucleotide/117415072?report=genbank&log$=nucltop&blast_rank=1&RID=9R00Y6YM014) | *Uncultured fungus clone*^§^ | Arctic soil in Canada (73.22N; 119.56W) |
|  |  |  |  |  |  |  | 81%(437/541) | JN638252 | *Hydropunctaria amphibia* | Ireland |
| 74 | 8 | 255 | Ascomycota | Helotiales | Unassigned | *Helotiales sp.* | 99%(507/512) | KC965355 | *Uncultured fungus clone*^§^ | Arctic soil in Canada (78.78N; 103.55W) |
|  |  |  |  |  |  |  | 93%(444/477) | EU726289 | *Uncultured Helotiales clone* | Ectomycorrhiza in USA |
| 75 | 2 | 36 | Basidiomycota | Unassigned | Unassigned | *Basidiomycota sp.* | 95%(262/275) | [GU328576](http://www.ncbi.nlm.nih.gov/nucleotide/387966464?report=genbank&log$=nucltop&blast_rank=1&RID=9R00Y6YM014) | *Uncultured Basidiomycota clone* | Oe layer |
| 76 | 2 | 24 | Ascomycota | Unassigned | Unassigned | *Ascomycota sp.* | 92% (400/436) | [KC965705](http://www.ncbi.nlm.nih.gov/nucleotide/99866600?report=genbank&log$=nucltop&blast_rank=1&RID=9R00Y6YM014) | *Uncultured fungus clone* | Arctic soil in USA (69.15N; 148.85W) |
|  |  |  |  |  |  |  | 91%(378/417) | FR773503 | *Uncultured Ascomycota clone* | Plant |
| 77 | 1 | 23 | Unassigned | Unassigned | Unassigned | *Fungus sp.* | 91%(475/520) | [KC965473](http://www.ncbi.nlm.nih.gov/nucleotide/307750675?report=genbank&log$=nucltop&blast_rank=1&RID=9R00Y6YM014) | *Uncultured fungus clone* | Arctic soil in Canada (73.22N,119.56W) |
| 78 | 1 | 3 | Ascomycota | Unassigned | Unassigned | *Ascomycota sp.* | 86%(449/523) | [KF274130](http://www.ncbi.nlm.nih.gov/nucleotide/305671356?report=genbank&log$=nucltop&blast_rank=1&RID=9R00Y6YM014) | *Uncultured fungus clone* | Wood stump in Finland |
|  |  |  |  |  |  |  | 88%(385/440) | HF947891 | *Uncultured Ascomycota clone* | Plant root in Ireland |
| 79 | 2 | 941 | Ascomycota | Unassigned | Unassigned | *Ascomycota sp.* | 91%(472/520) | [FJ553685](http://www.ncbi.nlm.nih.gov/nucleotide/311334607?report=genbank&log$=nucltop&blast_rank=2&RID=9R00Y6YM014) | *Uncultured Leotimycetes clone* | Forest soil in Canada |
| 80 | 5 | 63 | Unassigned | Unassigned | Unassigned | *Fungus sp.* | 99%(518/523) | [KF297187](http://www.ncbi.nlm.nih.gov/nucleotide/238058348?report=genbank&log$=nucltop&blast_rank=1&RID=9R00Y6YM014) | *Uncultured fungus clone*^§^ | Arctic soil in Canada (76.23N,119.30W) |
| 81 | 4 | 124 | Ascomycota | Lecanorales | Porpidiaceae | *Porpidia macrocarpa** | 98%(430/440) | [HQ605940](http://www.ncbi.nlm.nih.gov/nucleotide/22024475?report=genbank&log$=nucltop&blast_rank=1&RID=9R00Y6YM014) | *Porpidia macrocarpa*^§^ | Turkey |
| 82 | 2 | 5 | Ascomycota | Helotiales | Dermeataceae | *Dermea viburni** | 99%(509/511) | [FJ827172](http://www.ncbi.nlm.nih.gov/nucleotide/281190615?report=genbank&log$=nucltop&blast_rank=1&RID=A1G7PDYP01R) | *Uncultured Helotiales clone*^§^ | Ectomycorrhiza in China |
|  |  |  |  |  |  |  | 96%(493/511) | AF141163 | *Dermea viburni* | Unreported |
| 83 | 3 | 13 | Ascomycota | Pertusariales | Megasporaceae | *Aspicillia simoenosis** | 99%(355/359) | AF332115 | *Aspicillia simoensis*^§^ | Unreported |
| 84 | 1 | 3 | Unassigned | Unassigned | Unassigned | *Fungus sp.* | 97%(467/482) | KF297265 | *Uncultured fungus clone*^§^ | Arctic soil in Canada (76.23N;119.30W) |
| 85 | 1 | 4 | Ascomycota | Chaetothyriales | Herpotrichiellaceae | *Cladophialophora sp.* ^#^ | 99%(507/513) | [KC966083](http://www.ncbi.nlm.nih.gov/nucleotide/28974813?report=genbank&log$=nucltop&blast_rank=1&RID=9R00Y6YM014) | *Uncultured fungus clone*^§^ | Arctic soil in Canada (76.23N;119.30W) |
|  |  |  |  |  |  |  | 95%(508/535) | EU035406 | *Cladophialophora chaetospira* | Soil in Germany |
| 86 | 2 | 6 | Ascomycota | Helotiales | Helotiaceae | *Helotiaceae sp.* | 98%(512/525) | [EU668920](http://www.ncbi.nlm.nih.gov/nucleotide/194304253?report=genbank&log$=nucltop&blast_rank=1&RID=9R00Y6YM014) | *Uncultured Helotiaceae clone*^§^ | Host roots in forest soil (Estonia) |
| 87 | 12 | 2422 | Ascomycota | Helotiales | Helotiaceae | *Helotiaceae sp.* | 99%(509/512) | [KF274394](http://www.ncbi.nlm.nih.gov/nucleotide/344333076?report=genbank&log$=nucltop&blast_rank=1&RID=9R00Y6YM014) | *Uncultured fungus clone*^§^ | Wood stump in Finland |
|  |  |  |  |  |  |  | 91%(482/530) | HQ533008 | *Claussenomyces sp.* | New Zealand |
| 88 | 1 | 26 | Basidiomycota | Sebacinales | Sebacinaceae | *Sebacina sp.* ^#^ | 99%(490/496) | [GQ907097](http://www.ncbi.nlm.nih.gov/nucleotide/19913082?report=genbank&log$=nucltop&blast_rank=1&RID=9R00Y6YM014) | *Uncultured Sebacina mycobiont*^§^ | Liverwort in United Kingdom |
| 89 | 1 | 103 | Basidiomycota | Cantharellales | Tulasnellaceae | *Tulasnellaceae sp.* | 97%(416/431) | [DQ925531](http://www.ncbi.nlm.nih.gov/nucleotide/311404310?report=genbank&log$=nucltop&blast_rank=1&RID=9R00Y6YM014) | *Uncultured Tulasnellaceae isolate*^§^ | Plant roots in Japan |
| 90 | 3 | 11 | Ascomycota | Capnodiales | Unassigned | *Capnodiales sp.* | 84%(402/478) | FJ372394 | *Teratosphaeria altensteinii* | Unreported |
| 91 | 1 | 5 | Basidiomycota | Polyporales | Unassigned | *Trechispora sp.* | 95%(395/415) | [JF519107](http://www.ncbi.nlm.nih.gov/nucleotide/305671248?report=genbank&log$=nucltop&blast_rank=1&RID=9R00Y6YM014) | *Uncultured Trechispora clone* | Plant in Austria |
| 92 | 6 | 192 | Ascomycota | Pleosporales | Unassigned | *Atradidymella muscivota** | 99%(515/517) | [EU817829](http://www.ncbi.nlm.nih.gov/nucleotide/344333056?report=genbank&log$=nucltop&blast_rank=1&RID=9R00Y6YM014) | *Atradidymella muscivora*^§^ | Unreported |
| 93 | 1 | 4 | Ascomycota | Lecanorales | Unassigned | *Lecanorales sp.* | 88%(453/516) | [EU075538](http://www.ncbi.nlm.nih.gov/nucleotide/307750688?report=genbank&log$=nucltop&blast_rank=1&RID=9R00Y6YM014) | *Miriguidica garovaqlii* | Unreported |
| 94 | 2 | 140 | Basidiomycota | Tremellales | Unassigned | *Trichosporon loubieri** | 98%(403/412) | [KC254110](http://www.ncbi.nlm.nih.gov/nucleotide/193297393?report=genbank&log$=nucltop&blast_rank=1&RID=9R00Y6YM014) | *Trichosporon loubieri*^§^ | Unreported |
| 95 | 1 | 10 | Basidiomycota | Thelephorales | Thelephoraceae | *Tomentella sp.* ^#^ | 99%(478/480) | FJ554055 | *Uncultured Tomentella clone*^§^ | Forest soil in Canada |
| 96 | 1 | 32 | Ascomycota | Chaetothyriales | Herpotrichiellaceae | *Herpotrichiellaceae sp.* | 93%(497/536) | KF274156 | *Uncultured fungus clone* | Wood stump in Finland |
|  |  |  |  |  |  |  | 91%(492/538) | JQ272383 | *Herpotrichiellaceae sp.* | Plant root in USA |
| 97 | 1 | 14 | Ascomycota | Lecanorales | Porpidiaceae | *Porpidiaceae sp.* | 91%(461/506) | [HQ605939](http://www.ncbi.nlm.nih.gov/nucleotide/14571573?report=genbank&log$=nucltop&blast_rank=1&RID=A1G7PDYP01R) | *Porpidia musiva* | Turkey |
| 98 | 2 | 8 | Ascomycota | Unassigned | Unassigned | *Ascomycota sp.* | 94%(446/473) | [KF297261](http://www.ncbi.nlm.nih.gov/nucleotide/27497504?report=genbank&log$=nucltop&blast_rank=1&RID=9R00Y6YM014) | *Uncultured fungus clone* | Arctic soil in Canada (76.23N; 119.30W) |
|  |  |  |  |  |  |  | 94%(420/449) | FJ008690 | *Ascomycota sp.* | Endolichenic fungi |
| 99 | 1 | 5 | Ascomycota | Chaetothyriales | Herpotrichiellaceae | *Cladophialophora humicolae** | 99%(546/551) | [EU035408](http://www.ncbi.nlm.nih.gov/nucleotide/344332975?report=genbank&log$=nucltop&blast_rank=1&RID=9R00Y6YM014) | *Cladophialophora humicolae*^§^ | Arable soil in Germany |
| 100 | 1 | 4 | Ascomycota | Unassigned | Unassigned | *Ascomycota sp.* | 85%(439/518) | [EU552154](http://www.ncbi.nlm.nih.gov/nucleotide/2289066?report=genbank&log$=nucltop&blast_rank=1&RID=9R00Y6YM014) | *Rhynchostoma proteae* | South Africa |
| 101 | 2 | 99 | Ascomycota | Capnodiales | Teratosphaeriaceae | *Capnobotryella sp.* | 95%(462/485) | [KC966173](http://www.ncbi.nlm.nih.gov/nucleotide/27497500?report=genbank&log$=nucltop&blast_rank=1&RID=9R00Y6YM014) | *Uncultured fungus clone* | Arctic soil in Canada (76.23N; 119.30W) |
|  |  |  |  |  |  |  | 90%(461/514) | AJ972860 | *Capnobotryella sp.* | Marble in Turkey |
| 102 | 1 | 4 | Ascomycota | Chaetothyriales | Herpotrichiellaceae | *Exophila sp.* ^#^ | 99%(554/557) | [KF823613](http://www.ncbi.nlm.nih.gov/nucleotide/57869272?report=genbank&log$=nucltop&blast_rank=1&RID=9R00Y6YM014) | *Uncultured Exophila clone*^§^ | Dead wood in Germany |
| 103 | 2 | 7 | Ascomycota | Saccharomycetales | Saccharomycetaceae | *Pichia pastoris** | 97%(368/379) | FR839630 | *Pichia pastoris*^§^ | Unreported |
| 104 | 1 | 3 | Ascomycota | Unassigned | Unassigned | *Ascomycota sp.* | 86%(407/473) | [AY679141](http://www.ncbi.nlm.nih.gov/nucleotide/22024444?report=genbank&log$=nucltop&blast_rank=1&RID=9R00Y6YM014) | *Uncultured ascomycete clone*^§^ | Unreported |
| 105 | 6 | 69 | Ascomycota | Capnodiales | Unassigned | *Capnodiales sp.* | 99%(504/506) | [KF296999](http://www.ncbi.nlm.nih.gov/nucleotide/305671346?report=genbank&log$=nucltop&blast_rank=1&RID=9R00Y6YM014) | *Uncultured fungus clone*^§^ | Arctic soil in Canada (76.23N; 119.30W) |
|  |  |  |  |  |  |  | 87%(360/414) | GQ852801 | *Teratosphaeria eucalypti* | Plant in Australia |
| 106 | 2 | 133 | Ascomycota | Hypocreales | Unassigned | *Hypocreales sp.* | 95%(514/539) | [GU817178](http://www.ncbi.nlm.nih.gov/nucleotide/307750659?report=genbank&log$=nucltop&blast_rank=1&RID=9R00Y6YM014) | *Uncultured fungus clone* | Roots in Svalbard (78.90N; 12.08E) |
|  |  |  |  |  |  |  | 89%(476/534) | GU067755 | *Pseudocosmospora vilior* | Wood stump in Finland |
| 107 | 5 | 133 | Ascomycota | Chaetothyriales | Herpotrichiellaceae | *Herpotrichiellaceae sp.* | 99%(555/556) | FJ554329 | *Uncultured Herpotrichiellaceae clone*^§^ | Forest soil in Canada |
| 108 | 5 | 92 | Ascomycota | Helotiales | Unassigned | *Helotiales sp.* | 96%(456/477) | [JQ666493](http://www.ncbi.nlm.nih.gov/nucleotide/266634419?report=genbank&log$=nucltop&blast_rank=1&RID=9R00Y6YM014) | *Uncultured soil fungus clone* | Forest soil in China (Changbai mountain) |
|  |  |  |  |  |  |  | 95%(408/428) | KC485427 | *Helotiales sp.* | Algae in Antarctica |
| 109 | 1 | 57 | Ascomycota | Venturiales | Ventruiaceae | *Ventruiaceae sp.* | 99% (534/538) | [KC965430](http://www.ncbi.nlm.nih.gov/nucleotide/387145937?report=genbank&log$=nucltop&blast_rank=1&RID=9R00Y6YM014) | *Uncultured fungus clone*^§^ | Arctic soil in USA (69.15N; 148.85W) |
|  |  |  |  |  |  |  | 90%(507/563) | EU035459 | *Venturia hystrioides* | Plant in USA |
| 110 | 3 | 39 | Ascomycota | Chaetothyriales | Unassigned | *Chaetothyriales sp.* | 90%(424/473) | [KC966293](http://www.ncbi.nlm.nih.gov/nucleotide/56181649?report=genbank&log$=nucltop&blast_rank=1&RID=9R00Y6YM014) | *Uncultured fungus clone* | Arctic soil in Canada (76.23N,119.30W) |
|  |  |  |  |  |  |  | 89%(409/460) | EU139158 | *Capronia sp.* | Endolichenic fungi in China |
| 111 | 3 | 4 | Ascomycota | Helotiales | Unassigned | *Helotiales sp.* | 94%(459/487) | [FR846473](http://www.ncbi.nlm.nih.gov/nucleotide/387966464?report=genbank&log$=nucltop&blast_rank=2&RID=9R00Y6YM014) | *Helotiales sp.* | Host litter needles in Czech |
| 112 | 3 | 77 | Ascomycota | Capnodiales | Teratosphaeriaceae | *Devriesia sp.* ^#^ | 99%(533/534) | [KC965858](http://www.ncbi.nlm.nih.gov/nucleotide/387145970?report=genbank&log$=nucltop&blast_rank=1&RID=9R00Y6YM014) | *Uncultured fungus clone*^§^ | Arctic soil in Canada (76.23N,119.30W) |
|  |  |  |  |  |  |  | 96%(361/378) | GU214635 | *Devriesia strelitziicola* | South Africa |
| 113 | 2 | 32 | Ascomycota | Chaetothyriales | Herpotrichiellaceae | *Cladophialophora sp.* ^#^ | 98%(540/552) | [KF274346](http://www.ncbi.nlm.nih.gov/nucleotide/311334607?report=genbank&log$=nucltop&blast_rank=2&RID=9R00Y6YM014) | *Uncultured fungus clone*^§^ | Wood stump in Finland |
|  |  |  |  |  |  |  | 95%(523/553) | EU035408 | *Cladophialophora humicolae* | Soil in Germany |
| 114 | 1 | 71 | Ascomycota | Helotiales | Unassigned | *Helotiales sp.* | 91%(452/497) | [KF296739](http://www.ncbi.nlm.nih.gov/nucleotide/408884343?report=genbank&log$=nucltop&blast_rank=1&RID=9R00Y6YM014) | *Uncultured fungus clone* | Arctic soil in Canada (76.23N, 119.30W) |
|  |  |  |  |  |  |  | 89%(423/475) | JX630692 | *Uncultured Tetracladium clone* | Plant root in USA |
| 115 | 2 | 3 | Basidiomycota | Agaricostilbales | Unassigned | *Agaricostilbales sp.* | 99%(233/236) | JN906031 | *Uncultured fungus clone*^§^ | Phyllosphere in France |
|  |  |  |  |  |  |  | 94%(188/200) | AF444634 | *Bensingtonia yamatoana* | Unreported |
| 116 | 2 | 183 | Ascomycota | Helotiales | Helotiaceae | *Rhizoscyphus ericae** | 98%(518/530) | [AY762620](http://www.ncbi.nlm.nih.gov/nucleotide/342674097?report=genbank&log$=nucltop&blast_rank=1&RID=9R00Y6YM014) | *Rhizoscyphus ericae*^§^ | Unreported |
| 117 | 2 | 74 | Basidiomycota | Cystofilobasidiales | Cystofilobasidiaceae | *Itersonilia sp.* ^#^ | 97%(515/532) | [AM901971](http://www.ncbi.nlm.nih.gov/nucleotide/109452380?report=genbank&log$=nucltop&blast_rank=1&RID=9R00Y6YM014) | *Uncultured basidiomycete clone*^§^ | House dust in Finland |
|  |  |  |  |  |  |  | 96%(488/506) | NR077117 | *Itersonilia perplexans* | Unreported |
| 118 | 1 | 4 | Unassigned | Unassigned | Unassigned | *Fungus sp.* | 88%(380/430) | [KF274365](http://www.ncbi.nlm.nih.gov/nucleotide/28974811?report=genbank&log$=nucltop&blast_rank=1&RID=9R00Y6YM014) | *Uncultured fungus clone* | Wood stump in Finland |
| 119 | 9 | 112 | Ascomycota | Chaetothyriales | Herpotrichiellaceae | *Herpotrichiellaceae sp.* | 98%(562/571) | [KC965393](http://www.ncbi.nlm.nih.gov/nucleotide/399572806?report=genbank&log$=nucltop&blast_rank=1&RID=9R00Y6YM014) | *Uncultured fungus clone*^§^ | Arctic soil in Canada (78.78N; 103.55W) |
|  |  |  |  |  |  |  | 93%(536/576) | EU139138 | *Rhinocladiella sp.* | Endolichenic fungi in lichen from China |
| 120 | 3 | 15 | Ascomycota | Verrucariales | Verrucariaceae | *Verrucariaceae sp.* | 98%(478/489) | KC966180 | *Uncultured fungus clone*^§^ | Arctic soil in USA (69.67N; 148.72W) |
|  |  |  |  |  |  |  | 91%(423/465) | EU559739 | *Polyblastia sp.* | Sweden |
| 121 | 3 | 5 | Ascomycota | Pleosporales | Melanommataceae | *Herpotrichia juniperi** | 98%(523/532) | [DQ420833](http://www.ncbi.nlm.nih.gov/nucleotide/194338513?report=genbank&log$=nucltop&blast_rank=1&RID=9R00Y6YM014) | *Uncultured soil fungus clone*^§^ | Soil in USA |
|  |  |  |  |  |  |  | 98%(458/465) | JX981496 | *Herpotrichia juniperi*^§^ | Plant in Poland |
| 122 | 1 | 135 | Ascomycota | Unassigned | Unassigned | *Ascomycota sp.* | 97%(381/392) | [KC965806](http://www.ncbi.nlm.nih.gov/nucleotide/307750625?report=genbank&log$=nucltop&blast_rank=1&RID=9R00Y6YM014) | *Uncultured fungus clone*^§^ | Arctic soil in Canada (76.23N; 119.30W) |
|  |  |  |  |  |  |  | 92%(381/416) | HM239990 | *Uncultured Ascomycota clone* | Grass soil in USA |
| 123 | 1 | 2 | Ascomycota | Helotiales | Helotiaceae | *Claussenomyces sp.* ^#^ | 100%(495/495) | KC966294 | *Uncultured fungus clone*^§^ | Arctic soil in USA (69.15N; 148.85W) |
|  |  |  |  |  |  |  | 96%(497/519) | HQ533008 | *Claussenomyces sp.* | New Zealand |
| 124 | 5 | 103 | Ascomycota | Helotiales | Hyaloscyphaceae | *Hyaloscyphaceae sp.* | 94%(512/544) | [DQ227258](http://www.ncbi.nlm.nih.gov/nucleotide/305671346?report=genbank&log$=nucltop&blast_rank=1&RID=9R00Y6YM014) | *Hyphodiscus hymeniophilus* | Unreported |
| 125 | 3 | 41 | Ascomycota | Helotiales | Helotiaceae | *Godronia cassandrae** | 99% (510/515) | [KC595271](http://www.ncbi.nlm.nih.gov/nucleotide/307750642?report=genbank&log$=nucltop&blast_rank=1&RID=A1G7PDYP01R) | *Godronia cassandrae*^§^ | Germany |
| 126 | 1 | 3 | Basidiomycota | Thelephorales | Thelephoraceae | *Tomentella sp.* ^#^ | 97% (463/479) | [EU625883](http://www.ncbi.nlm.nih.gov/nucleotide/371766716?report=genbank&log$=nucltop&blast_rank=1&RID=A1G7PDYP01R) | *Uncultured Tomentella clone*^§^ | Mixed hardwood forest in USA |
| 127 | 2 | 6 | Ascomycota | Lecideales | Lecideaceae | *Lecideaceae sp.* | 91%(410/453) | [DQ534472](http://www.ncbi.nlm.nih.gov/nucleotide/315270717?report=genbank&log$=nucltop&blast_rank=1&RID=A1G7PDYP01R) | *Lecidea cancriformis* | Unreported |
| 128 | 3 | 13 | Ascomycota | Helotiales | Helotiaceae | *Hymenoscyphus sp.* ^#^ | 99%(490/493) | [KC966005](http://www.ncbi.nlm.nih.gov/nucleotide/315270203?report=genbank&log$=nucltop&blast_rank=1&RID=9R00Y6YM014) | *Uncultured fungus clone*^§^ | Arctic soil in Canada (78.78N; 103.55W) |
|  |  |  |  |  |  |  | 96%(493/515) | AB705232 | *Hymenoscyphus sp.* | Japan |
| 129 | 1 | 8 | Ascomycota | Lecanorales | Unassigned | *Lecanorales sp.* | 97%(233/240) | KC966341 | *Uncultured fungus clone*^§^ | Arctic soil in Canada (76.23N; 119.30W) |
|  |  |  |  |  |  |  | 88%(233/264) | DQ534473 | *Lepraria membranacea* | Unreported |
| 130 | 1 | 12 | Basidiomycota | Sebacinales | Sebacinaceae | *Sebacina sp.* ^#^ | 98%(466/476) | EU909222 | *Uncultured Sebacina clone*^§^ | Liverwort (Riccardia latifrons) |
| 131 | 5 | 142 | Ascomycota | Pleosporales | Pleosporaceae | *Alternaria sp.* ^#^ | 100%(514/514) | [KC920885](http://www.ncbi.nlm.nih.gov/nucleotide/6273855?report=genbank&log$=nucltop&blast_rank=1&RID=9R00Y6YM014) | *Uncultured Alternaria clone*^§^ | Unreported |
| 132 | 2 | 142 | Ascomycota | Helotiales | Unassigned | *Helotiales sp.* | 99%(494/499) | [KC965311](http://www.ncbi.nlm.nih.gov/nucleotide/344333052?report=genbank&log$=nucltop&blast_rank=1&RID=9R00Y6YM014) | *Uncultured fungus clone*^§^ | Arctic soil in Canada (73.22N; 119.56W) |
|  |  |  |  |  |  |  | 94%(493/522) | GU327472 | *Uncultured Tetracladium clone* | Mycorrhizal seedling in Czech |
| 133 | 1 | 7 | Ascomycota | Unassigned | Unassigned | *Ascomycota sp.* | 99%(537/539) | [KC966353](http://www.ncbi.nlm.nih.gov/nucleotide/372293536?report=genbank&log$=nucltop&blast_rank=1&RID=9R00Y6YM014) | *Uncultured fungus clone*^§^ | Arctic soil in Canada (76.23N,119.30W) |
|  |  |  |  |  |  |  | 93%(522/564) | FN555433 | *Uncultured Ascomycota clone* | Liverwort in Antarctica |
| 134 | 2 | 17 | Unassigned | Unassigned | Unassigned | *Fungus sp.* | 99%(528/529) | [KC965283](http://www.ncbi.nlm.nih.gov/nucleotide/289190102?report=genbank&log$=nucltop&blast_rank=1&RID=9R00Y6YM014) | *Uncultured fungus clone*^§^ | Arctic soil in Canada (76.23N,119.30W) |
| 135 | 4 | 15 | Unassigned | Unassigned | Unassigned | *Fungus sp.* | 94%(449/476) | [JQ666656](http://www.ncbi.nlm.nih.gov/nucleotide/344332920?report=genbank&log$=nucltop&blast_rank=1&RID=9R00Y6YM014) | *Uncultured fungus clone* | Forest soil in China (Changbai mountain) |
| 136 | 10 | 534 | Ascomycota | Helotiales | Helotiaceae | *Helotiaceae sp.* | 98%(426/434) | [KF274099](http://www.ncbi.nlm.nih.gov/nucleotide/326319947?report=genbank&log$=nucltop&blast_rank=1&RID=9R00Y6YM014) | *Uncultured fungus clone*^§^ | Wood stump in Finland |
|  |  |  |  |  |  |  | 92%(394/426) | HQ533008 | *Claussenomyces sp.* | New Zealand |
| 137 | 2 | 9 | Basidiomycota | Sporidiobolas | Unassigned | *Sporidiobolas sp.* | 88%(446/507) | [FN548148](http://www.ncbi.nlm.nih.gov/nucleotide/374638336?report=genbank&log$=nucltop&blast_rank=1&RID=9R00Y6YM014) | *Rhodotorula sp.* | Living leaves in Germany |
| 138 | 3 | 20 | Ascomycota | Xylariales | Amphisphaeriaceae | *Seimatosporium sp.* | 95%(509/534) | [JN871207](http://www.ncbi.nlm.nih.gov/nucleotide/3169884?report=genbank&log$=nucltop&blast_rank=1&RID=9R00Y6YM014) | *Seimatosporium walkeri* | Australia |
| 139 | 3 | 254 | Ascomycota | Chaetothyriales | Herpotrichiellaceae | *Herpotrichiellaceae sp.* | 97%(540/558) | [FJ554329](http://www.ncbi.nlm.nih.gov/nucleotide/266634419?report=genbank&log$=nucltop&blast_rank=1&RID=9R00Y6YM014) | *Uncultured Herpotrichiellaceae clone*^§^ | Forest soil in Canada |
| 140 | 2 | 134 | Ascomycota | Unassigned | Unassigned | *Ascomycota sp.* | 89%(403/451) | [KF274343](http://www.ncbi.nlm.nih.gov/nucleotide/399572806?report=genbank&log$=nucltop&blast_rank=1&RID=9R00Y6YM014) | *Uncultured fungus clone* | Wood stump in Finland |
|  |  |  |  |  |  |  | 87%(379/435) | HM239991 | *Uncultured Ascomycota clone* | Grass soil in USA |
| 141 | 1 | 6 | Ascomycota | Unassigned | Unassigned | *Ascomycota sp.* | 97%(494/510) | [KC966233](http://www.ncbi.nlm.nih.gov/nucleotide/307750623?report=genbank&log$=nucltop&blast_rank=1&RID=9R00Y6YM014) | *Uncultured fungus clone*^§^ | Arctic soil in Canada (78.78N,103.55W) |
|  |  |  |  |  |  |  | 81%(376/466) | NR119924 | *Placiopsis perrugosa* | Unreported |
| 142 | 2 | 22 | Ascomycota | Lecanorales | Stereocaulaceae | *Stereocaulaceae sp.* | 99%(497/501) | KC966341 | *Uncultured fungus clone*^§^ | Arctic soil in Canada (76.23N,119.30W) |
|  |  |  |  |  |  |  | 92%(475/516) | KF682450 | *Lepraria jackii* | Finland |
| 143 | 1 | 14 | Ascomycota | Acarosporales | Unassigned | *Acarosporales sp.* | 86%(384/446) | DQ534451 | *Acarospora austroshetlandica* | Antarctica |
| 144 | 2 | 4 | Basidiomycota | Unassigned | Unassigned | *Basidiomycota sp.* | 91%(243/268) | [JQ768935](http://www.ncbi.nlm.nih.gov/nucleotide/315270717?report=genbank&log$=nucltop&blast_rank=1&RID=9R00Y6YM014) | *Basidiomycota sp.* | Glacier surface snow in Tibet |
| 145 | 3 | 103 | Ascomycota | Helotiales | Unassigned | *Helotiales sp.* | 98%(489/501) | [KC965147](http://www.ncbi.nlm.nih.gov/nucleotide/298357253?report=genbank&log$=nucltop&blast_rank=7&RID=9R00Y6YM014) | *Uncultured fungus clone*^§^ | Arctic soil in Canada (73.22N; 119.56W) |
|  |  |  |  |  |  |  | 95%(499/524) | JN859275 | *Helotiales sp.* | Plant root in Hungary |
| 146 | 4 | 84 | Unassigned | Unassigned | Unassigned | *Fungus sp.* | 90%(474/526) | [HM069353](http://www.ncbi.nlm.nih.gov/nucleotide/307750650?report=genbank&log$=nucltop&blast_rank=1&RID=9R00Y6YM014) | *Uncultured fungus clone* | Pine forest soil |
| 147 | 2 | 41 | Ascomycota | Lecanorales | Ramalinaceae | *Biatora subduples** | 98%(488/496) | AJ247540 | *Biatora subduples*^§^ | Unreported |
| 148 | 4 | 9 | Ascomycota | Capnodiales | Unassigned | *Elasticomyces elasticus** | 99%(521/525) | [KC966349](http://www.ncbi.nlm.nih.gov/nucleotide/344333334?report=genbank&log$=nucltop&blast_rank=1&RID=9R00Y6YM014) | *Uncultured fungus clone*^§^ | Arctic soil in Canada (76.23N; 119.30W) |
|  |  |  |  |  |  |  | 98%(513/521) | FJ415476 | *Elasticomyces elasticus*^§^ | Antarctica |
| 149 | 2 | 52 | Ascomycota | Unassigned | Unassigned | *Ascomycota sp.* | 91%(460/505) | [KC965473](http://www.ncbi.nlm.nih.gov/nucleotide/307750624?report=genbank&log$=nucltop&blast_rank=1&RID=9R00Y6YM014) | *Uncultured fungus clone* | Arctic soil in Canada (76.23N,119.30W) |
|  |  |  |  |  |  |  | 86%(434/504) | HM239886 | *Uncultured Ascomycota clone* | Grass soil in USA |
| 150 | 1 | 11 | Unassigned | Unassigned | Unassigned | *Fungus sp.* | 87%(418/481) | [KF274185](http://www.ncbi.nlm.nih.gov/nucleotide/387966464?report=genbank&log$=nucltop&blast_rank=1&RID=9R00Y6YM014) | *Uncultured fungus clone* | Wood stump in Finland |
| 151 | 2 | 14 | Ascomycota | Helotiales | Leotiaceae | *Alatospora flagellata** | 98%(503/511) | [KC834041](http://www.ncbi.nlm.nih.gov/nucleotide/307750639?report=genbank&log$=nucltop&blast_rank=1&RID=9R00Y6YM014) | *Alatospora flagellata*^§^ | Stream |
| 152 | 2 | 29 | Ascomycota | Lecanorales | Lecideaceae | *Lecideaceae sp.* | 94%(482/511) | HQ605940 | *Porpidia macrocarpa* | Turkey |
| 153 | 5 | 11 | Ascomycota | Capnodiales | Cladosporiaceae | *Cladosporium cf. tenuissimum** | 100% (546/546) | [HM148219](http://www.ncbi.nlm.nih.gov/nucleotide/307750639?report=genbank&log$=nucltop&blast_rank=1&RID=9R00Y6YM014) | *Cladosporium cf. tenuissimum*^§^ | Forest soil in China |
| 154 | 4 | 20 | Ascomycota | Helotiales | Unassigned | *Tetracladium furcatum** | 98%(523/532) | [AM901833](http://www.ncbi.nlm.nih.gov/nucleotide/6273855?report=genbank&log$=nucltop&blast_rank=1&RID=9R00Y6YM014) | *Uncultured ascomycete clone*^§^ | House dust in Finland |
|  |  |  |  |  |  |  | 97%(518/532) | EU883432 | *Tetracladium furcatum*^§^ | Unreported |
| 155 | 1 | 12 | Ascomycota | Unassigned | Unassigned | *Ascomycota sp.* | 91%(454/498) | [KC965473](http://www.ncbi.nlm.nih.gov/nucleotide/311334607?report=genbank&log$=nucltop&blast_rank=3&RID=9R00Y6YM014) | *Uncultured fungus clone* | Arctic soil in Canada (73.22N; 119.56W) |
|  |  |  |  |  |  |  | 85%(422/492) | FJ903295 | *Sarea difformis* | Stump of Picea abies in Latvia |
| 156 | 6 | 226 | Basidiomycota | Unassigned | Unassigned | *Basidiomycota sp.* | 99%(500/501) | [KF274145](http://www.ncbi.nlm.nih.gov/nucleotide/209870738?report=genbank&log$=nucltop&blast_rank=1&RID=9R00Y6YM014) | *Uncultured fungus clone*^§^ | Wood stump in Finland |
|  |  |  |  |  |  |  | 91%(471/520) | AM901836 | *Uncultured basidiomycete lcone* | House dust in Finland |
| 157 | 5 | 23 | Ascomycota | Capnodiales | Teratosphaeriaceae | *Teratosphaeriaceae sp.* | 90%(407/454) | [EU490051](http://www.ncbi.nlm.nih.gov/nucleotide/344333334?report=genbank&log$=nucltop&blast_rank=1&RID=9R00Y6YM014) | *Uncultured ascomycete clone* | Savanna soil in USA |
|  |  |  |  |  |  |  | 90%(406/453) | JF499833 | *Catenulostroma hermanusense* | Leaf bracts in South Africa |
| 158 | 1 | 2 | Ascomycota | Hypocreales | Unassigned | *Acremonium sp.* ^#^ | 98%(511/519) | [JQ410055](http://www.ncbi.nlm.nih.gov/nucleotide/99866600?report=genbank&log$=nucltop&blast_rank=1&RID=9R00Y6YM014) | *Uncultured fungus clone*^§^ | Soil in China (Shanxi) |
|  |  |  |  |  |  |  | 98%(508/519) | EF577237 | *Acremonium sp.* ^§^ | Soil in China |
| 159 | 2 | 51 | Ascomycota | Helotiales | Unassigned | *Helotiales sp.* | 99%(498/502) | [KF296765](http://www.ncbi.nlm.nih.gov/nucleotide/125634642?report=genbank&log$=nucltop&blast_rank=1&RID=9R00Y6YM014) | *Uncultured fungus clone*^§^ | Arctic soil in Canada (76.23N; 119.30W) |
|  |  |  |  |  |  |  | 94%(498/527) | FJ378851 | *Uncultured Helotiales clone* | Ectomycorrhiza in Himalaya |
| 160 | 3 | 8 | Ascomycota | Microascales | Halosphaeriaceae | *Monodictys arctica** | 99% (503/510) | [GU817175](http://www.ncbi.nlm.nih.gov/nucleotide/387966523?report=genbank&log$=nucltop&blast_rank=1&RID=9R00Y6YM014) | *Uncultured fungus clone*^§^ | Plant root in Svalbard |
|  |  |  |  |  |  |  | 99%(500/507) | EU686522 | *Monodictys arctica*^§^ | Plant root in High Arctic Canada |
| 161 | 8 | 22 | Ascomycota | Pleosporales | Sporormiaceae | *Preussia sp.* ^#^ | 99%(516/517) | [KC965263](http://www.ncbi.nlm.nih.gov/nucleotide/268636567?report=genbank&log$=nucltop&blast_rank=1&RID=9R00Y6YM014) | *Uncultured fungus clone*^§^ | Arctic soil in USA (70.31N; 147.99W) |
|  |  |  |  |  |  |  | 99%(510/512) | HQ602666 | *Preussia sp.* ^§^ | surface sterilized needle tissue in USA |
| 162 | 2 | 3 | Ascomycota | Unassigned | Unassigned | *Ascomycota sp.* | 98%(265/270) | KF297149 | *Uncultured fungus clone*^§^ | Arctic soil in Canada (76.23N;119.30W) |
|  |  |  |  |  |  |  | 83%(250/303) | DQ534452 | *Bryoria fuscescens* | Antarctica |
| 163 | 2 | 6 | Ascomycota | Rhytismatales | Unassigned | *Rhytismatales sp.* | 86%(503/503) | AB714536 | *Rhytisma polare* | Norway: Spitsbergen, Ny-Alesund |
| 164 | 1 | 4 | Ascomycota | Capnodiales | Unassigned | *Capnodiales sp.* | 92%(331/359) | GU931741 | *Uncultured Capnodiales clone* | House dust in Canada |
| 165 | 1 | 5 | Ascomycota | Helotiales | Unassigned | *Helotiales sp.* | 93%(468/502) | [KC966220](http://www.ncbi.nlm.nih.gov/nucleotide/380003900?report=genbank&log$=nucltop&blast_rank=1&RID=9R00Y6YM014) | *Uncultured fungus clone* | Arctic soil in Canada (78.78N; 103.55W) |
|  |  |  |  |  |  |  | 86%(385/448) | AB705255 | *Lambertella sp.* | Unreported |
| 166 | 1 | 40 | Basidiomycota | Sebacinales | Sebacinaceae | *Sebacina sp.* ^#^ | 99%(490/492) | [GQ907078](http://www.ncbi.nlm.nih.gov/nucleotide/19913082?report=genbank&log$=nucltop&blast_rank=1&RID=9R00Y6YM014) | *Uncultured Sebacina clone*^§^ | Liverwort in Switzerland |
| 167 | 1 | 113 | Ascomycota | Helotiales | Helotiaceae | *Claussenomyces sp.* ^#^ | 98%(514/522) | [GU174358](http://www.ncbi.nlm.nih.gov/nucleotide/357934273?report=genbank&log$=nucltop&blast_rank=2&RID=9R00Y6YM014) | *Uncultured fungus clone*^§^ | Forest floor in USA (43.66N; 86.14W) |
|  |  |  |  |  |  |  | 95%(500/527) | HQ533008 | *Claussenomyces sp.* | New Zealand |
| 168 | 18 | 1276 | Ascomycota | Helotiales | Leotiaceae | *Gorgomyces sp.* ^#^ | 99%(513/514) | [KC966006](http://www.ncbi.nlm.nih.gov/nucleotide/305671348?report=genbank&log$=nucltop&blast_rank=1&RID=A1G7PDYP01R) | *Uncultured fungus clone*^§^ | Arctic soil in Canada (78.78N, 103.55W) |
|  |  |  |  |  |  |  | 95%(494/519) | KC834057 | *Gorgomyces honrubiae* | Stream |
| 169 | 2 | 3 | Unassigned | Unassigned | Unassigned | *Fungus sp.* | 100%(506/506) | [KC965500](http://www.ncbi.nlm.nih.gov/nucleotide/408882798?report=genbank&log$=nucltop&blast_rank=4&RID=A1G7PDYP01R) | *Uncultured fungus clone*^§^ | Arctic soil in USA (69.67N; 148.72W) |
| 170 | 2 | 319 | Ascomycota | Helotiales | Unassigned | *Helotiales sp.* | 89%(406/454) | HQ631037 | *Scytalidium sp.* | Unreported |
| 171 | 1 | 21 | Ascomycota | Helotiales | Helotiaceae | *Helotiaceae sp.* | 91%(440/484) | [HM030573](http://www.ncbi.nlm.nih.gov/nucleotide/117574028?report=genbank&log$=nucltop&blast_rank=1&RID=9R00Y6YM014) | *Uncultured fungus clone* | Plant root soil in USA |
|  |  |  |  |  |  |  | 90%(435/481) | FJ440900 | *Uncultured Helotiaceae clone* | Plant root in USA |
| 172 | 2 | 96 | Ascomycota | Lecanorales | Unassigned | *Lecidea ementiens** | 97%(494/508) | [KF297029](http://www.ncbi.nlm.nih.gov/nucleotide/307750641?report=genbank&log$=nucltop&blast_rank=1&RID=9R00Y6YM014) | *Uncultured fungus clone*^§^ | Arctic soil in Canada (76.23N; 119.30W) |
|  |  |  |  |  |  |  | 97%(449/463) | KF650962 | *Lecidea ementiens*^§^ | Unreported |
| 173 | 2 | 34 | Ascomycota | Baeomycetales | Unassigned | *Baeomycetales sp.* | 91%(448/492) | [KC965473](http://www.ncbi.nlm.nih.gov/nucleotide/209412925?report=genbank&log$=nucltop&blast_rank=1&RID=9R00Y6YM014) | *Uncultrued fungus clone* | Arctic soil in Canada (73.22N; 119.56W) |
|  |  |  |  |  |  |  | 89%(379/426) | FJ903295 | *Sarea difformis* | Stump in Latvia |
| 174 | 6 | 44 | Basidiomycota | Tremellales | Syzygosporaceae | *Syzygosporaceae sp.* | 100%(481/481) | [KF296901](http://www.ncbi.nlm.nih.gov/nucleotide/387966434?report=genbank&log$=nucltop&blast_rank=1&RID=A1G7PDYP01R) | *Uncultured fungus clone*^§^ | Arctic soil in Canada (76.23N;119.30W) |
|  |  |  |  |  |  |  | 92%(334/365) | JN053505 | *Syzygospora bachmannii* | Unreported |
| 175 | 6 | 41 | Ascomycota | Helotiales | Helotiaceae | *Helotiaceae sp.* | 98%(495/507) | [HM069356](http://www.ncbi.nlm.nih.gov/nucleotide/1857514?report=genbank&log$=nucltop&blast_rank=2&RID=9R00Y6YM014) | *Uncultured fungus clone*^§^ | Pine forest soil |
|  |  |  |  |  |  |  | 94%(465/495) | KF359569 | *Hymenoscyphus monotropae* | Plant root in USA |
| 176 | 4 | 14 | Unassigned | Unassigned | Unassigned | *Fungus sp.* | 94%(520/551) | [KC965541](http://www.ncbi.nlm.nih.gov/nucleotide/289190130?report=genbank&log$=nucltop&blast_rank=1&RID=9R00Y6YM014) | *Uncultured fungus clone* | Arctic soil in Canada (76.23N; 119.30W) |
| 177 | 1 | 16 | Ascomycota | Capnodiales | Unassigned | *Capnodiales sp.* | 99% (474/476) | [JQ759479](http://www.ncbi.nlm.nih.gov/nucleotide/317383377?report=genbank&log$=nucltop&blast_rank=1&RID=9R00Y6YM014) | *Dothideomycetes sp.* ^§^ | Plant in Canada (64.50N; 165.40W) |
|  |  |  |  |  |  |  | 94%(503/536) | JF499843 | *Penidiella ellipsoidea* | Plant in South Africa |
| 178 | 1 | 23 | Basidiomycota | Platygloeales | Unassigned | *Platygloeales sp.* | 95%(454/479) | [KF297051](http://www.ncbi.nlm.nih.gov/nucleotide/57869255?report=genbank&log$=nucltop&blast_rank=1&RID=9R00Y6YM014) | *Uncultured fungus clone* | Arctic soil in Canada (78.78N; 103.55W) |
|  |  |  |  |  |  |  | 91%(394/432) | JX852332 | *Eocronartium sp.* | Moss in Antaractica |
| 179 | 1 | 134 | Ascomycota | Hypocreales | Clavicipitaceae | *Pochonia sp.* ^#^ | 99%(464/467) | [KF741827](http://www.ncbi.nlm.nih.gov/nucleotide/315270612?report=genbank&log$=nucltop&blast_rank=1&RID=9R00Y6YM014) | *Pochonia sp.*^§^ | Soil in Svalbard (Arctic, Sorgfjorden) |
| 180 | 1 | 3 | Basidiomycota | Unassigned | Unassigned | *Basidiomycota sp.* | 90%(385/429) | [HM030585](http://www.ncbi.nlm.nih.gov/nucleotide/387966503?report=genbank&log$=nucltop&blast_rank=1&RID=9R00Y6YM014) | *Uncultured fungus clone* | Soil in USA |
|  |  |  |  |  |  |  | 85%(410/480) | AY605706 | *Basidiomycete sp.* | Unreported |
| 181 | 3 | 64 | Basidiomycota | Tremellales | Unassigned | *Tremellales sp.* | 99%(493/497) | KC966080 | *Uncultured fungus clone*^§^ | Arctic soil in Canada (76.23N; 119.30W) |
|  |  |  |  |  |  |  | 98%(442/450) | GU327507 | *Uncultured Tremellales clone*^§^ | Mycorrhizal seedling in Cezch |
| 182 | 2 | 3 | Ascomycota | Helotiales | Leotiaceae | *Gorgomyces sp.* ^#^ | 99%(458/459) | [KC966006](http://www.ncbi.nlm.nih.gov/nucleotide/284504032?report=genbank&log$=nucltop&blast_rank=1&RID=9R00Y6YM014) | *Uncultured fungus clone*^§^ | Arctic soil in Canada (78.78N; 103.55W) |
|  |  |  |  |  |  |  | 95%(443/464) | KC834057 | *Gorgomyces honrubiae* | Stream |
| 183 | 1 | 10 | Basidiomycota | Sebacinales | Sebacinaceae | *Sebacina sp.* ^#^ | 99%(500/506) | AB669636 | *Uncultured mycorrhizal fungus*^§^ | Mycorrhizal root tip in forest soil |
|  |  |  |  |  |  |  | 98%(472/481) | [JX844772](http://www.ncbi.nlm.nih.gov/nucleotide/13936258?report=genbank&log$=nucltop&blast_rank=1&RID=9R00Y6YM014) | *Uncultured Sebacina clone*^§^ | Mycorrhizal root tip |
| 184 | 1 | 78 | Ascomycota | Unassigned | Unassigned | *Ascomycota sp.* | 98%(536/546) | KC965530 | *Uncultured fungus clone*^§^ | Arctic soil in Canada (78.78N; 103.55W) |
|  |  |  |  |  |  |  | 86%(502/583) | AM902024 | *Uncultured ascomycete clone* | House dust in Finland |
| 185 | 5 | 22 | Ascomycota | Helotiales | Unassigned | *Helotiales sp.* | 99%(493/494) | [JX852326](http://www.ncbi.nlm.nih.gov/nucleotide/305671291?report=genbank&log$=nucltop&blast_rank=1&RID=9R00Y6YM014) | *Helotiales sp.* ^§^ | Antarctica liverwort |
| 186 | 5 | 32 | Ascomycota | Chaetothyriales | Herpotrichiellaceae | *Herpotrichiellaceae sp.* | 98%(562/571) | [FJ554179](http://www.ncbi.nlm.nih.gov/nucleotide/307750672?report=genbank&log$=nucltop&blast_rank=1&RID=9R00Y6YM014) | *Uncultured Herpotrichiellaceae clone*^§^ | Forest soil in Canada |
| 187 | 4 | 93 | Ascomycota | Lecanorales | Porpidiaceae | *Biatora sp.* ^#^ | 98%(506/514) | KC965546 | *Uncultured fungus clone*^§^ | Arctic soil in Canada (76.23N; 119.30W) |
|  |  |  |  |  |  |  | 95%(482/510) | AJ247561 | *Biatora tetramera* | Finland |
| 188 | 1 | 2657 | Ascomycota | Helotiales | Unassigned | *Helotiales sp.* | 96%(492/515) | [KF274408](http://www.ncbi.nlm.nih.gov/nucleotide/307750659?report=genbank&log$=nucltop&blast_rank=1&RID=9R00Y6YM014) | *Uncultured fungus clone* | Wood stump in Finland |
|  |  |  |  |  |  |  | 95%(432/457) | EU726289 | *Uncultured Helotiales clone* | Plant in USA |
| 189 | 5 | 66 | Ascomycota | Unassigned | Unassigned | *Ascomycota sp.* | 98%(441/448) | KC965719 | *Uncultured fungus clone*^§^ | Arctic soil in Canada (78.78N; 103.55W) |
|  |  |  |  |  |  |  | 77%(375/430) | EF373563 | *Ascomycete sp.* | Tuckermannopsis chlorophylla |
| 190 | 3 | 13 | Basidiomycota | Sebacinales | Sebacinaceae | *Sebacina sp.* ^#^ | 99%(485/486) | GQ907091 | *Uncultured Sebacina clone*^§^ | Liverwort in Scotland |
| 191 | 1 | 11 | Ascomycota | Capnodiales | Teratosphaeriaceae | *Teratosphaeriaceae sp.* | 99%(415/419) | [KC966025](http://www.ncbi.nlm.nih.gov/nucleotide/289190145?report=genbank&log$=nucltop&blast_rank=1&RID=9R00Y6YM014) | *Uncultured fungus clone*^§^ | Arctic soil in Canada (76.23N; 119.30W) |
|  |  |  |  |  |  |  | 94%(412/438) | EU707864 | *Teratosphaeria jonkershoekensis* | Plant in South Africa |
| 192 | 1 | 7 | Ascomycota | Unassigned | Unassigned | *Ascomycota sp.* | 85%(457/542) | DQ534483 | *Rhizocarpon nidificum* | Unreported |
| 193 | 3 | 12 | Ascomycota | Unassigned | Unassigned | *Oidiodendron sp.* ^#^ | 97%(480/493) | [JX270413](http://www.ncbi.nlm.nih.gov/nucleotide/323320622?report=genbank&log$=nucltop&blast_rank=4&RID=9R00Y6YM014) | *Oidiodendron sp.* ^§^ | Soil in USA |
| 194 | 1 | 8 | Ascomycota | Unassigned | Unassigned | *Ascomycota sp.* | 87%(403/464) | HM161962 | *Uncultured Ascomycota clone* | Grass roots in USA |
| 195 | 2 | 6 | Ascomycota | Unassigned | Unassigned | *Ascomycota sp.* | 91%(454/498) | JF519577 | *Uncultured Pezizomycotina clone* | Forest soil in Austria |
| 196 | 6 | 380 | Ascomycota | Chaetothyriales | Unassigned | *Chaetothyriales sp.* | 99%(534/537) | KC966354 | *Uncultured fungus clone*^§^ | Arctic soil in Canada (76.23N; 119.30W) |
|  |  |  |  |  |  |  | 89%(474/530) | JQ272383 | *Herpotrichiellaceae sp.* | Plant root in USA |
| 197 | 2 | 6 | Ascomycota | Unassigned | Unassigned | *Ascomycota sp.* | 95%(420/441) | [KF297065](http://www.ncbi.nlm.nih.gov/nucleotide/290575783?report=genbank&log$=nucltop&blast_rank=1&RID=9R00Y6YM014) | *Uncultured fungus clone* | Arctic soil in Canada (76.23N; 119.30W) |
|  |  |  |  |  |  |  | 89%(416/465) | FN555433 | *Uncultured Ascomycota clone* | Liverwort in Antarctica |
| 198 | 1 | 12 | Ascomycota | Unassigned | Unassigned | *Ascomycota sp.* | 84%(353/418) | [FJ493196](http://www.ncbi.nlm.nih.gov/nucleotide/95106173?report=genbank&log$=nucltop&blast_rank=1&RID=9R00Y6YM014) | *Teratosphaeria profusa* | Unreported |
| 199 | 1 | 6 | Basidiomycota | Unassigned | Unassigned | *Basidiomycota sp.* | 88%(428/484) | [HQ850144](http://www.ncbi.nlm.nih.gov/nucleotide/307750641?report=genbank&log$=nucltop&blast_rank=1&RID=9R00Y6YM014) | *Uncultured fungus clone* | Plant root in China |
|  |  |  |  |  |  |  | 87%(410/469) | FJ553957 | *Uncultured Agaricomycetes clone* | Forest soil in Canada |
| 200 | 2 | 8 | Ascomycota | Helotiales | Helotaiceae | *Tricladium angulatum** | 99%(407/408) | [KC965406](http://www.ncbi.nlm.nih.gov/nucleotide/156865887?report=genbank&log$=nucltop&blast_rank=1&RID=9R00Y6YM014) | *Uncultured fungus clone*^§^ | Arctic soil in Canada (76.23N; 119.30W) |
|  |  |  |  |  |  |  | 97%(393/405) | AY204609 | *Tricladium angulatum*^§^ | Unreported |
| 201 | 2 | 22 | Ascomycota | Unassigned | Unassigned | *Ascomycota sp.* | 98%(405/415) | [KC965800](http://www.ncbi.nlm.nih.gov/nucleotide/6273855?report=genbank&log$=nucltop&blast_rank=1&RID=9R00Y6YM014) | *Uncultured fungus clone*^§^ | Arctic soil in Canada (76.23N; 119.30W) |
|  |  |  |  |  |  |  | 86%(457/533) | EU365847 | *Caloplaca thracopontica* | Unreported |
| 202 | 3 | 19 | Ascomycota | Capnodiales | Unassigned | *Capnodiales sp.* | 87%(338/389) | KC753419 | *Uncultured Zymiseptoria clone* | Wheat in Sweden |
| 203 | 1 | 2 | Ascomycota | Lecanorales | Unassigned | *Lecanorales sp.* | 88%(516/587) | DQ534484 | *Rhizoplaca aspidophora* | Unreported |
| 204 | 1 | 7 | Unassigned | Unassigned | Unassigned | *Fungus sp.* | 95%(297/314) | KF274408 | *Uncultured fungus clone* | Wood stump in Finland |
| 205 | 1 | 99 | Ascomycota | Helotiales | Unassigned | *Infundichalara microchona** | 99%(494/500) | [KF359590](http://www.ncbi.nlm.nih.gov/nucleotide/57869254?report=genbank&log$=nucltop&blast_rank=1&RID=9R00Y6YM014) | *Infundichalara microchona*^§^ | Plant root in USA |
| 206 | 1 | 6 | Ascomycota | Helotiales | Leotiaceae | *Alatospora acuminata** | 99%(487/491) | [AY204587](http://www.ncbi.nlm.nih.gov/nucleotide/307750639?report=genbank&log$=nucltop&blast_rank=1&RID=9R00Y6YM014) | *Alatospora acuminata*^§^ | Unreported |
| 207 | 2 | 12 | Ascomycota | Helotiales | Leotiaceae | *Alatospora sp.* ^#^ | 97%(467/483) | JF519259 | *Uncultured Alatospora clone*^§^ | Plant in Austria |
| 208 | 1 | 24 | Ascomycota | Lecanorales | Unassigned | *Lecanorales sp.* | 88%(465/530) | [EU075538](http://www.ncbi.nlm.nih.gov/nucleotide/307750688?report=genbank&log$=nucltop&blast_rank=1&RID=9R00Y6YM014) | *Miriquidica garovaqlii* | Unreported |
| 209 | 1 | 34 | Ascomycota | Lecanorales | Unassigned | *Lecanorales sp.* | 99%(528/530) | [KC965389](http://www.ncbi.nlm.nih.gov/nucleotide/124441653?report=genbank&log$=nucltop&blast_rank=1&RID=9R00Y6YM014) | *Uncultured fungus clone*^§^ | Arctic soil in USA (70.31N; 147.99W) |
|  |  |  |  |  |  |  | 90%(504/558) | DQ534467 | *Lecania brialmontii* | Antarctica |
| 210 | 5 | 1163 | Ascomycota | Helotiales | Hyaloscyphaceae | *Hyphodiscus sp.* ^#^ | 100%(500/500) | [JX852321](http://www.ncbi.nlm.nih.gov/nucleotide/99866600?report=genbank&log$=nucltop&blast_rank=1&RID=9R00Y6YM014) | *Hyphodiscus sp.* ^§^ | Antarctica moss |
| 211 | 2 | 38 | Ascomycota | Unassigned | Unassigned | *Ascomycota sp.* | 98%(486/494) | [KC965903](http://www.ncbi.nlm.nih.gov/nucleotide/55783691?report=genbank&log$=nucltop&blast_rank=1&RID=9R00Y6YM014) | *Uncultured fungus clone*^§^ | Arctic soil in Canada (78.78N; 103.55W) |
|  |  |  |  |  |  |  | 84%(427/506) | AF096215 | *Umbilicaria crustulosa* | Unreported |
| 212 | 4 | 14 | Basidiomycota | Sebacinales | Sebacinaceae | *Sebacina sp.* | 96%(480/499) | JQ420983 | *Uncultured Sebacina clone* | Plant |
| 213 | 1 | 8 | Unassigned | Unassigned | Unassigned | *Fungus sp.* | 96% (456/474) | [KC966027](http://www.ncbi.nlm.nih.gov/nucleotide/307750518?report=genbank&log$=nucltop&blast_rank=1&RID=9R00Y6YM014) | *Uncultured fungus clone* | Arctic soil in Canada (76.23N; 119.30W) |
| 214 | 1 | 3 | Ascomycota | Helotiales | Unassigned | *Helotiales sp.* | 86%(485/562) | [KJ559541](http://www.ncbi.nlm.nih.gov/nucleotide/56181649?report=genbank&log$=nucltop&blast_rank=1&RID=9R00Y6YM014) | *Unquiculariopsis lettaui* | Plant in USA |
| 215 | 1 | 2 | Ascomycota | Helotiales | Leotiaceae | *Gorgomyces honrubiae** | 98%(488/500) | [KC834057](http://www.ncbi.nlm.nih.gov/nucleotide/266634419?report=genbank&log$=nucltop&blast_rank=1&RID=9R00Y6YM014) | *Gorgomyces honrubiae*^§^ | Stream |
| 216 | 1 | 1176 | Ascomycota | Helotiales | Unassigned | *Helotiales sp.* | 85%(449/528) | AF141160 | *Dermea padi* | Unreported |
| 217 | 2 | 16 | Ascomycota | Helotiales | Unassigned | *Tetracladium sp.* ^#^ | 99%(571/519) | [GU327472](http://www.ncbi.nlm.nih.gov/nucleotide/312434590?report=genbank&log$=nucltop&blast_rank=3&RID=9R00Y6YM014) | *Uncultured Tetracladium clone*^§^ | Mycorrhizal seedling in Czech |
| 218 | 1 | 12 | Ascomycota | Unassigned | Unassigned | *Oidiodendron sp.* ^#^ | 99%(504/509) | JQ272359 | *Oidiodendron sp.* ^§^ | Plant roots in USA |
| 219 | 2 | 7 | Ascomycota | Helotiales | Helotiaceae | *Helotiaceae sp.* | 95%(368/386) | [HM141060](http://www.ncbi.nlm.nih.gov/nucleotide/307750665?report=genbank&log$=nucltop&blast_rank=1&RID=A1G7PDYP01R) | *Uncultured Ascomycota isolate* | Mycorrhizal root |
|  |  |  |  |  |  |  | 94%(365/390) | KF359569 | *Hymenoscyphus monotropae* | Plant root in USA |
| 220 | 1 | 4 | Basidiomycota | Filobasidiales | Unassigned | *Filobasidiales sp.* | 95%(372/392) | [GU328524](http://www.ncbi.nlm.nih.gov/nucleotide/209412925?report=genbank&log$=nucltop&blast_rank=1&RID=A1G7PDYP01R) | *Uncultured Basidiomycota clone* | Unreported |
|  |  |  |  |  |  |  | 94%(356/380) | KC894158 | *Cryptococcus sp.* | Moss in Antarctica |
| 221 | 1 | 4 | Ascomycota | Chaetothyriales | Unassigned | *Chaetothyriales sp.* | 89%(374/421) | [EF016384](http://www.ncbi.nlm.nih.gov/nucleotide/194304360?report=genbank&log$=nucltop&blast_rank=1&RID=A1G7PDYP01R) | *Cladophialophora minutissma* | Bryophytes |
| 222 | 5 | 1056 | Ascomycota | Helotiales | Unassigned | *Helotiales sp.* | 99%(500/501) | KF297268 | *Uncultured fungus clone*^§^ | Arctic soil in Canada (76.23N; 119.30W) |
|  |  |  |  |  |  |  | 96%(467/485) | FJ196296 | *Helotiales sp.* | Mycorrhizal fungi in Taiwan |
| 223 | 1 | 3 | Basidiomycota | Agaricales | Tricholomataceae | *Gymnopus sp.* ^#^ | 95%(445/468) | [AY256709](http://www.ncbi.nlm.nih.gov/nucleotide/387145937?report=genbank&log$=nucltop&blast_rank=1&RID=A1G7PDYP01R) | *Gymnopus luxurians* | USA |
| 224 | 2 | 14 | Ascomycota | Lecanorales | Unassigned | *Lecanorales sp.* | 90%(359/398) | [KC965160](http://www.ncbi.nlm.nih.gov/nucleotide/305671307?report=genbank&log$=nucltop&blast_rank=1&RID=9R00Y6YM014) | *Uncultured fungus clone* | Arctic soil in USA (70.31N; 147.99W) |
|  |  |  |  |  |  |  | 93%(325/348) | AM292669 | *Bilimbia microcarpa* | Unreported |
| 225 | 2 | 170 | Ascomycota | Baeomycetales | Unassigned | *Baeomycetales sp.* | 85%(443/519) | KF800339 | *Uncultured fungus clone* | House dust in USA |
|  |  |  |  |  |  |  | 85%(424/499) | FJ903326 | *Sarea sp.* | Plant in Latvia |
| 226 | 1 | 2 | Basidiomycota | Polyporales | Unassigned | *Polyporales sp.* | 87%(424/489) | [JQ673132](http://www.ncbi.nlm.nih.gov/nucleotide/294861918?report=genbank&log$=nucltop&blast_rank=1&RID=9R00Y6YM014) | *Skeletocutis chrysella* | Plant in USA |
| 227 | 1 | 3 | Ascomycota | Helotiales | Helotiaceae | *Helotiaceae sp.* | 92%(459/498) | GU174356 | *Uncultured fungus clone* | Soil in USA |
|  |  |  |  |  |  |  | 91%(444/489) | FJ440900 | *Uncultured Helotiaceae clone* | Plant root in USA |
| 228 | 3 | 9 | Ascomycota | Capnodiales | Unassigned | *Capnodiales sp.* | 91%(506/556) | [KF800576](http://www.ncbi.nlm.nih.gov/nucleotide/387966464?report=genbank&log$=nucltop&blast_rank=1&RID=9R00Y6YM014) | *Uncultured fungus clone* | Indoor air in USA |
|  |  |  |  |  |  |  | 91%(478/528) | KC311489 | *Pseudotaeniolina globosa* | Soil in Austria |
| 229 | 1 | 15 | Unassigned | Unassigned | Unassigned | *Fungus sp.* | 87%(400/461) | KF274105 | *Uncultured fungus clone* | Wood stump in Finland |
| 230 | 6 | 15 | Ascomycota | Capnodiales | Unassigned | *Capnodiales sp.* | 95%(464/487) | [JQ247386](http://www.ncbi.nlm.nih.gov/nucleotide/353681906?report=genbank&log$=nucltop&blast_rank=1&RID=A1G7PDYP01R) | *Uncultured fungus clone* | Soil in Mexico (32.41N; 11.68W) |
|  |  |  |  |  |  |  | 93%(482/520) | JF499843 | *Penidiella ellipsoidea* | Leaf bracts in South Africa |
| 231 | 1 | 4 | Ascomycota | Helotiales | Helotiaceae | *Helotiaceae sp.* | 96%(469/489) | [KC965927](http://www.ncbi.nlm.nih.gov/nucleotide/307750518?report=genbank&log$=nucltop&blast_rank=1&RID=A1G7PDYP01R) | *Uncultured fungus clone* | Arctic soil in Canada (76.23N; 119.30W) |
|  |  |  |  |  |  |  | 92%(468/508) | KF359569 | *Hymenoscyphus monotropae* | Plant root in USA |
| 232 | 2 | 79 | Unassigned | Unassigned | Unassigned | *Fungus sp.* | 96%(516/540) | KF274449 | *Uncultured fungus clone* | Wood stump in Finland |
| 233 | 1 | 4 | Ascomycota | Unassigned | Unassigned | *Ascomycota sp.* | 96%(462/482) | KC9656903 | *Uncultured fungus clone* | Arctic soil in Canada (76.23N,119.30W) |
|  |  |  |  |  |  |  | 84%(413/491) | HM161487 | *Umbilicaria haplocarpa* | Unreported |
| 234 | 3 | 8 | Ascomycota | Verrucariales | Verrucariaceae | *Verrucariaceae sp.* | 99%(511/512) | [KF296962](http://www.ncbi.nlm.nih.gov/nucleotide/387966481?report=genbank&log$=nucltop&blast_rank=1&RID=9R00Y6YM014) | *Uncultured fungus clone*^§^ | Arctic soil in Canada (76.23N,119.30W) |
|  |  |  |  |  |  |  | 93%(465/501) | JF509161 | *Agonimia repleta* | Poland |
| 235 | 1 | 2 | Ascomycota | Helotiales | Unassigned | *Helotiales sp.* | 99%(470/473) | JQ991730 | *Uncultured Helotiales clone*^§^ | Ectomycorrhizal root tips |
| 236 | 1 | 17 | Ascomycota | Unassigned | Unassigned | *Ascomycota sp.* | 99%(489/494) | KF296938 | *Uncultured fungus clone*^§^ | Arctic soil in Canada (76.23N; 119.30W) |
|  |  |  |  |  |  |  | 86%(405/482) | AF096216 | *Umbilicaria hyperborea* | Unreported |
| 237 | 1 | 2 | Ascomycota | Unassigned | Unassigned | *Ascomycota sp.* | 86% (438/570) | [JQ666530](http://www.ncbi.nlm.nih.gov/nucleotide/408877231?report=genbank&log$=nucltop&blast_rank=1&RID=9R00Y6YM014) | *Uncultured soil fungus clone* | Forest soil in China |
|  |  |  |  |  |  |  | 86%(435/507) | HM239908 | *Uncultured Ascomycota clone* | Grassland soil in USA |
| 238 | 2 | 33 | Ascomycota | Lecanorales | Unassigned | *Lecanorales sp.* | 88%(458/523) | [AF228470](http://www.ncbi.nlm.nih.gov/nucleotide/307750666?report=genbank&log$=nucltop&blast_rank=1&RID=9R00Y6YM014) | *Letharia vulpina* | Unreported |
| 239 | 8 | 72 | Ascomycota | Helatiales | Unassigned | *Helotiales sp.* | 99%(515/516) | [JQ666492](http://www.ncbi.nlm.nih.gov/nucleotide/294861925?report=genbank&log$=nucltop&blast_rank=1&RID=9R00Y6YM014) | *Uncultured soil fungus clone*^§^ | Forest soil in China (Changbai mountain) |
|  |  |  |  |  |  |  | 97%(465/478) | FJ196296 | *Helotiales sp.* ^§^ | Mycorrhizal fungi in Taiwan |
| 240 | 2 | 18 | Ascomycota | Helotiales | Unassigned | *Cadophora luteo-olivacea** | 99%(524/526) | [HM116747](http://www.ncbi.nlm.nih.gov/nucleotide/198250463?report=genbank&log$=nucltop&blast_rank=1&RID=9R00Y6YM014) | *Cadophora luteo-olivacea*^§^ | Plant in New Zealand |
| 241 | 4 | 18 | Ascomycota | Capnodiales | Unassigned | *Capnodiales sp.* | 96%(487/507) | [KC965669](http://www.ncbi.nlm.nih.gov/nucleotide/75863826?report=genbank&log$=nucltop&blast_rank=1&RID=9R00Y6YM014) | *Uncultured fungus clone* | Arctic soil in USA (69.15N; 148.85W) |
|  |  |  |  |  |  |  | 91%(485/534) | JF499843 | *Penidella ellipsoidea* | Leaf bracts in South Africa |
| 242 | 1 | 7 | Ascomycota | Capnodiales | Unassigned | *Capnodiales sp.* | 88%(488/552) | GU931741 | *Uncultured Capnodiales clone* | House dust in Canada |
| 243 | 2 | 7 | Ascomycota | Unassigned | Unassigned | *Ascomycota sp.* | 83%(443/533) | DQ534484 | *Rhizoplaca aspidophora* | Antarctica |
| 244 | 3 | 283 | Ascomycota | Chaetothyriales | Unassigned | *Chaetothyriales sp.* | 89%(514/576) | [AM260819](http://www.ncbi.nlm.nih.gov/nucleotide/194578726?report=genbank&log$=nucltop&blast_rank=1&RID=9R00Y6YM014) | *Uncultured fungus clone* | Peat in United Kingdom |
|  |  |  |  |  |  |  | 88%(463/526) | EU139158 | *Capronia sp.* | Endolichenic fungi in China |
| 245 | 1 | 2 | Ascomycota | Verrucariales | Unassigned | *Verrucariales sp.* | 88%(301/344) | [FJ479632](http://www.ncbi.nlm.nih.gov/nucleotide/305671334?report=genbank&log$=nucltop&blast_rank=1&RID=9R00Y6YM014) | *Placopyrenium formosum* | Freshwater in United Kingdom |
| 246 | 3 | 21 | Ascomycota | Dothideales | Dothiocraceae | *Dothiocraceae sp.* | 92%(409/444) | KF274441 | *Uncultured fungus clone* | Wood stump in Finland |
|  |  |  |  |  |  |  | 91%(405/444) | KJ690089 | *Aurebasidium sp.* | Ectomycorrhiza |
| 247 | 1 | 9 | Ascomycota | Chaetothyriales | Unassigned | *Chaetothyriales sp.* | 94% (503/534) | [KF297156](http://www.ncbi.nlm.nih.gov/nucleotide/206114085?report=genbank&log$=nucltop&blast_rank=1&RID=9R00Y6YM014) | *Uncultured fungus clone* | Arctic soil in Canada (76.23N; 119.30W) |
|  |  |  |  |  |  |  | 87%(485/558) | GU998515 | *Uncultured Chaetothyriales clone* | Ectomycorrhiza root tip in USA |
| 248 | 2 | 14 | Ascomycota | Chaetothyriales | Unassigned | *Chaetothyriales sp.* | 99%(518/521) | KC965234 | *Uncultured fungus clone*^§^ | Arctic soil in Canada (76.23N; 119.30W) |
|  |  |  |  |  |  |  | 88%(484/552) | JQ272383 | *Herpotrichiellaceae sp.* | Plant root in USA |
| 249 | 3 | 236 | Ascomycota | Unassigned | Unassigned | *Ascomycota sp.* | 90%(466/519) | GU721967 | *Uncultured fungus clone* | Surface dust in USA |
|  |  |  |  |  |  |  | 89%(464/521) | AM901764 | *Uncultured ascomycete clone* | House dust in Finland |
| 250 | 6 | 28 | Ascomycota | Chaetothyriales | Herpotrichiellaceae | *Herpotrichiellaceae sp.* | 98%(534/546) | [FJ554179](http://www.ncbi.nlm.nih.gov/nucleotide/109390553?report=genbank&log$=nucltop&blast_rank=1&RID=9R00Y6YM014) | *Uncultured Herpotrichiellaceae clone*^§^ | Forest soil in Canada |
| 251 | 1 | 2 | Ascomycota | Capnodiales | Teratosphaeriaceae | *Teratosphaeriaceae sp.* | 99%(511/514) | [KC965353](http://www.ncbi.nlm.nih.gov/nucleotide/57869249?report=genbank&log$=nucltop&blast_rank=1&RID=9R00Y6YM014) | *Uncultured fungus clone*^§^ | Arctic soil in Canada (78.78N; 103.55W) |
|  |  |  |  |  |  |  | 90%(495/547) | GU570527 | *Devriesia pseudoamericana* | Fruit surface in Germany |
| 252 | 1 | 1795 | Ascomycota | Helotiales | Unassigned | *Geltingia associata** | 99%(494/495) | [KJ559542](http://www.ncbi.nlm.nih.gov/nucleotide/311305773?report=genbank&log$=nucltop&blast_rank=1&RID=9R00Y6YM014) | *Geltingia associata*^§^ | Lichenicolous fungi in Iceland |
| 253 | 3 | 6 | Ascomycota | Pleosporales | Phaeosphaeriaceae | *Phaeosphaeria triglochinicola** | 99%(511/518) | [AM901822](http://www.ncbi.nlm.nih.gov/nucleotide/124054937?report=genbank&log$=nucltop&blast_rank=1&RID=9R00Y6YM014) | *Uncultured ascomycete clone*^§^ | House dust in Finland |
|  |  |  |  |  |  |  | 99%(454/458) | AF439507 | *Phaeosphaeria triglochinicola*^§^ | Plant in Switzerland |
| 254 | 4 | 18 | Ascomycota | Lecideales | Lecideaceae | *Lecidea sp.* ^#^ | 96%(348/361) | [JN032491](http://www.ncbi.nlm.nih.gov/nucleotide/19913082?report=genbank&log$=nucltop&blast_rank=1&RID=9R00Y6YM014) | *Uncultured fungus clone* | Coniferous litter in boreal forest |
|  |  |  |  |  |  |  | 96%(341/356) | DQ534472 | *Lecidea cancriformis* | Antarctica |
| 255 | 4 | 5 | Ascomycota | Eurotiales | Aspergillaceae | *Aspergillus niger** | 99% (534/542) | [EF105366](http://www.ncbi.nlm.nih.gov/nucleotide/401772527?report=genbank&log$=nucltop&blast_rank=1&RID=9R00Y6YM014) | *Aspergillus niger*^§^ | Unreported |
| 256 | 1 | 334 | Ascomycota | Pertusariales | Unassigned | *Pertusariales sp.* | 88%(433/492) | HQ631037 | *Scytalidium sp.* | Unreported |
| 257 | 2 | 10 | Ascomycota | Verrucariales | Verrucariaceae | *Polyblastia sp.* ^#^ | 96%(514/533) | [KF296738](http://www.ncbi.nlm.nih.gov/nucleotide/332015805?report=genbank&log$=nucltop&blast_rank=1&RID=9R00Y6YM014) | *Uncultured fungus clone* | Arctic soil in Canada (76.23N; 119.30W) |
|  |  |  |  |  |  |  | 96%(512/532) | EU364560 | *Polyblastia terrestris* | Unreported |
| 258 | 14 | 849 | Ascomycota | Helotiales | Leotiaceae | *Leotiaceae sp.* | 99%(458/459) | KC[966006](http://www.ncbi.nlm.nih.gov/nucleotide/323320653?report=genbank&log$=nucltop&blast_rank=2&RID=9R00Y6YM014) | *Uncultured fungus clone*^§^ | Arctic soil in Canada (78.78N; 103.55W) |
|  |  |  |  |  |  |  | 93%(478/513) | KC834057 | *Gorgomyces honrubiae* | Unreported |
| 259 | 8 | 113 | Ascomycota | Unassigned | Unassigned | *Ascomycota sp.* | 93%(465/500) | [KF297261](http://www.ncbi.nlm.nih.gov/nucleotide/268636587?report=genbank&log$=nucltop&blast_rank=1&RID=9R00Y6YM014) | *Uncultured fungus clone* | Arctic soil in Canada (76.23N; 119.30W) |
|  |  |  |  |  |  |  | 91%(435/479) | FJ008690 | *Ascomycota sp.* | Endolichenic fungi |
| 260 | 7 | 113 | Ascomycota | Saccharomycetales | Saccharomycetaceae | *Pichia pastoris** | 98%(372/380) | FR839630 | *Pichia pastoris*^§^ | Unreported |
| 261 | 2 | 154 | Ascomycota | Chaetothyriales | Unassigned | *Chaetothyriales sp.* | 89% (474/532) | [KC965841](http://www.ncbi.nlm.nih.gov/nucleotide/344333452?report=genbank&log$=nucltop&blast_rank=1&RID=9R00Y6YM014) | *Uncultured fungus clone* | Arctic soil in USA (69.67N; 148.72W) |
|  |  |  |  |  |  |  | 87%(428/494) | EU139146 | *Capronia sp.* | Endolichenic fungi in lichen |
| 262 | 1 | 7 | Ascomycota | Xylariales | Apiosporaceae | *Arthrinium arundinis** | 99%(466/471) | [KF693784](http://www.ncbi.nlm.nih.gov/nucleotide/327532804?report=genbank&log$=nucltop&blast_rank=2&RID=9R00Y6YM014) | *Arthrinium arundinis*^§^ | Unreported |
| 263 | 3 | 120 | Basidiomycota | Tremellales | Unassigned | *Tremellales sp.* | 87%(419/480) | [JN053490](http://www.ncbi.nlm.nih.gov/nucleotide/110555843?report=genbank&log$=nucltop&blast_rank=2&RID=9R00Y6YM014) | *Tremella cetrariicola* | Unreported |
| 264 | 1 | 22 | Ascomycota | Capnodiales | Teratosphaeriaceae | *Oleoguttula sp.* ^#^ | 96%(494/514) | [KC966025](http://www.ncbi.nlm.nih.gov/nucleotide/99866600?report=genbank&log$=nucltop&blast_rank=1&RID=9R00Y6YM014) | *Uncultured fungus clone* | Arctic soil in Canada (76.23N; 119.30W) |
|  |  |  |  |  |  |  | 96%(446/467) | KF309972 | *Oleoguttula mirabilis* | Unreported |
| 265 | 2 | 16 | Ascomycota | Lecanorales | Unassigned | *Lecanorales sp.* | 97%(263/270) | KF297149 | *Uncultured fungus clone*^§^ | Arctic soil in Canada (76.23N; 119.30W) |
|  |  |  |  |  |  |  | 85%(229/271) | JQ301699 | *Cetraria islandica* | Unreported |
| 266 | 1 | 19 | Ascomycota | Lecanorales | Unassigned | *Lecanorales sp.* | 89% (488/546) | KF274432 | *Uncultured fungus clone* | Wood stump in Finland |
|  |  |  |  |  |  |  | 87%(461/528) | GU183183 | *Parmeliacae sp.* | Unreported |
| 267 | 1 | 23 | Ascomycota | Lecanorales | Unassigned | *Lecanorales sp.* | 91% (453/498) | [KC965855](http://www.ncbi.nlm.nih.gov/nucleotide/305671360?report=genbank&log$=nucltop&blast_rank=1&RID=9R00Y6YM014) | *Uncultured fungus clone* | Arctic soil in Canada (78.78N; 103.55W) |
|  |  |  |  |  |  |  | 87%(456/526) | JX036118 | *Rhizoplaca sp.* | Antarctica |
| 268 | 3 | 7 | Ascomycota | Lecanorales | Unassigned | *Lecanorales sp.* | 92%(414/449) | [HQ605940](http://www.ncbi.nlm.nih.gov/nucleotide/370316654?report=genbank&log$=nucltop&blast_rank=1&RID=9R00Y6YM014) | *Porpidia macrocarpa* | Turkey |
| 269 | 3 | 43 | Ascomycota | Unassigned | Unassigned | *Ascomycota sp.* | 90%(467/517) | [HM240000](http://www.ncbi.nlm.nih.gov/nucleotide/310894137?report=genbank&log$=nucltop&blast_rank=3&RID=9R00Y6YM014) | *Uncultured Ascomycota clone* | Grassland soil in USA |
| 270 | 2 | 7 | Ascomycota | Verrucariales | Verrucariaceae | *Sporodictyon schaererianum** | 99%(422/424) | [EU697642](http://www.ncbi.nlm.nih.gov/nucleotide/208401005?report=genbank&log$=nucltop&blast_rank=1&RID=9R00Y6YM014) | *Sporodictyon schaererianum*^§^ | Greenland |
| 271 | 1 | 2 | Ascomycota | Chaetothyriales | Unassigned | *Chaetothyriales sp.* | 99%(506/507) | [KF297153](http://www.ncbi.nlm.nih.gov/nucleotide/305671360?report=genbank&log$=nucltop&blast_rank=1&RID=9R00Y6YM014) | *Uncultured fungus clone*^§^ | Arctic soil in Canada (76.23N; 119.30W) |
|  |  |  |  |  |  |  | 85%(448/528) | HQ634653 | *Chaetothyriales sp.* | Ant |
| 272 | 2 | 42 | Ascomycota | Pertusariales | Megasporaceae | *Aspicillia sp.* ^#^ | 96%(430/448) | [EU057949](http://www.ncbi.nlm.nih.gov/nucleotide/124441653?report=genbank&log$=nucltop&blast_rank=1&RID=9R00Y6YM014) | *Aspicillia zonata* | Sweden |
| 273 | 9 | 79 | Ascomycota | Capnodiales | Teratosphaeriaceae | *Penidiella sp.* ^#^ | 95%(408/430) | [HM240000](http://www.ncbi.nlm.nih.gov/nucleotide/307750518?report=genbank&log$=nucltop&blast_rank=1&RID=9R00Y6YM014) | *Uncultured Ascomycota clone* | Grassland soil in Canada |
|  |  |  |  |  |  |  | 96%(392/408) | JF499843 | *Penidiella ellipsoidea* | Leaf bracts in South Africa |
| 274 | 1 | 14 | Ascomycota | Hysteriales | Gloniaceae | *Cenococcum geophilum** | 97% (524/541) | [JQ711896](http://www.ncbi.nlm.nih.gov/nucleotide/357216876?report=genbank&log$=nucltop&blast_rank=1&RID=9R00Y6YM014) | *Cenococcum geophilum*^§^ | Ectomycorrhiza in Canada |
| 275 | 2 | 42 | Ascomycota | Unassigned | Unassigned | *Ascomycota sp.* | 89%(396/446) | KF800339 | *Uncultured fungus clone* | House dust in USA |
|  |  |  |  |  |  |  | 89%(395/446) | AM901772 | *Uncultured ascomycete clone* | House dust in Finland |
| 276 | 4 | 582 | Ascomycota | Hypocreales | Unassigned | *Hypocreales sp.* | 99% (519/522) | [KC965446](http://www.ncbi.nlm.nih.gov/nucleotide/325516921?report=genbank&log$=nucltop&blast_rank=2&RID=9R00Y6YM014) | *Uncultured fungus clone*^§^ | Arctic soil in Canada (76.23N; 119.30W) |
|  |  |  |  |  |  |  | 88%(490/559) | AF081472 | *Stachybotrys dichroa* | Unreported |
| 277 | 6 | 145 | Ascomycota | Capnodiales | Teratosphaeriaceae | *Teratosphaeriaceae sp.* | 97%(505/521) | [AM901736](http://www.ncbi.nlm.nih.gov/nucleotide/289190150?report=genbank&log$=nucltop&blast_rank=1&RID=A1G7PDYP01R) | *Uncultured ascomycete clone*^§^ | House dust in Finland |
|  |  |  |  |  |  |  | 93%(482/518) | GU570527 | *Devriesia pseudoamericana* | Fruit surface in Germany |
| 278 | 7 | 357 | Ascomycota | Chaetothyriales | Unassigned | *Chaetothyriales sp.* | 99%(496/503) | [KF296827](http://www.ncbi.nlm.nih.gov/nucleotide/193885584?report=genbank&log$=nucltop&blast_rank=1&RID=A1G7PDYP01R) | *Uncultured fungus clone*^§^ | Arctic soil in Canada (78.78N; 103.55W) |
|  |  |  |  |  |  |  | 87%(483/553) | JQ272383 | *Herpotrichiellaceae sp.* | Plant root in USA |
| 279 | 1 | 64 | Ascomycota | Lecanorales | Lecanoraceae | *Lecanoraceae sp.* | 94%(487/518) | [KC966001](http://www.ncbi.nlm.nih.gov/nucleotide/300394356?report=genbank&log$=nucltop&blast_rank=1&RID=A1G7PDYP01R) | *Uncultured fungus clone* | Arctic soil in Canada (78.78N; 103.55W) |
|  |  |  |  |  |  |  | 94%(473/502) | HQ605936 | *Lecidella elaeochroma* | Turkey |
| 280 | 1 | 27 | Ascomycota | Unassigned | Unassigned | *Ascomycota sp.* | 95%(251/264) | [FR682211](http://www.ncbi.nlm.nih.gov/nucleotide/325516886?report=genbank&log$=nucltop&blast_rank=1&RID=A1G7PDYP01R) | *Uncultured Ascomycota clone* | Houst dust in Finland |
| 281 | 2 | 21 | Ascomycota | Helotiales | Unassigned | *Helotiales sp.* | 99% (507/512) | [KF618056](http://www.ncbi.nlm.nih.gov/nucleotide/305671350?report=genbank&log$=nucltop&blast_rank=1&RID=A1G7PDYP01R) | *Uncultured fungus clone*^§^ | Forest soil in USA (64.91N; 147.82W) |
|  |  |  |  |  |  |  | 93%(453/487) | EF029215 | *Spirosphaera beverwijkiana* | Unreported |
| 282 | 1 | 2 | Ascomycota | Capnodiales | Teratosphaeriaceae | *Teratosphaeriaceae sp.* | 93%(381/408) | [KC966173](http://www.ncbi.nlm.nih.gov/nucleotide/344333370?report=genbank&log$=nucltop&blast_rank=1&RID=A1G7PDYP01R) | *Uncultured fungus clone* | Arctic soil in Canada (76.23N; 119.30W) |
|  |  |  |  |  |  |  | 91%(393/434) | JN232423 | *Teratosphaeria pseudafricana* | Plant in Brazil |
| 283 | 2 | 3 | Ascomycota | Verrucariales | Verrucariaceae | *Atla alpina* | 97%(499/515) | KC966360 | *Uncultured fungus clone*^§^ | Arctic soil in Canada (73.22N;159.56W) |
|  |  |  |  |  |  |  | 99%(464/465) | EU697726 | *Atla alpina*^§^ | Sweden |
| 284 | 3 | 128 | Ascomycota | Capnodiales | Mycosphaerellaceae | *Mycocentrospora sp.* ^#^ | 100%(512/512) | KC966327 | *Uncultured fungus clone*^§^ | Arctic soil in Canada (78.78N; 103.55W) |
|  |  |  |  |  |  |  | 97%(488/533) | FJ911882 | *Mycocentrospora sp.* ^§^ | Plant leaves in Antarctica |
| 285 | 2 | 7 | Ascomycota | Verrucariales | Verrucariaceae | *Polyblastia wheldonii** | 99%(512/513) | KC966360 | *Uncultured fungus clone*^§^ | Arctic soil in Canada (73.22N; 119.56W) |
|  |  |  |  |  |  |  | 98%(462/472) | EU553497 | *Polyblastia wheldonii*^§^ | Lichen in Sweden |
| 286 | 1 | 7 | Ascomycota | Lecanorales | Lecanoraceae | *Carbonea sp.* ^#^ | 95%(459/482) | JX036120 | *Carbonea sp.* | Antarctica |
| 287 | 1 | 2 | Basidiomycota | Boletales | Boletaceae | *Boletaceae sp.* | 99%(393/397) | JQ9911654 | *Uncultured Boletaceae clone*^§^ | Ectomycorrhizal root tips in China |
| 288 | 1 | 9 | Ascomycota | Capnodiales | Cladosporiaceae | *Cladosporiaceae sp.* | 95% (467/493) | KC965721 | *Uncultured fungus clone* | Arctic soil in Canada (78.78N; 103.55W) |
|  |  |  |  |  |  |  | 90%(474/525) | JF951145 | *Rachicladosporium pini* | Plant in Netherlands |
| 289 | 3 | 9 | Ascomycota | Capnodiales | Unassigned | *Capnodiales sp.* | 99%(534/541) | FJ553032 | *Uncultured Dothideomycetidae clone*^§^ | Forest soil in Canada |
|  |  |  |  |  |  |  | 89%(494/554) | EU707864 | *Teratosphaeria jonkershoekensis* | Plant in South Africa |
| 290 | 1 | 22 | Ascomycota | Helotiales | Helotiaceae | *Helotiaceae sp.* | 94%(507/542) | AB705232 | *Hymenoscyphus sp.* | Unreported |
| 291 | 2 | 14 | Ascomycota | Unassigned | Unassigned | *Ascomycota sp.* | 99% (531/534) | KF296903 | *Uncultured fungus clone*^§^ | Arctic soil in Canada (76.23N; 119.30W) |
|  |  |  |  |  |  |  | 90%(515/575) | FN555433 | *Uncultured Ascomycota clone* | Liverworts in Antarctica |
| 292 | 7 | 46 | Ascomycota | Unassigned | Unassigned | *Ascomycota sp.* | 97%(487/503) | KC965719 | *Uncultured fungus clone*^§^ | Arctic soil in Canada (78.78N; 103.55W) |
|  |  |  |  |  |  |  | 87%(438/506) | EF373563 | *Ascomycete sp.* | Endolichenic fungi |
| 293 | 1 | 3 | Ascomycota | Helotiales | Unassigned | *Ascomycota sp.* | 99%(508/509) | HG738855 | *Scytalidium thermophilum*^§^ | soil |
| 294 | 2 | 5 | Ascomycota | Pleosporales | Unassigned | *Coniothyrium fuckelii** | 99%(503/506) | KC966092 | *Uncultured fungus clone*^§^ | Arctic soil in Canada (76.23N; 119.30W) |
|  |  |  |  |  |  |  | 97%(514/531) | AB665314 | *Coniothyrium fuckelii*^§^ | Plant in Japan |
| 295 | 2 | 3 | Ascomycota | Unassigned | Unassigned | *Ascomycota sp.* | 90%(348/386) | KF800302 | *Uncultured fungus clone* | House dust in USA |
|  |  |  |  |  |  |  | 89%(340/383) | AM901817 | *Uncultured ascomycete clone* | House dust in Finland |
| 296 | 3 | 21 | Basidiomycota | Tremellales | Unassigned | *Dioszegia fristingensis** | 99%(436/437) | KC965538 | *Uncultured fungus clone*^§^ | Arctic soil in Canada (78.78N; 103.55W) |
|  |  |  |  |  |  |  | 100%(409/409) | JQ857038 | *Dioszegia fristingensis*^§^ | Antarctica |
| 297 | 4 | 37 | Basidiomycota | Cystofilobasidiales | Cystofilobasidiaceae | *Mrakia blollopis** | 100%(522/522) | AM901861 | *Uncultured basidiomycete clone*^§^ | House dust in Finland |
|  |  |  |  |  |  |  | 99%(501/505) | AB916516 | *Mrakia blollopis*^§^ | Arctic bird feather in Norway |
| 298 | 2 | 94 | Ascomycota | Chaetothyriales | Unassigned | *Chaetothyriales sp.* | 92% (440/477) | KC965473 | *Uncultured fungus clone* | Arctic soil in Canada (73.22N; 119.56W) |
|  |  |  |  |  |  |  | 87%(384/439) | AJ971455 | *Coniosporium sp.* | Marble monument in Turkey |
| 299 | 2 | 360 | Ascomycota | Chaetothyriales | Unassigned | *Chaetothyriales sp.* | 99%(529/531) | KF297101 | *Uncultured fungus clone*^§^ | Arctic soil in USA (70.31N; 147.99W) |
|  |  |  |  |  |  |  | 88%(508/578) | JQ272383 | *Herpotrichiellaceae sp.* | Plant in USA |
| 300 | 1 | 6 | Basidiomycota | Sebacinales | Sebacinaceae | *Sebacina sp.* ^#^ | 95%(448/473) | EU909222 | *Uncultured Sebacina mycobioint* | Liverworts (*Riccardia latifrons*) |
| 301 | 1 | 5 | Unassigned | Unassigned | Unassigned | *Fungus sp.* | 99%(478/480) | KF296725 | *Uncultured fungus clone*^§^ | Arctic soil in Canada (76.23N; 119.30W) |
| 302 | 3 | 2161 | Ascomycota | Verrucariales | Verrucariaceae | *Polyblastia sp.* ^#^ | 99%(499/502) | KC965806 | *Uncultured fungus clone*^§^ | Arctic Soil in Canada (76.23N;119.30W) |
|  |  |  |  |  |  |  | 96%(443/461) | EU553497 | *Polyblastia wheldonii* | Sweden |
| 303 | 1 | 6 | Ascomycota | Helotiales | Unassigned | *Helotiales sp.* | 87%(430/492) | HQ845751 | *Helotiales sp.* | Plant in USA |
| 304 | 1 | 6 | Ascomycota | Lecanorales | Unassigned | *Lecanorales sp.* | 88%(460/521) | KF274472 | *Uncultured fungus clone* | Wood stump in Finland |
|  |  |  |  |  |  |  | 87%(450/518) | GU183183 | *Parmeliaceae sp.* | Unreported |
| 305 | 2 | 31 | Zygomycota | Mortierellales | Mortierellaceae | *Mortierella sp.* ^#^ | 99%(498/502) | KC965282 | *Uncultured fungus clone*^§^ | Arctic soil in Canada (78.78N; 103.55W) |
|  |  |  |  |  |  |  | 95%(501/528) | JF439485 | *Mortierella elongata* | Unreported |
| 306 | 1 | 6 | Ascomycota | Capnodiales | Unassigned | *Capnodiales sp.* | 95%(379/419) | KC965673 | *Uncultured fungus clone* | Arctic soil in USA (69.15N; 148.85W) |
|  |  |  |  |  |  |  | 94%(315/336) | JF691177 | *Uncultured Capnodiales clone* | Plant root in Reunion |
| 307 | 4 | 14 | Ascomycota | Unassigned | Unassigned | *Ascomycota sp.* | 99%(489/495) | KF297261 | *Uncultured fungus clone*^§^ | Arctic soil in Canada (76.23N; 119.30W) |
|  |  |  |  |  |  |  | 91%(428/470) | FJ008690 | *Ascomycota sp.* | Endolichenic fungi |
| 308 | 1 | 20 | Ascomycota | Pezizales | Pyronemataceae | *Pyronemataceae sp.* | 97%(479/496) | KF617920 | *Uncultured fungus clone*^§^ | Forest soil in USA (64.99N; 147.65W) |
|  |  |  |  |  |  |  | 91%(466/513) | EU669387 | *Pseudaleuria quinaultiana* | Unreported |
| 309 | 1 | 14 | Basidiomycota | Agaricales | Strophariaceae | *Galerina unicolor* | 99%(465/466) | JF908013 | *Galerina unicolor*^§^ | Unreported |
| 310 | 3 | 583 | Ascomycota | Capnodiales | Cladosporiaceae | *Cladosporium sp.* ^#^ | 96%(386/401) | KC966013 | *Uncultured fungus clone* | Arctic soil in Canada (76.23N; 119.30W) |
|  |  |  |  |  |  |  | 95%(333/349) | FJ378727 | *Uncultured Cladosporium isolate* | Plant in Himalaya |
| 311 | 4 | 13 | Ascomycota | Chaetothyriales | Unassigned | *Chaetothyriales sp.* | 96%(491/510) | KC965234 | *Uncultured fungus clone* | Arctic soil in Canada (76.23N; 119.30W) |
|  |  |  |  |  |  |  | 88%(473/540) | JQ272383 | *Herpotrichiellaceae sp.* | USA |
| 312 | 1 | 2 | Basidiomycota | Agaricales | Tricholomataceae | *Arrhenia lobata* | 99%(480/483) | [U66429](http://www.ncbi.nlm.nih.gov/nucleotide/266634419?report=genbank&log$=nucltop&blast_rank=1&RID=9R00Y6YM014) | *Arrhenia lobata*^§^ | Unreported |
| 313 | 1 | 9 | Ascomycota | Verrucariales | Verrucariaceae | *Polyblastia sp.* ^#^ | 99%(503/504) | KC965931 | *Uncultured fungus clone*^§^ | Arctic soil in Canada 76.23N; 119.30W) |
|  |  |  |  |  |  |  | 99%(457/463) | EU559739 | *Polyblastia sp.* ^§^ | Lichen in Sweden |
| 314 | 3 | 20 | Ascomycota | Helotiales | Unassigned | *Helotiales sp.* | 96%(490/511) | KF646097 | *Leptodontidium orchidicola* | Plant roots in Lithuania |
| 315 | 1 | 9 | Ascomycota | Trapeliales | Trapeliaceae | *Trapeliaceae sp.* | 93%(500/539) | KC965545 | *Uncultured fungus clone* | Arctic soil in Canada (76.23N; 119.30W) |
|  |  |  |  |  |  |  | 94%(440/469) | HQ650633 | *Placynthiella uliginosa* | Unreported |
| 316 | 6 | 181 | Ascomycota | Capnodiales | Teratosphaeriaceae | *Teratosphaeriaceae sp.* | 99%(502/508) | KC965774 | *Uncultured fungus clone*^§^ | Arctic soil in Canada (76.23N; 119.30W) |
|  |  |  |  |  |  |  | 93%(499/535) | GU570527 | *Devriesia pseudoamericana* | Fruit surface in Germany |
| 317 | 1 | 20 | Ascomycota | Helotiales | Unassigned | *Helotiales sp.* | 98%(491/502) | KF296959 | *Uncultured fungus clone*^§^ | Arctic soil in Canada(76.23N; 119.30W) |
|  |  |  |  |  |  |  | 93%(300/324) | KJ188690 | *Leptodontidiu orchidicola* | Unreported |
| 318 | 1 | 9 | Ascomycota | Chaetothyriales | Unassigned | *Chaetothyriales sp.* | 98%(513/524) | KC965863 | *Uncultured fungus clone*^§^ | Arctic soil in Canada (76.23N; 119.30W) |
|  |  |  |  |  |  |  | 86%(479/554) | EU139138 | *Rhinocladiella sp.* | Endolichenic fungi |
| 319 | 1 | 71 | Ascomycota | Lecanorales | Unassigned | *Lecanorales sp.* | 98%(519/527) | FJ554208 | *Uncultured Lecanorineae clone*^§^ | Forest soil in Canada |
| 320 | 1 | 21 | Ascomycota | Chaetothyriales | Herpotrichiellaceae | *Herpotrichiellaceae sp.* | 97%(509/525) | KC965763 | *Uncultured fungus clone*^§^ | Arctic soil in USA (69.15N; 148.85W) |
|  |  |  |  |  |  |  | 95%(424/447) | KF636410 | *Herpotrichiellaceae sp.* | Liverwort in Antarctica |
| 321 | 5 | 21 | Basidiomycota | Malasseziales | Unassigned | *Malassezia sp.* ^#^ | 99%(502/503) | AM901750 | *Uncultured basidiomycete clone*^§^ | House dust in Finland |
|  |  |  |  |  |  |  | 99%(485/489) | KC785585 | *Uncultured Malassezia clone*^§^ | Soil in Antarctica |
| 322 | 1 | 3 | Ascomycota | Rhytismatales | Rhytismataceae | *Rhytismataceae sp.* | 99%(489/493) | JQ272405 | *Rhytismataceae sp.* ^§^ | Plant root in USA |
| 323 | 4 | 71 | Ascomycota | Helotiales | Unassigned | *Mycoarthris sp.* ^#^ | 98%(507/520) | GU931728 | *Uncultured Helotiales clone*^§^ | House dust in Canada |
|  |  |  |  |  |  |  | 98%(489/498) | JF449666 | *Uncultured Mycoarthris clone*^§^ | Leaf litter in Austria |
| 324 | 7 | 321 | Ascomycota | Verrucariales | Verrucariaceae | *Polyblastia sp.* ^#^ | 98%(487/495) | KC966180 | *Uncultured fungus clone*^§^ | Arctic soil in USA (69.67N; 148.72W) |
|  |  |  |  |  |  |  | 92%(431/470) | EU559739 | *Polyblastia sp.* | Sweden |
| 325 | 1 | 6 | Ascomycota | Helotiales | Unassigned | *Helotiales sp.* | 99%(478/482) | KC966107 | *Uncultured fungus clone*^§^ | Arctic soil in Canada (76.23N; 119.30W) |
|  |  |  |  |  |  |  | 89%(436/489) | KF429260 | *Vibrissea sp.* | Chile |
| 326 | 1 | 3 | Ascomycota | Helotiales | Helotiaceae | *Helotiaceae sp.* | 90%(438/488) | FJ440900 | *Uncultured Helotiaceae clone* | Plant root in USA |
| 327 | 4 | 542 | Ascomycota | Helotiales | Unassigned | *Helotiales sp.* | 96%(492/511) | JN859275 | *Helotiales sp.* | Plant root in Hungary |
| 328 | 3 | 7 | Ascomycota | Helotiales | Sclerotiniaceae | *Sclerotinia sp.* ^#^ | 99%(522/526) | AJ279480 | *Sclerotinia sp.* ^§^ | Unreported |
| 329 | 1 | 11 | Basidiomycota | Tremellales | Unassigned | *Cryptococcus victoriae** | 94%(391/416) | KC884367 | *Unculured fungus clone*^§^ | soil samples in Qinghai-Tibet Plateau |
|  |  |  |  |  |  |  | 98%(323/330) | JX188144 | *Cryptococcus victoriae*^§^ | Plant (*Vitis vinifera*) in USA |
| 330 | 5 | 65 | Basidiomycota | Malasseziales | Malasseziaceae | *Malassezia restricta* | 99%(521/522) | JF497141 | *Uncultured fungus clone*^§^ | Unreported |
|  |  |  |  |  |  |  | 99%(520/522) | EU400587 | *Malassezia restricta*^§^ | Unreported |
| 331 | 4 | 13 | Ascomycota | Hypocreales | Nectriaceae | *Fusarium sp.* ^#^ | 100% (547/547) | FN397215 | *Uncultured fungus clone*^§^ | Soil in France (44.26N; 1.26E) |
|  |  |  |  |  |  |  | 100%(544/544) | JQ775555 | *Fusarium sp.*^§^ | Plant root in USA |
| 332 | 1 | 23 | Ascomycota | Verrucariales | Verrucariaceae | *Polyblastia inconspicua** | 93%(461/494) | [KC965507](http://www.ncbi.nlm.nih.gov/nucleotide/289190130?report=genbank&log$=nucltop&blast_rank=1&RID=A1G7PDYP01R) | *Uncultured fungus clone* | Arctic soil in USA (70.31N; 147.99W) |
|  |  |  |  |  |  |  | 99%(471/472) | JQ088075 | *Polyblastia inconspicua*^§^ | Arctic |
| 333 | 1 | 3 | Ascomycota | Unassigned | Unassigned | *Ascomycota sp.* | 87%(464/531) | HM239990 | *Uncultured Ascomycota clone* | Grassland soil in USA (Califorina) |
| 334 | 1 | 15 | Basidiomycota | Thelephorales | Thelephoraceae | *Tomentella sp.* ^#^ | 98%(479/491) | JQ711817 | *Tomentella sp.* ^§^ | Ectomycorrhiza in Canada (55.45N, 123.20W) |
| 335 | 6 | 622 | Ascomycota | Unassigned | Unassigned | *Ascomycota sp.* | 99%(508/513) | KC965719 | *Uncultured fungus clone*^§^ | Arctic soil in Canada (78.78N; 103.55W) |
|  |  |  |  |  |  |  | 87%(444/511) | EF373563 | *Ascomycete sp.* | Endolichenic fungi |
| 336 | 1 | 2 | Basidiomycota | Boletales | Boletaceae | *Boletellus obscurococcineus** | 99%(424/426) | AB973723 | *Boletellus obscurococcineus*^§^ | Japan |
| 337 | 3 | 12 | Ascomycota | Helotiales | Hyaloscyphaceae | *Hyaloscyphaceae sp.* | 96%(489/507) | KF274460 | *Unculturd fungus clone* | Wood stump in Finland |
|  |  |  |  |  |  |  | 94%(495/524) | DQ227258 | *Hyphodiscus hymeniophilus* | Unreported |
| 338 | 3 | 31 | Ascomycota | Helotiales | Unassigned | *Helotiales sp.* | 99%(515/518) | AB636433 | *Uncultured Helotiales clone*^§^ | Ectomycorrhizal root tip in China (Hunan) |
| 339 | 3 | 18 | Ascomycota | Helotiales | Leotiaceae | *Leotiaceae sp.* | 99%(481/484) | KF617583 | *Uncultured fungus clone*^§^ | Forest soil in USA (63.81N; 144.95W) |
|  |  |  |  |  |  |  | 92%(453/491) | KC834058 | *Gorgomyces hungaricus* | Decaying leaves |
| 340 | 2 | 62 | Ascomycota | Unassigned | Unassigned | *Ascomycota sp.* | 87%(439/503) | HQ605944 | *Rhizocarpon lavatum* | Unreported |
| 341 | 2 | 12 | Ascomycota | Chaetothyriales | Unassigned | *Chaetothyriales sp.* | 89%(402/452) | FJ265764 | *Rhinocladiella sp.* | Unreported |
| 342 | 3 | 46 | Basidiomycota | Tremellales | Tremellaceae | *Tremella sp.* ^#^ | 96%(429/445) | JN790587 | *Tremella diploschistina* | Lichenicolous fungi |
| 343 | 7 | 64 | Ascomycota | Saccharomycetales | Saccharomycetaceae | *Pichia pastoris** | 98%(368/379) | FN392325 | *Pichia pastoris*^§^ | Unreported |
| 344 | 3 | 5 | Ascomycota | Helotiales | Unassigned | *Helotiales sp.* | 99%(476/482) | GU998549 | *Uncultured Helotiales clone*^§^ | Plant root in USA (68.63N; 149.57W) |
| 345 | 1 | 150 | Ascomycota | Chaetothyriales | Unassigned | *Chaetorhyriales sp.* | 99%(537/538) | KC965864 | *Uncultured fungus clone*^§^ | Arctic soil in Canada (76.23N; 119.30W) |
|  |  |  |  |  |  |  | 86%(413/482) | EU139148 | *Capronia sp.* | Endolichenic fungi |
| 346 | 5 | 206 | Basidiomycota | Tremellales | Tremellaceae | *Tremellaceae sp.* | 99%(434/436) | AB476491 | *Uncultured fungus clone*^§^ | Plant roots in Sweden |
|  |  |  |  |  |  |  | 91%(413/456) | AF042417 | *Tremella foliacea* | Unreported |
| 347 | 1 | 14 | Ascomycota | Unassigned | Unassigned | *Ascomycota sp.* | 86%(448/521) | HQ605942 | *Rhizocarpon petraeum* | Turkey |
| 348 | 14 | 1678 | Ascomycota | Unassigned | Unassigned | *Ascomycota sp.* | 99%(444/446) | KC966342 | *Uncultured fungus clone*^§^ | Arctic soil in Canada (76.23N; 119.30W) |
|  |  |  |  |  |  |  | 88%(379/430) | EF373563 | *Ascomycete sp.* | Tuckermannopsis chlorophylla |
| 349 | 1 | 50 | Ascomycota | Lecanorales | Unassigned | *Lecanorales sp.* | 91%(458/502) | AY679138 | *Uncultured ascomycete clone* | Granitic rock |
|  |  |  |  |  |  |  | 91%(463/510) | HQ605939 | *Porpidia musiva* | Turkey |
| 350 | 1 | 5 | Ascomycota | Mytilinidiales | Gloniaceae | *Gloniaceae sp.* | 99%(503/508) | DQ273286 | *Uncultured ascomycete clone*^§^ | Bulked root pools in USA (California) |
|  |  |  |  |  |  |  | 93%(430/464) | HE814233 | *Cenococcum sp.* | Plant root in China |
| 351 | 3 | 78 | Ascomycota | Unassigned | Unassigned | *Ascomycota sp.* | 82%(477/580) | EU490066 | *Uncultured ascomycete clone* | Soil in USA |
| 352 | 2 | 101 | Ascomycota | Chaetothyriales | Herpotrichiellaceae | *Herpotrichiellaceae sp.* | 99%(457/462) | FJ553396 | *Uncultured Herpotrichiellaceae clone*^§^ | Forest soil in Canada |
| 353 | 1 | 5 | Basidiomycota | Filobasidiales | Unassigned | *Cryptococcus gilvescens** | 99%(477/480) | KC965257 | *Uncultured fungus clone*^§^ | Arctic soil in USA (70.31N; 147.99W) |
|  |  |  |  |  |  |  | 100%(467/467) | AB032678 | *Cryptococcus gilvescens*^§^ | Unreported |
| 354 | 1 | 9 | Ascomycota | Hypocreales | Unassigned | *Hypocreales sp.* | 99%(492/495) | KF297027 | *Uncultured fungus clone*^§^ | Arctic soil in Canada (76.23N; 119.30W) |
|  |  |  |  |  |  |  | 91%(363/401) | DQ393594 | *Acremonium cyanophagus* | Germany |
| 355 | 4 | 162 | Ascomycota | Capnodiales | Unassigned | *Capnodiales sp.* | 95%(523/548) | KC966158 | *Uncultured fungus clone* | Arctic soil in USA (69.15N; 148.85W) |
|  |  |  |  |  |  |  | 89%(497/560) | EU707864 | *Teratosphaeria jonkershoekensis* | South Africa |
| 356 | 2 | 14 | Ascomycota | Capnodiales | Unassigned | *Capnodiales sp.* | 92%(358/390) | JQ272378 | *Capnodiales sp.* | Plant root in USA |
| 357 | 5 | 20 | Basidiomycota | Filobasidiales | Filobasidiaceae | *Cryptococcus tephrensis** | 99%(509/510) | DQ000318 | *Cryptococcus tephrensis*^§^ | Plant leaves |
| 358 | 1 | 2 | Unassigned | Unassigned | Unassigned | *Fungus sp.* | 84%(440/523) | KF274452 | *Uncultured fungus clone* | Wood stump in Finland |
| 359 | 1 | 34 | Ascomycota | Chaetothyriales | Unassigned | *Capronia peltigerae** | 99%(548/552) | FJ554031 | *Uncultured Lecanoromycetidae clone*^§^ | Forest soil in Canada |
|  |  |  |  |  |  |  | 98%(482/494) | HQ709322 | *Capronia peltigerae*^§^ | Lichenicolous fungi |
| 360 | 2 | 6 | Ascomycota | Xylariales | Amphisphaeriaceae | *Amphisphaeriaceae sp.* | 90%(477/531) | JF440975 | *Leiosphaerella lycopodina* | Unreported |
| 361 | 1 | 7 | Basidiomycota | Russulales | Russulaceae | *Russula sp.* ^#^ | 98%(474/485) | DQ990845 | *Uncultured ectomycorrhiza (Russula)* ^§^ | Ectomycorrhizal mantle |
| 362 | 5 | 218 | Ascomycota | Capnodiales | Teratosphaeriaceae | *Rachicladosporium monterosium** | 98%(464/472) | KC966122 | *Uncultured fungus clone*^§^ | Arctic soil in USA (69.67N; 148.72W) |
|  |  |  |  |  |  |  | 97%(353/365) | KF309940 | *Rachicladosporium monterosium*^§^ | Unreported |
| 363 | 2 | 61 | Ascomycota | Capnodiales | Unassigned | *Capnodiales sp.* | 99%(505/511) | KF297284 | *Uncultured fungus clone*^§^ | Arctic soil in Canada (73.22N,119.56W) |
|  |  |  |  |  |  |  | 89%(479/539) | GU570527 | *Devriesia pseudoamericana* | Fruit surface in Germany |
| 364 | 1 | 6 | Ascomycota | Lecanorales | Porpidiaceae | *Biatora carneoalbida** | 99%(528/530) | KC965547 | *Uncultured fungus clone*^§^ | Arctic soil in Canada (76.23N; 119.30W) |
|  |  |  |  |  |  |  | 99%(520/524) | AJ247567 | *Biatora carneoalbida*^§^ | Finland |
| 365 | 1 | 13 | Basidiomycota | Auriculariales | Unassigned | *Oliveonia sp.* ^#^ | 96%(467/487) | FJ554256 | *Uncultured Agaricomycetes clone* | Forest soil in Canada |
|  |  |  |  |  |  |  | 95%(449/473) | HQ441577 | *Oliveonia pauxilla* | United Kingdom |
| 366 | 1 | 41 | Ascomycota | Capnodiales | Cladosporiaceae | *Cladosporium herbarum** | 100% (560/560) | HG798775 | *Cladosporium herbarum*^§^ | Unreported |
| 367 | 3 | 6 | Ascomycota | Helotiales | Helotiaceae | *Hymenoscyphus sp.* ^#^ | 99%(451/454) | KC966057 | *Uncultured fungus clone*^§^ | Arctic soil in USA (69.15N; 148.85W) |
|  |  |  |  |  |  |  | 97%(463/477) | AB705232 | *Hymenoscyphus sp.* ^§^ | Japan |
| 368 | 1 | 6 | Ascomycota | Helotiales | Helotiaceae | *Filosporella sp.* ^#^ | 98%(331/337) | KF617278 | *Uncultured fungus clone*^§^ | Soil in Alaska (64.9965N, 147.6530W) |
|  |  |  |  |  |  |  | 96%(324/336) | KC834047 | *Filosporella fistucella* | Plant roots |
| 369 | 1 | 14 | Unassigned | Unassigned | Unassigned | *Fungus sp.* | 93% (364/393) | AM999619 | *Uncultured fungus clone* | Bryphyte in Norway (Telemark) |
| 370 | 2 | 106 | Ascomycota | Unassigned | Unassigned | *Ascomycota sp.* | 98%(483/493) | KF297263 | *Uncultured fungus clone*^§^ | Arctic soil in Canada (76.23N; 119.30W) |
|  |  |  |  |  |  |  | 87%(406/483) | AF096208 | *Umbilicaria vellea* | Unreported |

*.For sequence similarity ≥ 97%, the genus and species were accepted;

#.For sequence similarity between 95% and 97%, only the genus was accepted;

§.For sequence similarity ≥ 97%, the matched fungal sequence and fungal OTU were considered as the same fungal taxa.

**Table S4 |** Information on the phylum, classes, orders, families, genera, and species of lichen-associated fungi identified in the present study.

| Phylum | Class | Order | Family | Genus | Species |
| --- | --- | --- | --- | --- | --- |
| Ascomycota  Basidiomycota  Zygomycota | Agaricomycetes  Agaricostilbomyces  Cystobasidiomycetes  Dothideomycetes  Eurotiomycetes  Exobasidiomycetes  Leotiomycetes  Lecanoromycetes  Pucciniomycetes  Microbotryomycetes  Saccharomycetes  Sordariomycetes  Tremellomycetes  Zygomycetes | Agaricales  Agaricostibales  Auriculariales  Baeomycetales  Boletales  Cantharellales  Capnodiales  Chaetothyriales  Cystofilobasidiales  Dothileales  Erythrobasidiales  Eurotiales  Filobasidiales  Helotiales  Hypocreales  Hysteriales  Lecanorales  Lecideales  Malasseziales  Microascales  Mortierellales Mytilinidiales  Pertusariales  Pezizales  Platygloeales  Pleosporales  Polyporales  Rhytismatales  Russulales  Saccharomycetales  Sebacinales  Sporidiobolales  Teloschistales  Thelebolales  Thelephorales  Trapeliales  Tremellales  Xylariales  Venturiales  Verrucariales | Amphisphaeriaceae  Apiosporaceae  Aspergillaceae  Baeomycetaceae  Boletaceae  Cladosporiaceae  Clavicipitaceae  Cystofilobasidiaceae  Dermeataceae  Dothidoraceae  Erythrobasidiaceae  Filobasidiaceae  Gloniaceae  Halosphaeriaceae  Helotaiceae  Herpotrichiellaceae  Hyaloscyphaceae  Lecanoraceae  Lecideaceae  Leotiaceae  Malasseziaceae  Megasporaceae  Melanommataceae  Mortierellaceae  Mycosphaerellaceae  Nectriaceae  Phaeosphaeriaceae  Pleosporaceae  Porpidiaceae  Pyronemataceae  Ramalinaceae  Rhytismataceae  Russulaceae  Saccharomycetaceae  Sclerotiniaceae  Sebacinaceae  Sporormiaceae  Stereocaulaceae  Strophariaceae  Syzygosporaceae  Teloschistaceae  Teratosphaeriaceae  Thelebolaceae  Thelephoraceae  Trapeliaceae  Tremellaceae  Tricholomataceae  Tulasnellaceae  Ventruiaceae  Verrucariaceae  Vibrisseaceae | *Acremonium*  *Alatospora*  *Alternaria*  *Arrhenia*  *Arthrinium*  *Aspergillus*  *Atla*  *Atradidymella*  *Baeomyces*  *Biatora*  *Bilimbia*  *Boletellus*  *Cadophora*  *Capnobotryella*  *Capronia*  *Carbonea*  *Cenococcum*  *Cladophialophora*  *Cladosporium*  *Claussenomyces*  *Coniothyrium*  *Cryptococcus*  *Dermea*  *Devriesia*  *Dioszegia*  *Elasticomyces*  *Exophiala*  *Filosporella*  *Fusarium*  *Galerina*  *Geltingia*  *Godronia*  *Gorgomyces*  *Gymnopus*  *Herpotrichia*  *Hymenoscyphus*  *Hyphodiscus*  *Infundichalara*  *Itersonilia*  *Lecidea*  *Malassezia*  *Monodictys*  *Mortierella*  *Mrakia*  *Mycoarthris*  *Mycocentrospora*  *Oidiodendron*  *Oleoguttula*  *Oliveonia*  *Paryoplaca*  *Penidiella*  *Pezizella*  *Phaeosphaeria*  *Phialocephala*  *Pichia*  *Pochonia*  *Polyblastia*  *Porpidia*  *Preussia*  *Rachicladosporium*  *Rhinocladiella*  *Rhizoscyphus*  *Rhodotorula*  *Russula*  *Sclerotinia*  *Sebacina*  *Seimatosporium*  *Sporidiobolas*  *Sporodictyon*  *Tetracladium*  *Thelobolus*  *Tomentella*  *Trapeliopsis*  *Trechispora*  *Tremella*  *Trichosporon*  *Tricladium*  *Venturia* | *Alatospora acuminata*  *Alatospora flagellata*  *Arrhenia lobata*  *Arthrinium arundinis*  *Aspergillus niger*  *Aspicillia simoenosis*  *Atla alpina*  *Atradidymella muscivota*  *Baeomyces rufus*  *Biatora carneoalbida*  *Biatora subduples*  *Bilimbia microcarpa*  *Boletellus obscurococcineus*  *Cadophora luteo-olivacea*  *Capronia peltigerae*  *Cenococcum geophilum*  *Cladophialophora humicolae*  *Cladosporium cf. tenuissimum*  *Cladosporium herbarum*  *Coniothyrium fuckelii*  *Cryptococcus gilvescens*  *Cryptococcus tephrensis*  *Cryptococcus victoriae*  *Dermea viburni*  *Dioszegia fristingensis*  *Elasticomyces elasticus*  *Galerina unicolor*  *Geltingia associata*  *Godronia cassandrae*  *Gorgomyces honrubiae*  *Herpotrichia juniperi*  *Infundichalara microchona*  *Lecidea ementiens*  *Malassezia restricta*  *Monodictys arctica*  *Mrakia blollopis*  *Paryoplaca athallina*  *Pezizella discreta*  *Phaeosphaeria triglochinicola*  *Phialocephala flargerbergii*  *Pichia pastoris*  *Polyblastia inconspicua*  *Polyblastia sendtneri*  *Polyblastia terrestis*  *Polyblastia wheldonii*  *Porpidia macrocarpa*  *Rachicladosporium monterosium*  *Rhizoscyphus ericae*  *Rhodotorula lamellibrachiae*  *Sporodictyon schaererianum*  *Tetracladium furcatum*  *Thelobolus globosus*  *Trapeliopsis bisorediata*  *Trichosporon loubieri*  *Tricladium angulatum* |

**Table S5 |** A dissimilarity test (multiple response permutation procedure, MRPP) for significant differences in fungal communities among the 7 lichen species using QIIME 1.8.0 software.

|  | *Cetrariella delisei* | *Cladonia borealis* | *Cladonia arbuscula* | *Cladonia pocillum* | *Flavocetraria*  *nivalis* | *Ochrolechia frigida* | *Peltigera canina* | groups |
| --- | --- | --- | --- | --- | --- | --- | --- | --- |
| delta | 0.9561 | 0.9158 | 0.9511 | 0.9607 | 0.9086 | 0.9538 | 0.9614 | - |
| n^#^ | 4 | 3 | 2 | 5 | 3 | 4 | 3 | - |
| A-value | - | - | - |  |  |  |  | 0.02698 |
| Observed delta | - | - | - |  |  |  |  | 0.9459 |
| Expected delta | - | - | - |  |  |  |  | 0.9722 |
| Significance of delta^*^ | - | - | - |  |  |  |  | 0.001 |

# Number of samples in each lichen species

*Based on 999 permutations

**Table S6 |** Primers used for 454 pyrosequencing of the 24 lichen samples.

| Sample ID | Forward Primer (5’-adaptor A-barcode-ITS1F-3’) | Reverse Primer (5’-adaptor B-ITS4-3’) |
| --- | --- | --- |
| ZT2013175 | CCATCTCATCCCTGCGTGTCTCCGACGACT- GACTGTGT -CTTGGTCATTTAGAGGAAGTAA | CCTATCCCCTGTGTGCCTTGGCAGTCGACT-TCCTCCGCTTATTGATATGC |
| ZT2013030 | CCATCTCATCCCTGCGTGTCTCCGACGACT - GACTCTGA -CTTGGTCATTTAGAGGAAGTAA | CCTATCCCCTGTGTGCCTTGGCAGTCGACT-TCCTCCGCTTATTGATATGC |
| ZT2013096 | CCATCTCATCCCTGCGTGTCTCCGACGACT - GAGTAGTG -CTTGGTCATTTAGAGGAAGTAA | CCTATCCCCTGTGTGCCTTGGCAGTCGACT-TCCTCCGCTTATTGATATGC |
| ZT2013234 | CCATCTCATCCCTGCGTGTCTCCGACGACT - GAGAGTGT -CTTGGTCATTTAGAGGAAGTAA | CCTATCCCCTGTGTGCCTTGGCAGTCGACT-TCCTCCGCTTATTGATATGC |
| ZT2013128 | CCATCTCATCCCTGCGTGTCTCCGACGACT - CAGAGTGA -CTTGGTCATTTAGAGGAAGTAA | CCTATCCCCTGTGTGCCTTGGCAGTCGACT-TCCTCCGCTTATTGATATGC |
| ZT2013230 | CCATCTCATCCCTGCGTGTCTCCGACGACT - CATGTGCA -CTTGGTCATTTAGAGGAAGTAA | CCTATCCCCTGTGTGCCTTGGCAGTCGACT-TCCTCCGCTTATTGATATGC |
| ZT2013076 | CCATCTCATCCCTGCGTGTCTCCGACGACT -AGAGACTG-CTTGGTCATTTAGAGGAAGTAA | CCTATCCCCTGTGTGCCTTGGCAGTCGACT-TCCTCCGCTTATTGATATGC |
| ZT2013129 | CCATCTCATCCCTGCGTGTCTCCGACGACT - GTACTGCA -CTTGGTCATTTAGAGGAAGTAA | CCTATCCCCTGTGTGCCTTGGCAGTCGACT-TCCTCCGCTTATTGATATGC |
| ZT2013212 | CCATCTCATCCCTGCGTGTCTCCGACGACT - GTAGTGCT -CTTGGTCATTTAGAGGAAGTAA | CCTATCCCCTGTGTGCCTTGGCAGTCGACT-TCCTCCGCTTATTGATATGC |
| ZT2013155 | CCATCTCATCCCTGCGTGTCTCCGACGACT - CAGTGTGT -CTTGGTCATTTAGAGGAAGTAA | CCTATCCCCTGTGTGCCTTGGCAGTCGACT-TCCTCCGCTTATTGATATGC |
| ZT2013231 | CCATCTCATCCCTGCGTGTCTCCGACGACT - CGATGCAT -CTTGGTCATTTAGAGGAAGTAA | CCTATCCCCTGTGTGCCTTGGCAGTCGACT-TCCTCCGCTTATTGATATGC |
| ZT2013211 | CCATCTCATCCCTGCGTGTCTCCGACGACT - CATCGTAG -CTTGGTCATTTAGAGGAAGTAA | CCTATCCCCTGTGTGCCTTGGCAGTCGACT-TCCTCCGCTTATTGATATGC |
| ZT2013125 | CCATCTCATCCCTGCGTGTCTCCGACGACT - CAGTAGAG -CTTGGTCATTTAGAGGAAGTAA | CCTATCCCCTGTGTGCCTTGGCAGTCGACT-TCCTCCGCTTATTGATATGC |
| ZT2013209 | CCATCTCATCCCTGCGTGTCTCCGACGACT - CACTGTGA -CTTGGTCATTTAGAGGAAGTAA | CCTATCCCCTGTGTGCCTTGGCAGTCGACT-TCCTCCGCTTATTGATATGC |
| ZT2013204 | CCATCTCATCCCTGCGTGTCTCCGACGACT - CTGAGTGT -CTTGGTCATTTAGAGGAAGTAA | CCTATCCCCTGTGTGCCTTGGCAGTCGACT-TCCTCCGCTTATTGATATGC |
| ZT2013246 | CCATCTCATCCCTGCGTGTCTCCGACGACT - CTGTAGTG -CTTGGTCATTTAGAGGAAGTAA | CCTATCCCCTGTGTGCCTTGGCAGTCGACT-TCCTCCGCTTATTGATATGC |
| ZT2013235 | CCATCTCATCCCTGCGTGTCTCCGACGACT - CTCTCTGA -CTTGGTCATTTAGAGGAAGTAA | CCTATCCCCTGTGTGCCTTGGCAGTCGACT-TCCTCCGCTTATTGATATGC |
| ZT2013079 | CCATCTCATCCCTGCGTGTCTCCGACGACT - GAGTCTGT -CTTGGTCATTTAGAGGAAGTAA | CCTATCCCCTGTGTGCCTTGGCAGTCGACT-TCCTCCGCTTATTGATATGC |
| ZT2013083 | CCATCTCATCCCTGCGTGTCTCCGACGACT - GAGTGTGA -CTTGGTCATTTAGAGGAAGTAA | CCTATCCCCTGTGTGCCTTGGCAGTCGACT-TCCTCCGCTTATTGATATGC |
| ZT2013205 | CCATCTCATCCCTGCGTGTCTCCGACGACT - GATCTGCA -CTTGGTCATTTAGAGGAAGTAA | CCTATCCCCTGTGTGCCTTGGCAGTCGACT-TCCTCCGCTTATTGATATGC |
| ZT2013209o | CCATCTCATCCCTGCGTGTCTCCGACGACT - GATGTGCT -CTTGGTCATTTAGAGGAAGTAA | CCTATCCCCTGTGTGCCTTGGCAGTCGACT-TCCTCCGCTTATTGATATGC |
| ZT2013054 | CCATCTCATCCCTGCGTGTCTCCGACGACT - GCATGCAT -CTTGGTCATTTAGAGGAAGTAA | CCTATCCCCTGTGTGCCTTGGCAGTCGACT-TCCTCCGCTTATTGATATGC |
| ZT2013104 | CCATCTCATCCCTGCGTGTCTCCGACGACT - GCTACGAT -CTTGGTCATTTAGAGGAAGTAA | CCTATCCCCTGTGTGCCTTGGCAGTCGACT-TCCTCCGCTTATTGATATGC |
| ZT2013198 | CCATCTCATCCCTGCGTGTCTCCGACGACT - GCTATACG -CTTGGTCATTTAGAGGAAGTAA | CCTATCCCCTGTGTGCCTTGGCAGTCGACT-TCCTCCGCTTATTGATATGC |
